# *SU*(2) Decomposition for the Quantum Information Dynamics in 2*d*-Partite Two-Level Quantum Systems

**DOI:** 10.3390/e20080610

**Published:** 2018-08-17

**Authors:** Francisco Delgado

**Affiliations:** Escuela de Ingeniería y Ciencias, Tecnológico de Monterrey, Atizapán 52926, Mexico; fdelgado@itesm.mx; Tel.: +52-55-5864-5670

**Keywords:** quantum information, quantum dynamics, entanglement

## Abstract

The gate array version of quantum computation uses logical gates adopting convenient forms for computational algorithms based on the algorithms classical computation. Two-level quantum systems are the basic elements connecting the binary nature of classical computation with the settlement of quantum processing. Despite this, their design depends on specific quantum systems and the physical interactions involved, thus complicating the dynamics analysis. Predictable and controllable manipulation should be addressed in order to control the quantum states in terms of the physical control parameters. Resources are restricted to limitations imposed by the physical settlement. This work presents a formalism to decompose the quantum information dynamics in $SU(2^{2d})$ for 2d-partite two-level systems into $2^{2d-1}$ SU(2) quantum subsystems. It generates an easier and more direct physical implementation of quantum processing developments for qubits. Easy and traditional operations proposed by quantum computation are recovered for larger and more complex systems. Alternating the parameters of local and non-local interactions, the procedure states a universal exchange semantics on the basis of generalized Bell states. Although the main procedure could still be settled on other interaction architectures by the proper selection of the basis as natural grammar, the procedure can be understood as a momentary splitting of the 2d information channels into $2^{2d-1}$ pairs of 2 level quantum information subsystems. Additionally, it is a settlement of the quantum information manipulation that is free of the restrictions imposed by the underlying physical system. Thus, the motivation of decomposition is to set control procedures easily in order to generate large entangled states and to design specialized dedicated quantum gates. They are potential applications that properly bypass the general induced superposition generated by physical dynamics.

## 1. Introduction

Quantum information is generating new applications and tentative future technologies such as quantum computation [1,2,3] and quantum cryptography, based on disruptive phenomena in its main trends: quantum key distribution [4,5], quantum secret sharing [6], and quantum secure direct communication [7,8]. All these trends highlight the importance of entangled states—a basic aspect involved in the current work in order to achieve quantum information processing tasks. In this arena, the understanding of quantum information dynamics and the control of quantum systems is a compulsory development to manage the quantum resources involved. Applications require a tight control of resources and interactions—especially those related with coherence and entanglement. They are fundamental in most applications. Quantum control has developed the fine management of physical variables to prepare, maintain, and transform quantum states in order to exploit them for concrete purposes. The outstanding high-tech commercial appliances D-Wave and IBM-Q use qubits in the form of two-level systems, either with superconducting circuits or ions as well as several approaches to their interconnecting architecture.

For multipartite systems, research in control is oriented to achieve different goals in quantum applications. Most of them are numerical approaches rather than analytical due to the inherited complexity in the quantum information dynamics when the number of parts grows. For a single system with a two-level spectrum, the control problem has been extensively studied in terms of exact optimal control for energy or time cost [9,10]. Recently, research of the anisotropic Heisenberg–Ising model for bipartite systems in SU(4) [11] has shown how this model exhibits SU(2) block decomposition when it is written in the non-local basis of Bell states instead of the traditional computational basis. This means that $H^{2}$ becomes a direct sum of two subspaces, each one generated by a pair of Bell states, while *U* underlies in the direct product $U\left(1\right)\times SU{\left(2\right)}^{2}$. Thus, control can be reduced to SU(2) control problems, each one in each block. Then, exact solutions for some control procedures can be found [12,13]. There, controlled blocks can be configured by the direction of external driven interactions introduced. That scheme allows controlled transformations between Bell states on demand and therefore on a general state. Thus, the procedure sets a method of control to manipulate quantum information on magnetic systems, where the computational grammar is based on Bell states instead of the traditional computational basis. It allows an easier programmed transformation among any pair of elements in that basis. This result provided the inspiration to reproduce similar decomposition schemes for larger systems in terms of simpler problems based on quasi-isolated two-level subsystems, developing easier and universal (not necessarily optimal) controlled manipulation procedures for quantum information. Technology to set up the possible architecture of these generic systems is currently being achieved through trapped-ion qubits [14] and superconducting qubits [15].

Thus, the generalization of SU(2) block decomposition is a convenient formalism to express dynamics, revealing certain quantum information states algebraically free of the complexity introduced by the entangling operations (doing few convenient the use of the computational basis). Nevertheless, they still conserve their entangled properties. This reveals how the probability exchange happens together with the structure of entanglement behind the randomness introduced by the complexity of large quantum information systems. Still, as for their predecessor, those bases maintain a certain degree of universality, including several alternative local and non-local interactions. As for their SU(4) predecessor, when they are combined, it states a series of punctual operations that can be set: (a) fine control based on well-known SU(2) control procedures; (b) the construction of universal gates for the entire process based on two-channel like operations; and (c) the design of more complex dedicated multi-channel gates by factorization.

The general aim of this paper is to show that such decomposition and reduction is achievable for large qubit systems, not only for those in [11,13]. The second section states the general Hamiltonian to be analyzed. The third section shows how the SU(2) decomposition procedure can be generalized on general *n*-partite two-level systems (not only for the driven Heisenberg–Ising interactions), reducing them to $2^{n-1}$ selectable transformations between pairs of specific quantum energy states. Then, these transformations can be based on known control schemes for SU(2) systems such as those in [9,10]. The selection of these $2^{n-1}$ pairs of states can be based on the convenience of the quantum process being considered and the resources involved. Thus, the basis on which the decomposition can be established works as a computational grammar for the quantum procedures being attained. These bases are not completely arbitrary, and thus the fourth section shows how a kind of generalized Bell basis is able to generate the SU(2) decomposition for an even number of parts, n=2d. The fifth section is devoted to analyzing the restrictions on the Hamiltonian to get the SU(2) decomposition, the inherited states, and the block properties. This analysis includes a classification of interactions able to generate the SU(2) decomposition. Because the presented procedure can reproduce complex quantum gates, generate large entangled states, and introduce control procedures in $SU(2^{2d})$ if the grammar is based on the proposed basis, the sixth section analyzes potential applications in these trends. The final section concludes, summarizing the findings and settling the related future work to be developed. Because several aspects in the work may be complex for the reader, an appendix with some critical concepts has been included to clarify the contents.

## 2. Generalized Hamiltonian

The problem can be established for a general Hamiltonian for *n* coupled two-level systems on $U(2^{n})$ forming a closed system. It can be written as a general combination of tensor products of Pauli matrices for each subsystem (for a more detailed discussion of this Hamiltonian, please see Appendix A.1):

(1) $$\begin{array}{c} \tilde{H}=\sum_{\left\{i_{k}\right\}}h_{\left\{i_{k}\right\}}\overset{n}{\underset{k=1}{⨂}}\sigma_{i_{k}}=\sum_{I=0}^{4^{n}-1}h_{I_{4}^{n}}\overset{n}{\underset{k=1}{⨂}}\sigma_{I_{4,k}^{n}}, \end{array}$$

where $\left\{i_{k}\right\}=\left\{i_{1},i_{2},\ldots ,i_{n}\right\}$, $i_{k}=0,1,2,3$, and $h_{\left\{i_{k}\right\}}$ is a general set of time-dependent real functions. Sometimes, as in the second expression in (1), $\left\{i_{k}\right\}$ will be represented as the number $I\in \{0,1,\ldots ,4^{n}\;-\;1\}$, as it is expressed in base-4 with *n* digits, $I_{4}^{n}$. Then, $I_{4,k}^{n}=i_{k}$ for k=1,2,…,n. $\sigma_{i_{k}}$ for $i_{k}=0,1,2,3$ are the traditional Pauli matrices in the computational basis $|0\rangle ,|1\rangle \in H^{2}$ for each part *k*. Note that due to the SU(2) algebra of Pauli matrices, this Hamiltonian comprises all analytical Hamiltonians based on two-level systems with *n* parts. The Hamiltonian obeys the Schrödinger equation for its associated evolution operator $\tilde{U}$:

(2) $$\begin{array}{c} \tilde{H}\tilde{U}=i\hbar \frac{\partial \tilde{U}}{\partial t}. \end{array}$$

Although $h_{\left\{0,\ldots ,0\right\}}$ is not necessarily zero, if $\left\{{\tilde{E}}_{j}\;|j=1,\ldots ,2^{n}\right\}$ are the eigenvalues of $\tilde{H}$ and $E\;\equiv \;\sum_{j=1}^{2^{n}}{\tilde{E}}_{j}=2^{n}h_{\left\{0,\ldots ,0\right\}}$, then defining

(3) $$\begin{array}{c} H\equiv \tilde{H}-h_{\left\{0,\ldots ,0\right\}}\overset{n}{\underset{k=1}{⨂}}\sigma_{0},\;U\equiv \tilde{U}e^{\frac{i}{\hbar }h_{\left\{0,\ldots ,0\right\}}t}, \end{array}$$

these operators become the equivalent traceless Hamiltonian and its corresponding evolution operator with eigenvalues $E_{j}=\tilde{E_{j}}-h_{\left\{0,\ldots ,0\right\}}$, both fulfilling (2) as well. *H* and $\tilde{H}$ have the same set of eigenvectors $\left\{|b_{j}\rangle \in H^{2^{n}}|j=1,\ldots ,2^{n}\right\}$. Thus, the Hamiltonian can be written alternatively as $H=\sum_{j=1}^{2^{n}}E_{j}|b_{j}\rangle \langle b_{j}|$. Thus, in the following, the Hamiltonian can be assumed traceless without loss of generality. Note that while $\tilde{U}\in U\left(2^{n}\right)$, then $U\in SU(2^{n})$. In the following, only *H* and *U* symbols will be used as equivalent to $\tilde{H}$ and $\tilde{U}$. *H* can be split in two parts—the local $H_{l}$ and the non-local $H_{\mathrm{nl}}$ interactions:

(4) $$\begin{array}{c} H_{l}=\sum_{k=1}^{n}\sum_{i=1}^{3}h_{{\left(i4^{k-1}\right)}_{4}^{n}}\overset{n}{\underset{s=1}{⨂}}\sigma_{{\left(i4^{k-1}\right)}_{4,s}^{n}}\to \tilde{H}={\tilde{H}}_{\mathrm{nl}}+H_{l}, \end{array}$$

where ${\left(i4^{k-1}\right)}_{4}^{n}$ is the number $i4^{k-1}$ represented in base-4 with *n* digits and ${\left(i4^{k-1}\right)}_{4,s}^{n}$ is its $s^{\mathrm{th}}$ term in that basis.

## 3. SU(2) Decomposition Generalities

In order to support the understanding of some aspects in the further discussion, Appendix A.2 contains a brief of group theory that is relevant for this work as well as some critical points in the decomposition procedure being presented here. Delgado [11] found that the SU(2) decomposition procedure can be induced by considering a set of $2^{n}$ orthogonal states: $\left\{|\alpha_{i}\rangle \right\}$ and $2^{n-1}$ pairs $\left\{j\left(i\right),k\left(i\right)\right\},i=1,2,\ldots ,2^{n-1}$, with $k\left(i\right)=j\left(i\right)+1\in \left\{2,4,\ldots ,2^{n}\right\}$ related with the eigenvalues through a mixing matrix, in such way that they fulfill:

(5) $$\begin{array}{cccc} 2|b_{2i-1}\rangle & =A_{i}|\alpha_{j(i)}\rangle +B_{i}|\alpha_{k(i)}\rangle & \;\to \;|\alpha_{j(i)}\rangle & =A_{i}^{*}|b_{2i-1}\rangle -B_{i}e^{-i\phi }|b_{2i}\rangle ,\\ |b_{2i}\rangle & =-B_{i}^{*}e^{i\phi }|\alpha_{j(i)}\rangle +A_{i}^{*}e^{i\phi }|\alpha_{k(i)}\rangle & \;\to \;|\alpha_{k(i)}\rangle & =B_{i}^{*}|b_{2i-1}\rangle +A_{i}e^{-i\phi }|b_{2i}\rangle , \end{array}$$

with $|A_{i}{|}^{2}+{\left|B_{i}\right|}^{2}=1$, where last relations are clearly true because of orthogonality (note that energies $E_{j}$ become ordered as the states are paired). States $\left\{|\alpha_{j}\rangle \right\}$ are then defined by the selection of $A_{i},B_{i}$. Each pair sets one of the orthogonal subspaces:

(6) $$\begin{array}{c} H_{i}^{2}=\mathrm{span}\left(\left\{|b_{2i-1}\rangle ,|b_{2i}\rangle \right\}\right)=\mathrm{span}\left(\left\{|\alpha_{j(i)}\rangle ,|\alpha_{k(i)}\rangle \right\}\right)\to H^{2^{n}}=\overset{2^{n-1}}{\underset{i=1}{⨁}}H_{i}^{2}. \end{array}$$

There are many possibilities for this selection, but not necessarily all practical bases fit in this construction. In particular, separability or entanglement properties are not necessarily assured for $\left\{|\alpha_{i}\rangle \right\}$ as in [11]. Clearly, because these states are assumed unitary, then $A_{i}\;=\;\langle b_{2i}|\alpha_{k(i)}\rangle e^{i\phi }=\;{\langle b_{2i-1}|\alpha_{j(i)}\rangle }^{*},\;B_{i}={\langle b_{2i-1}|\alpha_{k(i)}\rangle }^{*}=-\langle b_{2i}|\alpha_{j(i)}\rangle e^{i\phi }$. By applying *H* on (5) and considering that $|b_{i}\rangle$ has the eigenvalue $E_{i}$, it is possible to arrive at the following expressions:

(7) $$\begin{array}{cc} H|\alpha_{j(i)}\rangle & =\left(\right|A_{i}{|}^{2}E_{2i-1}+|B_{i}{|}^{2}E_{2i})|\alpha_{j(i)}\rangle +A_{i}^{*}B_{i}\left(E_{2i-1}-E_{2i}\right)|\alpha_{k(i)}\rangle ,\\ H|\alpha_{k(i)}\rangle & =A_{i}B_{i}^{*}\left(E_{2i-1}-E_{2i}\right)|\alpha_{j(i)}\rangle +\left(\right|A_{i}{|}^{2}E_{2i}+|B_{i}{|}^{2}E_{2i-1})|\alpha_{k(i)}\rangle , \end{array}$$

giving the Hamiltonian components in this basis:

(8) $$\begin{array}{cc} \langle \alpha_{j(i)}\left|H\right|\alpha_{j(i)}\rangle & =|A_{i}{|}^{2}E_{2i-1}+{\left|B_{i}\right|}^{2}E_{2i},\\ \langle \alpha_{k(i)}\left|H\right|\alpha_{k(i)}\rangle & =|A_{i}{|}^{2}E_{2i}+{\left|B_{i}\right|}^{2}E_{2i-1},\\ \langle \alpha_{j(i)}\left|H\right|\alpha_{k(i)}\rangle & =A_{i}B_{i}^{*}\left(E_{2i-1}-E_{2i}\right), \end{array}$$

which can be alternatively obtained from (5). Note that the phase ϕ is non-physical. This basis transformation changes the diagonal structure for the basis $\left\{|b_{i}\rangle \right\}$ into a 2×2 diagonal block structure for the basis $\left\{|\alpha_{j}\rangle \right\}$. For simplicity, we define the following quantities:

(9) $$\begin{array}{cc} A_{i}=\; & r_{A_{i}}e^{i\gamma_{A_{i}}},B_{i}=r_{B_{i}}e^{i\gamma_{B_{i}}},\\ \Delta_{i}^{\pm }=\; & \frac{1}{2\hbar }\left(E_{2i}\pm E_{2i-1}\right),\\ \Gamma_{i}=\; & \gamma_{A_{i}}-\gamma_{B_{i}}. \end{array}$$

Then, each 2×2 block in *H* (labeled as ${S_{H}}_{i}$) can be written in matrix form as (see Appendix A.2):

(10) $$\begin{array}{ccc} {S_{H}}_{i} & = & \left(\begin{array}{cc} \Delta_{i}^{+}-\left({r_{A_{i}}}^{2}-{r_{B_{i}}}^{2}\right)\Delta_{i}^{-} & -2r_{A_{i}}r_{B_{i}}\Delta_{i}^{-}e^{i\Gamma_{i}}\\ -2r_{A_{i}}r_{B_{i}}\Delta_{i}^{-}e^{-i\Gamma_{i}} & \Delta_{i}^{+}+\left({r_{A_{i}}}^{2}-{r_{B_{i}}}^{2}\right)\Delta_{i}^{-} \end{array}\right)\\ & = & \Delta_{i}^{+}I_{i}-2r_{A_{i}}r_{B_{i}}\Delta_{i}^{-}\cos\Gamma_{i}X_{i}+2r_{A_{i}}r_{B_{i}}\Delta_{i}^{-}\sin\Gamma_{i}Y_{i}-\left({r_{A_{i}}}^{2}-{r_{B_{i}}}^{2}\right)\Delta_{i}^{-}Z_{i}\\ & \equiv & \Delta_{i}^{+}I_{i}+{S_{H}}_{i}^{0}, \end{array}$$

where although ${r_{A_{i}}}^{2}+{r_{B_{i}}}^{2}=1$, we use both terms $r_{A_{i}}$ and $r_{B_{i}}$ for the symmetry. In addition, $I_{i},X_{i},Y_{i}$, and $Z_{i}$ are respectively the 2×2 unitary matrix and the Pauli matrices settled as basis for the block ${S_{H}}_{i}$. Thus, *H* can be written as a sum of operators acting on the different subspaces $H_{i}^{2}$ or as the following direct sum structure of $2^{n-1}$ 2×2 block-diagonal matrices:

(11) $$\begin{array}{ccc} H & = & \overset{2^{n-1}}{\underset{i=1}{⨁}}{S_{H}}_{i}=\left(\begin{array}{cccc} {S_{H}}_{1} & 0 & \ldots & 0\\ 0 & {S_{H}}_{2} & \ldots & 0\\ \vdots & \vdots & \ddots & \vdots \\ 0 & 0 & \ldots & {S_{H}}_{2^{n-1}} \end{array}\right), \end{array}$$

with 0, the 2×2 zero matrix. Because this structure is preserved under matrix products, it is inherited by the evolution matrix *U*. In particular, if the Hamiltonian (1) is not time-dependent, then $U\;=\;\sum_{j=1}^{2^{n}}e^{-\frac{i}{\hbar }E_{j}t}|b_{j}\rangle \langle b_{j}|$. Thus, when the basis is changed (see Appendix A.2):

(12) $$\begin{array}{cc} {S_{U}}_{i}=\; & e^{-\frac{i}{\hbar }E_{2i-1}t}|b_{2i-1}\rangle \langle b_{2i-1}|+e^{-\frac{i}{\hbar }E_{2i}t}|b_{2i}\rangle \langle b_{2i}|\\ =\; & e^{-i\Delta_{i}^{+}t}(\left(e^{i\Delta_{i}^{-}t}-2i{r_{B_{i}}}^{2}\sin\Delta_{i}^{-}t\right)|\alpha_{j}\left(i\right)\rangle \langle \alpha_{j}\left(i\right)|+\\ & 2ir_{A_{i}}r_{B_{i}}\sin\Delta_{i}^{-}t\left(e^{i\Gamma_{i}}|\alpha_{j}\left(i\right)\rangle \langle \alpha_{k}\left(i\right)|+e^{-i\Gamma_{i}}|\alpha_{k}\left(i\right)\rangle \langle \alpha_{j}\left(i\right)|\right)+\\ & \left(e^{-i\Delta_{i}^{-}t}+2i{r_{B_{i}}}^{2}\sin\Delta_{i}^{-}t\right)|\alpha_{k}\left(i\right)\rangle \langle \alpha_{k}\left(i\right)|). \end{array}$$

Similarly, in matrix form or in terms of $I_{i},X_{i},Y_{i}$, and $Z_{i}$:

(13) $$\begin{array}{cc} {S_{U}}_{i}=\; & e^{-i\Delta_{i}^{+}t}\left(\begin{array}{cc} \cos\Delta_{i}^{-}t+i\left({r_{A_{i}}}^{2}-{r_{B_{i}}}^{2}\right)\sin\Delta_{i}^{-}t & 2ir_{A_{i}}r_{B_{i}}e^{i\Gamma_{i}}\sin\Delta_{i}^{-}t\\ 2ir_{A_{i}}r_{B_{i}}e^{-i\Gamma_{i}}\sin\Delta_{i}^{-}t & \cos\Delta_{i}^{-}t-i\left({r_{A_{i}}}^{2}-{r_{B_{i}}}^{2}\right)\sin\Delta_{i}^{-}t \end{array}\right)\\ =\; & e^{-i\Delta_{i}^{+}t}(\cos\Delta_{i}^{-}tI_{i}+2ir_{A_{i}}r_{B_{i}}\cos\Gamma_{i}\sin\Delta_{i}^{-}tX_{i}-\\ & \;\;\;\;2ir_{A_{i}}r_{B_{i}}\sin\Gamma_{i}\sin\Delta_{i}^{-}tY_{i}+i\left({r_{A_{i}}}^{2}-{r_{B_{i}}}^{2}\right)\sin\Delta_{i}^{-}tZ_{i})\equiv e^{-i\Delta_{i}^{+}t}{S_{U}}_{i}^{0}. \end{array}$$

Note that the election of $\Gamma_{i}$ lets us simplify the last expression to contain only one operator between $X_{i}$ and $Y_{i}$ (as in [11,12]). This property is useful to set the optimal control in [9] in each block. Then, similar to *H*:

(14) $$\begin{array}{ccc} U & = & \overset{2^{n-1}}{\underset{i=1}{⨁}}{S_{U}}_{i}=\left(\begin{array}{cccc} {S_{U}}_{1} & 0 & \ldots & 0\\ 0 & {S_{U}}_{2} & \ldots & 0\\ \vdots & \vdots & \ddots & \vdots \\ 0 & 0 & \ldots & {S_{U}}_{2^{n-1}} \end{array}\right), \end{array}$$

where in general for the time-dependent Hamiltonian:

(15) $$\begin{array}{ccc} {S_{U}}_{i} & = & \tau \left\{e^{-\frac{i}{\hbar }\int_{0}^{t}{S_{H}}_{i}}dt^{\prime }\right\}=e^{-i\Delta_{i}^{+}t}\tau \left\{e^{-\frac{i}{\hbar }\int_{0}^{t}{S_{H}}_{i}^{0}}dt^{\prime }\right\}\equiv e^{-i\Delta_{i}^{+}t}{S_{U}}_{i}^{0}, \end{array}$$

where τ is the time-ordered integral. This implies that *U* is an element in the direct product $U{\left(1\right)}^{2^{n-1}-1}\;\times \;SU{\left(2\right)}^{2^{n-1}}\;\subset \;SU\left(2^{n}\right)$ (because any factor phase $e^{-i\Delta_{i}^{+}t}$ depends on the remaining phase factors through E, see Appendix A.2). In the following, we will informally call this factorization the SU(2) decomposition (in reality, each block has the form U(1)×SU(2)) due to the block structure. Consequently, the Hilbert space $H^{n}$ becomes the direct sum of $2^{n-1}$ subspaces generated by each pair $\left\{|\alpha_{j(i)}\rangle ,|\alpha_{k(i)}\rangle \right\}$, $i=1,2,\ldots ,2^{n-1}$. In each subspace, dynamics mixes the probabilities, but probabilities among subspaces remain unmixed if there is no rearrangement in the pairing between $\left\{|b_{i}\rangle \right\}$ and $\left\{|\alpha_{j}\rangle \right\}$ (clearly, in this rearrangement, the basis $\left\{|\alpha_{j}\rangle \right\}$ could change). Thus, if $|\psi_{0}\rangle \;=\;\sum_{i=1}^{2^{n-1}}|{\psi_{0}}_{i}\rangle$ is the initial state with $|{\psi_{0}}_{i}\rangle ={\psi_{0}}_{i,j(i)}|\alpha_{j(i)}\rangle +{\psi_{0}}_{i,k(i)}|\alpha_{k(i)}\rangle$, then each component is evolved in the subspace $i=1,2,\ldots ,2^{n-1}$, fulfilling $∥|{\psi_{t}}_{i}\rangle ∥=∥{S_{U}}_{i}^{0}|{\psi_{0}}_{i}\rangle ∥=∥|{\psi_{0}}_{i}\rangle ∥$.

Finally, note that the SU(2) decomposition is not the only one available, although it is the most valuable for the binary inheritance from the classical computation. In fact, other decompositions involving bigger subgroups are possible, whether using bigger systems than two-level ones and/or simply involving more than two eigenvectors in (5). Inclusively, a mixed-sized block structure can be realized.

## 4. GBS: A Non-Local Basis Fitting in {$|\alpha_{j}\rangle$}

Non-local bases are used as a theoretical resource to explicitly show how evolution [16] and measurement [17] can generate entangled states. In [11], it was shown that the Heisenberg–Ising model including driven magnetic fields in a fixed direction allows the generation of the block structure in the traditional Bell basis. Thus, the Bell basis for two-level bipartite systems has been shown to fit in the $U\left(1\right)\times SU{\left(2\right)}^{2}$ decomposition of SU(4). Despite the added complexity to manage non-local states, recent work has moved towards the control of entangled states [18]. This basis works as a universal basis for the Heisenberg–Ising interaction, including an external magnetic field in any specific direction on a couple of qubits [11,12,13]. This model includes other interaction models, such as XXX [19], XY [20], and XXZ [21]. In the current development, the most obvious guess is the generalized Bell states (GBS) basis for n=2d presented in [22] as tensor products of Bell states. In the next sections, some further useful formulas are obtained to show how the GBS basis fits in the SU(2) decomposition for larger systems than bipartite ones.

### 4.1. GBS Basis and Hamiltonian Components

For n=2d, the GBS basis [22] forms an orthogonal basis of partial entangled states for 2d particles. A more extended treatment for this basis is given in Appendix A.3 in order to ease further understanding in the current context, particularly related with the underlying single Bell states in their construction together with their index notation—a key aspect in the remaining development. Each element in this basis can be written briefly as:

(16) $$\begin{array}{ccc} |\Psi_{I_{4}^{d}}\rangle & = & \overset{d}{\underset{s=1}{⨂}}\frac{1}{\sqrt{2}}\sum_{\epsilon_{s},\delta_{s}=0}^{1}{\left({\tilde{\sigma }}_{i_{s}}\right)}_{\epsilon_{s},\delta_{s}}|\epsilon_{s}\delta_{s}\rangle \\ & = & \frac{1}{\sqrt{2^{d}}}\sum_{\left\{\epsilon_{j}\right\},\left\{\delta_{k}\right\}}{\left({\tilde{\sigma }}_{i_{1}}⊗\ldots ⊗{\tilde{\sigma }}_{i_{d}}\right)}_{\epsilon_{1}\ldots \epsilon_{d},\delta_{1}\ldots \delta_{d}}|\epsilon_{1}\ldots \epsilon_{d}\rangle ⊗|\delta_{1}\ldots \delta_{d}\rangle \\ & = & \frac{1}{\sqrt{2^{d}}}\sum_{E,D=0}^{2^{d}-1}{\left({\tilde{\sigma }}_{i_{1}}⊗\ldots ⊗{\tilde{\sigma }}_{i_{d}}\right)}_{E_{2}^{d},D_{2}^{d}}|E_{2}^{d}\rangle ⊗|D_{2}^{d}\rangle , \end{array}$$

where $\left\{\epsilon_{j}\right\}=\left\{\epsilon_{1},\ldots ,\epsilon_{d}\right\},\left\{\delta_{k}\right\}=\left\{\delta_{1},\ldots ,\delta_{d}\right\};\epsilon_{j},\delta_{k}=0,1$. At this point, ${\tilde{\sigma }}_{i}$ can be considered as proportionally unitary to the traditional Pauli matrices [22]. In addition, $I_{4}^{d}$ is a brief expression of $\left\{i_{1},i_{2},\ldots ,i_{d}\right\}$ as the digits set of $I\in \{0,1,\ldots ,4^{d}-1\}$ when it is written in base-4 with *d* digits. Similarly, $E_{2}^{d},D_{2}^{d}$ are numbers written in base-2 with *d* digits ($E,D\in \{0,1,\ldots ,2^{d}-1\})$ representing $\left\{\epsilon_{1},\ldots ,\epsilon_{d}\right\},\left\{\delta_{1},\ldots ,\delta_{d}\right\}$, respectively (note that digits are used inverted, as they commonly appear in $E_{2}^{d}$ or $I_{4}^{d}$ expressions). In the following, for simplicity, we use $I_{b}^{d}$ and I interchangeably because the base *b* can normally be inferred from the context. Each element in this basis is not maximally entangled. Instead, they have maximally entangled bipartite subsystems (see Appendix A.3), which are separable from the remaining system. Separable pairs contain the parts [s,s+d], s=1,2,…,d (in the following, square brackets will be used to point out a subsystem of parts in the whole system).

In order for $\left\{|\Psi_{I_{4}^{d}}\rangle \right\}$ ($I\in \{0,1,\ldots ,4^{d}-1\}$) to reach the kind of sets $\left\{|\alpha_{j}\rangle \right\}$ stated in the previous section where *H* and *U* achieve the SU(2) block structure, *H* should fulfill some restrictions. We are interested in setting these in the current subsections. Combining expressions (1) and (16), we can express the components of *H* in the GBS basis. First, we note [23]:

(17) $$\begin{array}{cc} \langle \Psi_{I_{4}^{d}}\left|\sigma_{j_{1}}⊗\ldots ⊗\sigma_{j_{2d}}\right|\Psi_{K_{4}^{d}}\rangle & =\prod_{s=1}^{d}\frac{1}{\sqrt{2}}\sum_{\epsilon_{s},\delta_{s}=0}^{1}{\left({\tilde{\sigma }}_{i_{s}}^{*}\right)}_{\epsilon_{s},\delta_{s}}\frac{1}{\sqrt{2}}\sum_{\gamma_{s},\phi_{s}=0}^{1}{\left({\tilde{\sigma }}_{k_{s}}\right)}_{\gamma_{s},\phi_{s}}\langle \epsilon_{s}\left|\sigma_{j_{s}}\right|\gamma_{s}\rangle \langle \delta_{s}\left|\sigma_{j_{s+d}}\right|\phi_{s}\rangle \\ \;\; & =\frac{1}{2^{d}}\sum_{\begin{array}{c} E,D\\ F,G \end{array}}{\left({\tilde{\sigma }}_{i_{1}}^{*}⊗\ldots ⊗{\tilde{\sigma }}_{i_{d}}^{*}\right)}_{E_{2}^{d},D_{2}^{d}}{\left(\sigma_{j_{1}}⊗\ldots ⊗\sigma_{j_{2d}}\right)}_{E_{2}^{d}D_{2}^{d},F_{2}^{d}G_{2}^{d}}{\left({\tilde{\sigma }}_{k_{1}}⊗\ldots ⊗{\tilde{\sigma }}_{k_{d}}\right)}_{F_{2}^{d},G_{2}^{d}}\\ \;\; & =\frac{1}{2^{d}}\prod_{s=1}^{d}\mathrm{Tr}\left({\tilde{\sigma }}_{i_{s}}^{*}\sigma_{j_{d+s}}{\tilde{\sigma }}_{k_{s}}^{T}\sigma_{j_{s}}^{T}\right), \end{array}$$

where combined subscripts as $E_{2}^{d}D_{2}^{d}$ represent the set of subscripts obtained by merging $\left\{\epsilon_{1}\ldots \epsilon_{d}\right\}$ and $\left\{\delta_{1}\ldots \delta_{d}\right\}$. Therefore, the final and notable expression for the Hamiltonian components becomes [23]:

(18) $$\begin{array}{ccc} \langle \Psi_{I_{4}^{d}}\left|H\right|\Psi_{K_{4}^{d}}\rangle & = & \frac{1}{2^{d}}\sum_{J=0}^{4^{2d}-1}h_{J_{4}^{2d}}\prod_{s=1}^{d}\mathrm{Tr}\left({\tilde{\sigma }}_{i_{s}}^{*}\sigma_{j_{d+s}}{\tilde{\sigma }}_{k_{s}}^{T}\sigma_{j_{s}}^{T}\right), \end{array}$$

where $J\in \{0,1,\ldots ,4^{2d}-1\}$ (here, J=0 can be removed in spite of the discussion in the first section). In the last expressions, the product ${\tilde{\sigma }}_{i_{s}}^{*}\sigma_{j_{d+s}}{\tilde{\sigma }}_{k_{s}}^{T}\sigma_{j_{s}}^{T}$ has some properties inherited from Pauli matrices. Because $\sigma_{1},\sigma_{2},\sigma_{3}$ are traceless and $\sigma_{i}^{T}=\pm \sigma_{i}$ (negative sign only if i=2), then $\mathrm{Tr}({\tilde{\sigma }}_{i_{s}}^{*}\sigma_{j_{d+s}}{\tilde{\sigma }}_{k_{s}}^{T}\sigma_{j_{s}}^{T})$ is non-zero only if $i_{s},j_{d+s},k_{s},j_{s}$ are: (a) completely different between them; or (b) equal by pairs.

A remark is convenient at this point. In some works (e.g., [22]), GBS are preferred to be defined using ${\tilde{\sigma }}_{i}=\sigma_{i}$ for i=0,1,3 and ${\tilde{\sigma }}_{2}=i\sigma_{2}$ because it allows real coefficients when they are expressed in the computational basis $|0\rangle ,|1\rangle$ (alternative definitions introduce specific phase factors in ${\tilde{\sigma }}_{i}$). We adopt the last definition in the following, which does not produce changes in the previous discussion. Note that ${\tilde{\sigma_{i}}}^{*}=\sigma_{i}^{T}=\sigma_{i}$. The last expression should be fitted to (11), in particular with the non-diagonal block entries. In the following sections, we will show that the GBS basis naturally generates the SU(2) decomposition if the Hamiltonian fulfills certain restrictions. The use of the GBS basis allows the management of this analysis because it is based on Pauli matrices.

### 4.2. Case d=1

For d=1 there are three possibilities to arrange the pairs in the corresponding GBS basis (reduced in this case to the traditional Bell states: $\left\{|\beta_{00}\rangle ,|\beta_{01}\rangle ,|\beta_{10}\rangle ,|\beta_{11}\rangle \right\}$). A direct but large analysis shows that by fitting (18) to (11), the Hamiltonian should be reduced to the forms shown in Table 1 (assuming always $h_{0_{4}^{2d}}=0$ and $H_{0}=\sum_{j=1}^{3}h_{jj}\sigma_{j}⊗\sigma_{j}$). The first column shows the pairs arrangement to construct the blocks. These results generalize those found in [11,12] for the anisotropic Heisenberg–Ising model reached if the crossed interaction terms such as $h_{ij}\sigma_{i}⊗\sigma_{j}$ with i,j=1,2,3;i≠j are not present. These terms are similar to the Dzyaloshinskii–Moriya model [24,25], opening additional possibilities for control in the pair exchange. Case d=1 is special in the current context because for d>1 crossed terms can be present only for a unique pair in order to keep the SU(2) decomposition.

Although the eigenvalues $\left\{E_{j}\right\}$ do not follow a specific order, expressions in (18) can be arranged in several orders as functions of the pairs selected $\left\{|\alpha_{j(i)}\rangle ,|\alpha_{k(i)}\rangle \right\}$, being related with the decomposition process. In general, there are $\frac{(2^{2d})!}{\left(2^{2d-1}\right)!2^{2^{2d-1}}}$ combinations for these pairs, which grow very quickly with *d* (3 for d=1; 2,027,025 for d=2, etc.), making the cases for d>1 unmanageable in an analogous direct analysis.

### 4.3. Case d>1

The exponential growth of the problem with *d* makes an exhaustive analysis for d>1 based on a large algebraic equation system impossible, as in the previous case. The previous case and the results in [11,12] suggest some possible Hamiltonians for more complex cases. Thus, some of the following forms (see Appendix A.1) could allow the SU(2) decomposition for the basis (16):

(19) $$\begin{array}{ccc} H_{0}=\sum_{j=1}^{3}H_{0}^{\left(j\right)}, & & H_{0}^{\left(j\right)}=h_{{\left(j\frac{4^{2d}-1}{3}\right)}_{4}^{2d}}\sigma_{j}^{⊗2d}, \end{array}$$

(20) $$\begin{array}{ccc} H_{{\mathrm{nl}}_{i}}=\sum_{k^{\prime }>k=1}^{2d}H_{{\mathrm{nl}}_{i}}^{\left(k,k^{\prime }\right)}, & & H_{{\mathrm{nl}}_{i}}^{\left(k,k^{\prime }\right)}=h_{{\left(i\left(4^{k-1}+4^{k^{\prime }-1}\right)\right)}_{4}^{2d}}\overset{2d}{\underset{s=1}{⨂}}\sigma_{{\left(i\left(4^{k-1}+4^{k^{\prime }-1}\right)\right)}_{4,s}^{2d}}, \end{array}$$

(21) $$\begin{array}{ccc} H_{{\mathrm{cnl}}_{i}}=\sum_{k^{\prime }>k=1}^{2d}H_{{\mathrm{cnl}}_{i}}^{\left(k,k^{\prime }\right)}, & & H_{{\mathrm{cnl}}_{i}}^{\left(k,k^{\prime }\right)}=\sum_{p=0}^{1}h_{{\left(j_{p}4^{k-1}+k_{p}4^{k^{\prime }-1}\right)}_{4}^{2d}}\overset{2d}{\underset{s=1}{⨂}}\sigma_{{\left(j_{p}4^{k-1}+k_{p}4^{k^{\prime }-1}\right)}_{4,s}^{2d}}, \end{array}$$

(22) $$\begin{array}{ccc} H_{l_{i}}=\sum_{k=1}^{2d}H_{l_{i}}^{\left(k\right)}, & & H_{l_{i}}^{\left(k\right)}=h_{{\left(i4^{k-1}\right)}_{4}^{2d}}\overset{2d}{\underset{s=1}{⨂}}\sigma_{{\left(i4^{k-1}\right)}_{4,s}^{2d}}, \end{array}$$

where ${\left(i4^{k-1}\right)}_{4}^{2d}$ is the base-4 representation with 2d digits of $i4^{k-1}$, a number with only one *i* in position *k* and zero in the other; ${\left(i4^{k-1}\right)}_{4,s}^{2d}$ is its element *s*; and ${\left(j\frac{4^{2d}-1}{3}\right)}_{4}^{2d}$ is the base-4 representation with 2d digits of $j\frac{4^{2d}-1}{3}$, a number with *j* in each digit position (by using the geometric partial sums properties). Note that i∈{1,2,3} is fixed in all expressions. Physically, $H_{0}$ represents a full simultaneous interaction between all particles (as in the bipartite Heisenberg–Ising anisotropic interaction). Although this kind of interaction is non-physical for d>1, it is included here for reference. $H_{{\mathrm{nl}}_{i}}$ represents the interaction between the component *i* of the spin for pairs of particles as in the Heisenberg–Ising model. $H_{{\mathrm{cnl}}_{i}}$ is the crossed non-local interactions by pairs in the direction *i* (as those for d=1 in Table 1), a label used to characterize these interactions (as in the Dzyaloshinskii–Moriya model). Note that $i,j_{p},k_{p}$ is a permutation of 1,2,3 with parity p=0,1, even and odd, respectively. Finally, $H_{l_{i}}$ is the component *i* of the local interactions with $h_{{\left(i4^{k-1}\right)}_{4}^{2d}}$ as strengths (e.g., magnetic fields in the direction *i* for magnetic systems). These cases generalize the bipartite models presented in [11,12] and those found for d=1.

Some observations are useful at this point: (a) ${\tilde{\sigma }}_{i}=\alpha_{i}\sigma_{i}$, $\alpha_{i}\in \left\{1,i\right\}$; (b) $\sigma_{i}^{T}=\beta_{i}\sigma_{i}$, $\beta_{i}\in \left\{-1,1\right\}$; (c) $\sigma_{i}\sigma_{j}=\gamma_{i,j}\sigma_{j}\sigma_{i}$, $\gamma_{i,j}\in \left\{-1,1\right\}$. Thus, $2c_{j_{s},j_{d+s}}^{i_{s},k_{s}}\equiv \mathrm{Tr}\left({\tilde{\sigma }}_{i_{s}}\sigma_{j_{d+s}}{\tilde{\sigma }}_{k_{s}}^{T}\sigma_{j_{s}}^{T}\right)=\alpha_{i_{s}}\alpha_{k_{s}}\beta_{j_{s}}\beta_{k_{s}}\gamma_{k_{s}j_{s}}\gamma_{k_{s}i_{s}}\mathrm{Tr}\left(\sigma_{i_{s}}\sigma_{k_{s}}\sigma_{j_{d+s}}\sigma_{j_{s}}\right)\in \left\{0,\pm 2,\pm 2i\right\}$. We do not provide extensive formulas for the coefficients $\alpha_{i},\beta_{i},\gamma_{i,j},c_{j_{s},j_{d+s}}^{i_{s},k_{s}}$, but they are trivially constructed departing from the Pauli matrices properties.

At this point, a convenient definition is introduced for the following cases. We will say that two particles or parts, i,j, are *correspondents* if j=i+d, with i,j−d∈{1,2,…,d}. This means simply that one is in the same position of the first group of the Hamiltonian subscripts 1,2,…,d as the other is in the second group d+1,d+2,…,2d. Then, the analysis of $\langle \Psi_{I_{4}^{d}}\left|H_{0}\right|\Psi_{K_{4}^{d}}\rangle$, $\langle \Psi_{I_{4}^{d}}\left|H_{l_{i}}\right|\Psi_{K_{4}^{d}}\rangle$, $\langle \Psi_{I_{4}^{d}}\left|H_{{\mathrm{cnl}}_{i}}\right|\Psi_{K_{4}^{d}}\rangle$ and $\langle \Psi_{I_{4}^{d}}\left|H_{{\mathrm{nl}}_{i}}\right|\Psi_{K_{4}^{d}}\rangle$ is conducted with the following results.

#### 4.3.1. Analysis of $\langle \Psi_{I_{4}^{d}}\left|H_{0}\right|\Psi_{K_{4}^{d}}\rangle$

Because $J=j\frac{4^{2d}-1}{3}$ in (18), then $j_{d+s}=j_{s}=j\;\forall s=1,2,\ldots ,d$, implying $c_{j_{s},j_{d+s}}^{i_{s},k_{s}}\neq 0$ only if $i_{s}=k_{s}\;\forall s=1,2,\ldots ,d$. Thus, $H_{0}$ is diagonal in the GBS basis representation and each entry will contain the same three terms $h_{{\left(j\frac{4^{2d}-1}{3}\right)}_{4}^{2d}}$ for j=1,2,3, but each with diverse signs. Despite the similitude of $H_{0}$ with the bipartite case (d=1), for multipartite cases this interaction is non-physical, but it allows the main idea to be introduced and understood in the remaining analysis.

#### 4.3.2. Analysis of $\langle \Psi_{I_{4}^{d}}\left|H_{l_{i}}\right|\Psi_{K_{4}^{d}}\rangle$

The treatment for the remaining cases is compressed in the explanation of the current case. By first considering only an isolated term $H_{l_{i}}^{\left(k\right)}$ (in this case $J=i4^{k-1}$ for some i∈{1,2,3} and k=1,2,…,2d in (18)), then J in the base-4 representation contains only one *i* (in the position *k*) while other digits are zero. Thus, there are only two meaningful possibilities for each correspondent part: (1) $j_{d+s}=j_{s}=0$ in most cases, so $i_{s}=k_{s}$ is the only case with $c_{j_{s},j_{d+s}}^{i_{s},k_{s}}\neq 0$; or (2) one and only one position s=k or d+s=k in $J_{4}^{d}$ has $j_{k}=i$, either for $j_{s}$ or $j_{d+s}$, while the other is zero. This last case implies only two possibilities for $i_{s},k_{s}$: Case (A) one of $i_{s},k_{s}$ is *i* and other is zero (both possibilities are possible); or Case (B) $i,i_{s},k_{s}$ are different among them and from zero, thus they are a permutation $i,i^{\prime },i^{\prime \prime }$ of 1,2,3. In this case, there are two possibilities, $i_{s}=i^{\prime },k_{s}=i^{\prime \prime }$ or $i_{s}=i^{\prime \prime },k_{s}=i^{\prime }$.

**Figure 1:**
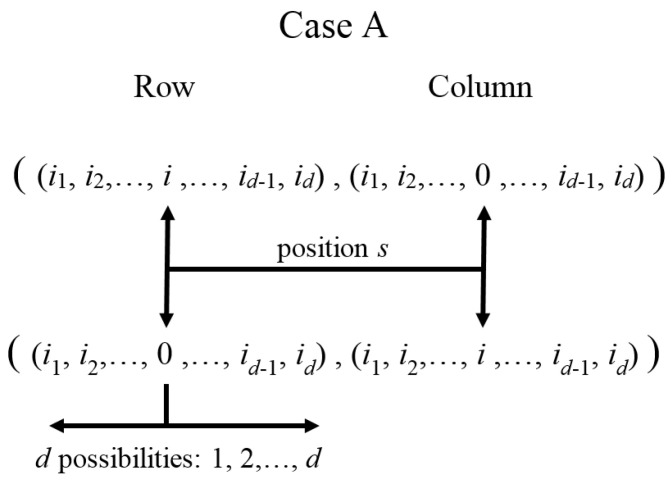
First case for a pair of entries in which $\langle \Psi_{I_{4}^{d}}\left|H_{l_{i}}\right|\Psi_{K_{4}^{d}}\rangle$ is non-zero. In them, for a fixed position s=k in the row and the column labels appears *i* or 0, while the other corresponding positions in the row and in the column have the same values.

Case A is depicted in Figure 1 for indexes I,K being considered in $\langle \Psi_{I_{4}^{d}}\left|H_{l_{i}}\right|\Psi_{K_{4}^{d}}\rangle$. There is a pair of entries whose labels for rows and columns have 0 or *i* in the position s=k: $\left(\left(i_{1},i_{2},\ldots ,i,\ldots ,i_{d}\right),\left(i_{1},i_{2},\ldots ,0,\ldots ,i_{d}\right)\right)$ and $\left(\left(i_{1},i_{2},\ldots ,0,\ldots ,i_{d}\right),\left(i_{1},i_{2},\ldots ,i,\ldots ,i_{d}\right)\right)$. This will be named the 0↔i association (or index exchange) rule.

**Figure 2:**
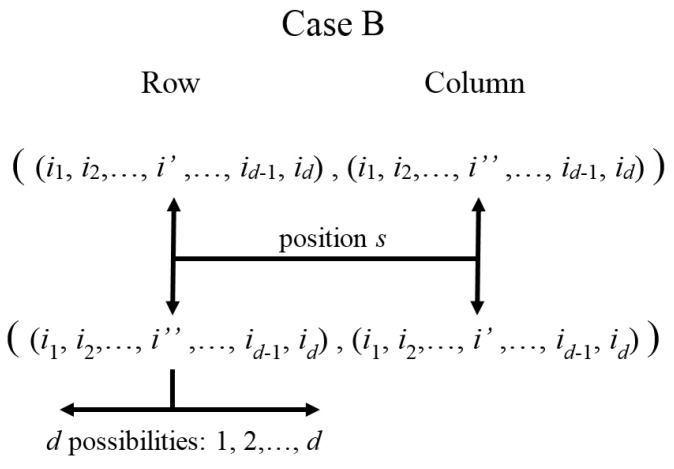
Second case for a pair of entries in which $\langle \Psi_{I_{4}^{d}}\left|H_{l_{i}}\right|\Psi_{K_{4}^{d}}\rangle$ is non-zero. In them, for a fixed position s=k in the row and the column labels appears $i^{\prime }$ or $i^{\prime \prime }$ alternatively ($i,i^{\prime },i^{\prime \prime }$ being a permutation of 1,2,3), while other corresponding positions in the row and in the column have the same values.

Case B is depicted in Figure 2. Here, there is a pair of entries whose labels for rows and columns have $i^{\prime }$ or $i^{\prime \prime }$ in the position s=k: $\left(\left(i_{1},i_{2},\ldots ,i^{\prime },\ldots ,i_{d}\right),\left(i_{1},i_{2},\ldots ,i^{\prime \prime },\ldots ,i_{d}\right)\right)$ and $\left(\left(i_{1},i_{2},\ldots ,i^{\prime \prime },\ldots ,i_{d}\right),\left(i_{1},i_{2},\ldots ,i^{\prime },\ldots ,i_{d}\right)\right)$, $i,i^{\prime },i^{\prime \prime }$ being a permutation of 1,2,3. This will be named the $i^{\prime }↔i^{\prime \prime }$ association (or index exchange) rule.

Clearly, in each case (A or B), for each pair of correspondent interaction terms with *i* and *k* fixed (k≤d and k+d positions), there are only two pairs on non-zero entries in rows $\left(i_{1},i_{2},\ldots ,i,\ldots ,i_{d}\right)$, $\left(i_{1},i_{2},\ldots ,0,\ldots ,i_{d}\right)$ for case A and in rows $\left(i_{1},i_{2},\ldots ,i^{\prime },\ldots ,i_{d}\right)$, $\left(\left(i_{1},i_{2},\ldots ,i^{\prime \prime },\ldots ,i_{d}\right)\right)$ for case B (with the corresponding column labels exchanged in both cases). Together with the diagonal entries generated by other adequate Hamiltonians (e.g., $H_{0}$ or $H_{{\mathrm{nl}}_{i}}$ as it will be seen), they will form 2×2 blocks. In fact, each non-zero entry for $H_{l_{i}}$ will have only two $h_{J}$ terms corresponding with $h_{0,0\ldots 0,i,0,\ldots ,0}$ with *i* in positions *s* or d+s (meaning local interaction with each element of the pair of correspondent parts in position *k*). Noting that labels in the position s=k in I (row) and K (column) for the non-zero entries are 0,i; i,0; $i^{\prime },i^{\prime \prime }$; or $i^{\prime \prime },i^{\prime }$, they cover all possibilities $i_{k}=0,1,2,3$. Thus, for a fixed column and defined i,k values in $\langle \Psi_{I_{4}^{d}}\left|H_{l_{i}}\right|\Psi_{K_{4}^{d}}\rangle$, there is exactly one non-zero row. Still, if two correspondent *k* elements are considered (local interactions on each element of a correspondent pair), they still generate only one non-zero row (each one with the two terms explained before).

Although there are 2d possibilities to select the position s=k in (22), they do not count as separate blocks because they appear in other entries in (18). Instead, each term in non-correspondent terms will appear in a different non-zero row, giving *d* non-zero rows as total. For each *i* direction of the interaction being included, additional non-zero rows will appear. This implies that 3d rows could appear when all parts have local interactions in the three spatial directions at time, destroying in this case the 2×2 block structure. Thus, maintaining local interactions in only one direction and on only one correspondent pair of elements, together, cases A and B form $\frac{1}{2}4^{d}$ 2×2 blocks as was required in the previous section. In any case, each non-zero entry will have the same 2 terms $h_{{\left(i4^{k-1}\right)}_{4}^{2d}}$ with different signs depending on $c_{j_{s},j_{d+s}}^{i_{s},k_{s}}$ involved in each factor of $H_{l_{i}}^{\left(k\right)}$. Clearly, blocks can be rearranged to adequately order the GBS basis elements getting the form (11). A brief analysis shows that there are no more diagonal-off elements in addition to last cases being generated by local terms. Additional diagonal-off elements come from the non-local terms, such as those in Table 1.

#### 4.3.3. Analysis of $\langle \Psi_{I_{4}^{d}}\left|H_{{\mathrm{nl}}_{i}}\right|\Psi_{K_{4}^{d}}\rangle$

With the correspondent parts definition and the analysis for $H_{l_{i}}$, we can identify two cases for the different terms $H_{{\mathrm{nl}}_{i}}^{\left(k,k^{\prime }\right)}$: (a) non-local interactions between correspondent parts; and (b) non-local interactions between non-correspondent parts. The discussion is similar to the previous subsection.

**Correspondent terms**$H_{{\mathrm{nl}}_{i}}^{\left(k,k+d\right)}$. This term in the Hamiltonian $H_{{\mathrm{nl}}_{i}}$ contains $\sigma_{0}⊗\ldots ⊗\sigma_{i}⊗\ldots ⊗\sigma_{i}⊗\ldots ⊗\sigma_{0}$ with $\sigma_{i}$ in positions *k* and k+d, and $\sigma_{0}$ in any other. When this term is allocated in $\langle \Psi_{I_{4}^{d}}\left|H_{{\mathrm{nl}}_{i}}\right|\Psi_{K_{4}^{d}}\rangle$ in agreement with (18), it does not cancel if each factor in the product become different from zero, implying $i_{s}=k_{s}\;\forall s=1,2,\ldots ,d$. Thus, this term gives non-zero entries only in the diagonal elements. Thus, each non-zero entry of $H_{{\mathrm{nl}}_{i}}$ will have *d* different terms in each diagonal element (one for each pair of interacting correspondent particles). Those terms will appear with different signs in each diagonal element in spite of $c_{j_{s},j_{d+s}}^{i_{s},k_{s}}$. At this point, note that results for $H_{{\mathrm{nl}}_{i}}^{\left(k,k+d\right)}$ and $H_{l_{i}}^{\left(k\right)}$ were expected due to the results in [11,26] and the separability of the GBS basis in their constitutive entangled pairs.

**Non-correspondent terms**$H_{{\mathrm{nl}}_{i}}^{\left(k,k^{\prime }\neq k+d\right)}$. These terms have a different behavior. Each term contains $\sigma_{0}⊗\ldots ⊗\sigma_{i}⊗\ldots ⊗\sigma_{i}⊗\ldots ⊗\sigma_{0}$, with $\sigma_{i}$ in positions *k* and $k^{\prime }$, and $\sigma_{0}$ in any other. It defines two pairs of correspondent parts involving $\sigma_{i}$: $\left[k,k+d,k^{\prime },k^{\prime }+d\right]$ if $k<k^{\prime }\leq d$ or $k,k+d,k^{\prime }-d,k^{\prime }$ if $k\leq d<k^{\prime }\leq 2d$. Then, each factor in (18) related with those two pairs ($s=k,k^{\prime }$ or $s=k,k^{\prime }-d$) will now include $\mathrm{Tr}(\sigma_{i_{s}}\sigma_{i}\sigma_{k_{s}})$ (until unitary factors), which is non-zero only if: (a) $i_{s}$ or $k_{s}$ are one of the pairs 0 and *i* or *i* and 0; (b) $i,i_{s},k_{s}$ are a permutation $i,i^{\prime },i^{\prime \prime }$ of 1,2,3 (having two cases depending on the parity). The last situation is similar to the local terms in the previous subsection, but in two parts simultaneously. The remaining factors for $s\neq k,k^{\prime }$ or $s\neq k,k^{\prime }-d$ will require $i_{s}=k_{s}$ in order to become non-zero. The latter scenario gives 16 possibilities for each term $h_{{\left(i\left(4^{k-1}+4^{k^{\prime }-1}\right)\right)}_{4}^{2d}}$, which will appear in diagonal-off positions obtained departing from the diagonal position $\left(i_{1},\ldots ,i_{d};i_{1},\ldots ,i_{d}\right)$ in $\langle \Psi_{I_{4}^{d}}\left|H_{{\mathrm{nl}}_{i}}\right|\Psi_{K_{4}^{d}}\rangle$, by changing each index in the pair $\left(i_{k},i_{k}^{\prime }\right)$ in the row, following the rules depicted in cases A and B. Thus, for each column and with $i,k,k^{\prime }$ fixed, only one row becomes non-zero, in agreement with the previous rule. Each entry of this kind involves four terms, including the four combinations of each pair of non-correspondent parts selected from the set $\left[k,k^{\prime },k+d,k^{\prime }+d\right]$. Instead, when all values *i* and $k,k^{\prime }$ are considered, a total of $3\cdot \frac{1}{2}d\left(d-1\right)$ non-zero rows appear in each column (clearly, by considering all these terms, SU(2) decomposition is not achieved).

#### 4.3.4. Analysis of $\langle \Psi_{I_{4}^{d}}\left|H_{{\mathrm{cnl}}_{i}}\right|\Psi_{K_{4}^{d}}\rangle$

**Correspondent terms**$H_{{\mathrm{cnl}}_{i}}^{\left(k,k+d\right)}$. For each term $H_{{\mathrm{cnl}}_{i}}^{\left(k,k+d\right)}$, the behavior is similar as for $H_{l_{i}}^{\left(k^{\prime }\right)}$. Because only one correspondent pair has $j_{p}=j_{s}\neq 0\neq j_{d+s}=k_{p}$ in (18), then $i_{s},k_{s}$ for $s=k^{\prime }$ should be 0,i or $j_{p},k_{p}$. For $s\neq k^{\prime }$, $i_{s}=k_{s}$. As before, it means that each term is diagonal-off by combining the values of index $k^{\prime }$ in I and K as before: 0,i; i,0; $j_{p},k_{p}$; and $k_{p},j_{p}$. For a fixed column and i,k, it will give four possibilities and two SU(2) blocks. Each entry will have two terms corresponding to the different parities *p*. Note that only one *i* and $k^{\prime }$ can be considered to achieve the SU(2) decomposition. Otherwise, for each column, 3d rows different from zero could appear, breaking the SU(2) decomposition as for the local interaction case.

**Non-correspondent terms**$H_{{\mathrm{cnl}}_{i}}^{\left(k,k^{\prime }\neq k+d\right)}$. As for $H_{{\mathrm{nl}}_{i}}^{\left(k,k^{\prime }\neq k+d\right)}$, in this case the only non-zero terms have $i_{s}=k_{s}$ for $s\neq k,k^{\prime },k-d,k^{\prime }-d$. Meanwhile, for the two remaining cases $s\in \left\{k,k^{\prime },k-d,k^{\prime }-d\right\}\cap \left\{1,2,\ldots ,d\right\}$, each $i_{s},k_{s}$ should be selected from the set $0,j_{p}$; $j_{p},0$; $i,k_{p}$; $k_{p},i$ or $0,k_{p}$; $k_{p},0$; $i,j_{p}$; $j_{p},i$. In a specific column and fixing *i*, it will give 16 possibilities and 8 blocks in SU(2), as for the $H_{{\mathrm{nl}}_{i}}^{\left(k,k^{\prime }\neq k+d\right)}$ case. Note that parity *p* should be fixed in this case because each one gives a different decomposition. Each entry will contain four terms for each parity *p* combining the four possible interaction terms. Again, if all options for *i* and $k,k^{\prime },p$ are considered, then 3·d(d−1) non-zero rows will appear for each column, breaking the SU(2) decomposition. These terms are not commonly introduced in models such as Heisenberg–Ising and those related. Instead, for magnetic systems they are the first-order approximation in the spin–orbit coupling, introducing antisymmetric exchange as in the Dzyaloshinskii–Moriya model: $H_{DM}=\vec{D}\cdot \left(\vec{\sigma_{1}}\times \vec{\sigma_{2}}\right)$. There, $\vec{D}$ is the Dzyaloshinskii–Moriya vector defining the orientation of coupling. Here, as only one term can be included in order to preserve the SU(2) reduction property, this coupling should be strictly oriented.

### 4.4. Explicit Analytical Formulas for Hamiltonian Components

After the last analysis, it is clear that other candidates to generate SU(2) decomposition are possible, but they involve more than two parts at a time (as in the case of $H_{0}$), which are non-physical for common point-like interactions. Nevertheless, these terms could appear for the quantum mechanical extended objects in which (1) is a mere expansion of the interactions. Therefore, we will restrict our remaining discussion to local or pairwise interactions. In this section, analytical formulas for $\langle \Psi_{I_{4}^{d}}\left|H_{l_{i}}\right|\Psi_{K_{4}^{d}}\rangle$, $\langle \Psi_{I_{4}^{d}}\left|H_{{\mathrm{nl}}_{i}}\right|\Psi_{K_{4}^{d}}\rangle$, and $\langle \Psi_{I_{4}^{d}}\left|H_{{\mathrm{cnl}}_{i}}\right|\Psi_{K_{4}^{d}}\rangle$ are provided to summarize the previous findings and because of their utility for optimal computer simulation purposes for larger systems. In order to simplify the expressions, we introduce the definition of the following generalized Kronecker delta:

(23) $$\begin{array}{c} \delta_{IK}^{S}\equiv \prod_{\begin{array}{c} s=1\\ s\notin S \end{array}}^{d}\delta_{i_{s}k_{s}}, \end{array}$$

where S is a set of scripts of the excluded parts in the product. Thus, for $H_{l_{i}}$:

(24) $$\begin{array}{cc} \langle \Psi_{I_{4}^{d}}\left|H_{l_{i}}\right|\Psi_{K_{4}^{d}}\rangle = & \sum_{k^{\prime }=1}^{d}\delta_{IK}^{\left\{k^{\prime }\right\}}{H_{l_{i}}^{k^{\prime }}}_{I_{4}^{d},K_{4}^{d}},\\ \mathrm{with}:\;{H_{l_{i}}^{k^{\prime }}}_{I_{4}^{d},K_{4}^{d}}= & \sum_{t^{\prime }=0}^{1}h_{{\left(i4^{k^{\prime }+dt^{\prime }-1}\right)}_{4}^{2d}}F_{i,k^{\prime }}^{i\delta_{0,t^{\prime }},i\delta_{1,t^{\prime }}}, \end{array}$$

by noting $c_{0,0}^{i_{s},i_{s}}=1$. In ${H_{l_{i}}^{k^{\prime }}}_{I_{4}^{d},K_{4}^{d}}$, $\left[k^{\prime },k^{\prime }+d\right]$ is the correspondent pair where each local interaction is being applied. There, the exchange factor generating the diagonal-off entries in the SU(2) blocks is:

(25) $$\begin{array}{ccc} F_{i,k^{\prime }}^{j,k} & = & \delta_{i_{k^{\prime }}0}\delta_{k_{k^{\prime }}i}c_{j,k}^{0,i}+\delta_{i_{k^{\prime }}i}\delta_{k_{k^{\prime }}0}c_{j,k}^{i,0}+\sum_{i^{\prime },i^{\prime \prime }=1}^{3}\epsilon_{ii^{\prime }i^{\prime \prime }}^{2}\delta_{i_{k^{\prime }}i^{\prime }}\delta_{k_{k^{\prime }}i^{\prime \prime }}c_{j,k}^{i^{\prime },i^{\prime \prime }}. \end{array}$$

For $H_{{\mathrm{nl}}_{i}}$:

(26) $$\begin{array}{cc} \langle \Psi_{I_{4}^{d}}\left|H_{{\mathrm{nl}}_{i}}\right|\Psi_{K_{4}^{d}}\rangle = & \sum_{k^{\prime }=1}^{d}\delta_{IK}^{\left\{k^{\prime }\right\}}{H_{{\mathrm{nl}}_{i}}^{c,k^{\prime }}}_{I_{4}^{d},K_{4}^{d}}+\sum_{k^{\prime \prime }>k^{\prime }=1}^{d}\delta_{IK}^{\left\{k^{\prime },k^{\prime \prime }\right\}}{H_{{\mathrm{nl}}_{i}}^{nc,k^{\prime }k^{\prime \prime }}}_{I_{4}^{d},K_{4}^{d}},\\ \mathrm{with}:\;{H_{{\mathrm{nl}}_{i}}^{c,k^{\prime }}}_{I_{4}^{d},K_{4}^{d}}= & \;h_{{\left(i\left(4^{k^{\prime }-1}+4^{k^{\prime }+d-1}\right)\right)}_{4}^{2d}}\delta_{i_{k^{\prime }}k_{k^{\prime }}}c_{i,i}^{i_{k^{\prime }},i_{k^{\prime }}},\\ \;{H_{{\mathrm{nl}}_{i}}^{nc,k^{\prime }k^{\prime \prime }}}_{I_{4}^{d},K_{4}^{d}}= & \sum_{t^{\prime },t^{\prime \prime }=0}^{1}h_{{\left(i\left(4^{k^{\prime }+dt^{\prime }-1}+4^{k^{\prime \prime }+dt^{\prime \prime }-1}\right)\right)}_{4}^{2d}}F_{i,k^{\prime }}^{i\delta_{0,t^{\prime }},i\delta_{1,t^{\prime }}}F_{i,k^{\prime \prime }}^{i\delta_{0,t^{\prime \prime }},i\delta_{1,t^{\prime \prime }}}. \end{array}$$

Each term belongs to correspondent and non-correspondent interactions, respectively. In ${H_{{\mathrm{nl}}_{i}}^{c,k^{\prime }}}_{I_{4}^{d},K_{4}^{d}}$ and ${H_{{\mathrm{nl}}_{i}}^{nc,k^{\prime }k^{\prime \prime }}}_{I_{4}^{d},K_{4}^{d}}$, $\left[k^{\prime },k^{\prime \prime }\right]$ are the parts with non-local interactions between them. Similarly, for $H_{{\mathrm{cnl}}_{i}}$:

(27) $$\begin{array}{cc} \langle \Psi_{I_{4}^{d}}\left|H_{{\mathrm{cnl}}_{i}}\right|\Psi_{K_{4}^{d}}\rangle = & \sum_{k^{\prime }=1}^{d}\delta_{IK}^{\left\{k^{\prime }\right\}}{H_{{\mathrm{cnl}}_{i}}^{c,k^{\prime }}}_{I_{4}^{d},K_{4}^{d}}+\sum_{p=0}^{1}\sum_{k^{\prime \prime }>k^{\prime }=1}^{d}\delta_{IK}^{\left\{k^{\prime },k^{\prime \prime }\right\}}{H_{{\mathrm{cnl}}_{i}}^{nc,k^{\prime }k^{\prime \prime }p}}_{I_{4}^{d},K_{4}^{d}},\\ \mathrm{with}:\;{H_{{\mathrm{cnl}}_{i}}^{c,k^{\prime }}}_{I_{4}^{d},K_{4}^{d}}= & \;\sum_{p=0}^{1}h_{{\left(j_{p}4^{k^{\prime }-1}+k_{p}4^{k^{\prime }+d-1}\right)}_{4}^{2d}}F_{i,k^{\prime }}^{j_{p},k_{p}},\\ \;{H_{{\mathrm{cnl}}_{i}}^{nc,k^{\prime }k^{\prime \prime }p}}_{I_{4}^{d},K_{4}^{d}}= & \sum_{t^{\prime },t^{\prime \prime }=0}^{1}h_{{\left(j_{p}4^{k^{\prime }+dt^{\prime }-1}+k_{p}4^{k^{\prime \prime }+dt^{\prime \prime }-1}\right)}_{4}^{2d}}F_{j_{p},k^{\prime }}^{j_{p}\delta_{0,t^{\prime }},j_{p}\delta_{1,t^{\prime }}}F_{k_{p},k^{\prime \prime }}^{k_{p}\delta_{0,t^{\prime \prime }},k_{p}\delta_{1,t^{\prime \prime }}}. \end{array}$$

Again, $H_{{\mathrm{cnl}}_{i}}^{c,k^{\prime }}$ and $H_{{\mathrm{cnl}}_{i}}^{nc,k^{\prime }k^{\prime \prime }p}$ are the correspondent and non-correspondent interactions in the Hamiltonian, $\left[k^{\prime },k^{\prime \prime }\right]$ being the parts where there are non-local interactions. This explicitly shows the existence of four (for $H_{l_{i}}^{k^{\prime }}$ and $H_{{\mathrm{cnl}}_{i}}^{c,k^{\prime }}$) and sixteen (for $H_{{\mathrm{nl}}_{i}}^{nc,k^{\prime }k^{\prime \prime }}$ and $H_{{\mathrm{cnl}}_{i}}^{nc,k^{\prime }k^{\prime \prime }p}$) diagonal-off entries, respectively, in agreement with cases A and B depicted by Figure 1 and Figure 2 (if only single specific values of $i,k^{\prime },k^{\prime \prime }$ are considered instead of the whole sum), generating $2\times 4^{d-1}=\frac{1}{2}\times 4^{d}$ and $8\times 4^{d-2}=\frac{1}{2}\times 4^{d}$ blocks, respectively. Then, the SU(2) decomposition could be achieved only by: (a) including any desired non-local terms $H_{{\mathrm{nl}}_{i}}^{c,k^{\prime }}$ (to generate the diagonal elements); and (b) including only one type of interaction among $H_{l_{i}}^{k^{\prime }}$, $H_{{\mathrm{nl}}_{i}}^{nc,k^{\prime }k^{\prime \prime }}$, $H_{{\mathrm{cnl}}_{i}}^{c,k^{\prime }}$ or $H_{{\mathrm{cnl}}_{i}}^{nc,k^{\prime }k^{\prime \prime }p}$ for concrete values for $i,k^{\prime },k^{\prime \prime }$, and *p*.

An important property used later for $F_{i,k^{\prime }}^{j_{s},j_{d+s}}$ is that only one term in (25) remains with the election of $i_{k^{\prime }}$ and $k_{k^{\prime }}$. Because each $c_{j,k}^{j_{s},j_{d+s}}$ is real or imaginary, and more concretely as a brief analysis shows, if it is not zero, then it becomes imaginary only if $j_{s}$ or $j_{d+s}$ is equal to 2, this property is transferred to $F_{i,k^{\prime }}^{j_{s},j_{d+s}}$.

## 5. Specific Interactions Generating SU(2) Decomposition

In this section, we summarize and organize the global findings to reach the SU(2) block structure on the GBS basis. Finally, we conclude that there are three great types of interactions that are able to generate the block structure depicted in Section 3.

### 5.1. General Depiction of Interactions Having SU(2) Decomposition for the GBS Basis

Based on the previous discussion, there are three groups of interactions that are able to generate the SU(2) decomposition on the GBS basis. The first one (Type I) involves all kinds of non-local and non-crossed interactions between any two correspondent parts in any direction. These terms generate the diagonal terms depicted previously in the Hamiltonian. Together, only two local interactions in only one specific direction and on only one pair of correspondent parts, $k_{l}$, should be included to generate the diagonal-off entries. Thus, this group of interactions generates the SU(2) blocks. Note that local interaction terms could be intended as external driven fields as in [11,26]. The second interaction (Type II) is obtained by substituting the previous local interactions with non-local interactions among only those non-correspondent elements included in two pairs of correspondent parts. This means that if $k,k^{\prime },k+d,k^{\prime }+d$ with $k<k^{\prime }\leq \;d$ are these elements in the two correspondent parts, then only the interactions between the following non-correspondent elements are allowed: $\left[k,k^{\prime }\right],\left[k,k^{\prime }+d\right],\left[k^{\prime },k+d\right]$, and $\left[k+d,k^{\prime }+d\right]$. This group of four interactions generates the diagonal-off terms to conform the SU(2) blocks. Nevertheless, the Type II interaction should normally be understood as a non-driven process of control. Note that Type II interaction could be classified into two other subclasses: (a) Type IIa for non-crossed interactions $H_{{\mathrm{nl}}_{i}}^{nc,k^{\prime }k^{\prime \prime }}$; and (b) Type IIb for crossed interactions $H_{{\mathrm{cnl}}_{i}}^{nc,k^{\prime }k^{\prime \prime }p}$. Finally, the third interaction (Type III) involves both the non-local and non-crossed interactions, with the inclusion of crossed interactions between one specific correspondent pair.

**Figure 3:**
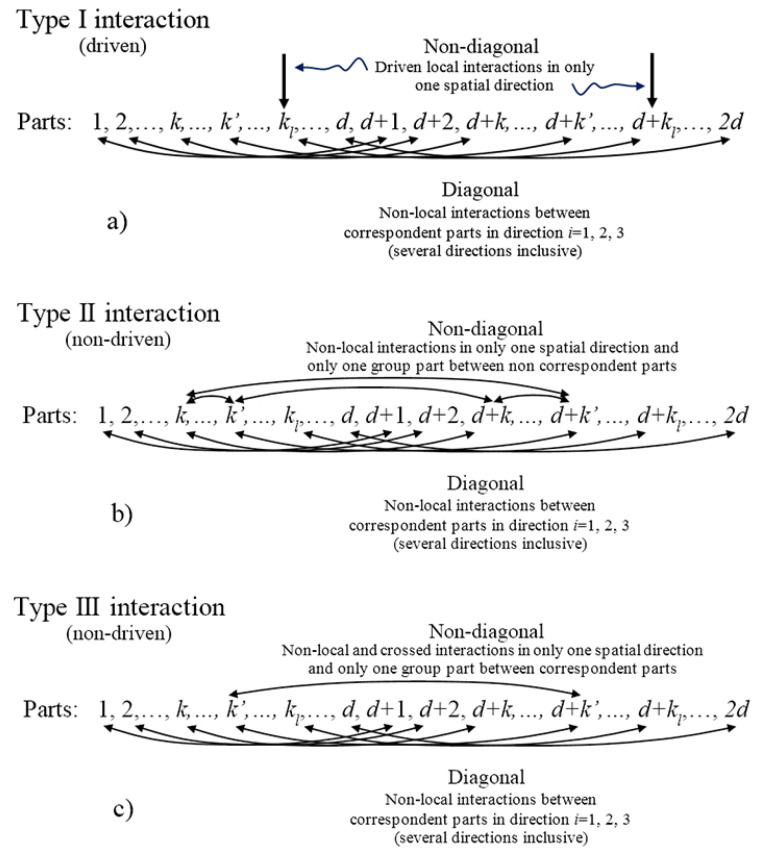
Three types of physical interactions able to generate the block decomposition. Non-local and non-crossed interactions among any correspondent parts combined with: (**a**) local interactions on only two correspondent parts ($k_{l},k_{l}+d$); (**b**) any two non-correspondent parts in only two specific pairs of correspondent parts of only one subtype, non-crossed or crossed; and (**c**) crossed interactions between a specific pair of correspondent parts.

In order to clarify the structure of those notable interaction architectures as special cases of Hamiltonian (1), we make some remarks as follows. Figure 3 summarizes the three types of interactions depicted above by listing the 2d qubits involved and then relating them with arrows in agreement with their mutual interactions. Then:

A: Curved arrows point out those qubits related through entangling operations in any case.
B: All curved arrows in the bottom refer to Heisenberg–Ising-like (non-crossed) interactions involving the three possible spatial directions together. Those interaction relations set the correspondent pairs.
C: For the curved arrows in the top, two kinds of entangling operations can be considered according to the text: Heisenberg–Ising-like (non-crossed) interactions or Dzyaloshinskii–Moriya-like (crossed) interactions. Only one characteristic spatial direction is allowed.
D: Type II interactions can be split into Type IIa and Type IIb if interactions in the top are non-crossed or crossed (between parts of two different correspondent pairs), respectively. Type IIb interactions in the top admits only one possible parity from the two possible.
E: Type III interactions admit only crossed interactions in the top between parts of one specific correspondent pair, but the two possible parities together are allowed.
F: For Figure 3a, the right arrows correspond to external local interactions such as those generated by magnetic fields on spin-based qubits. Due to their locality, they are referred to as driven interactions, although it actually depends on the available control of the interactions.

**Figure 4:**
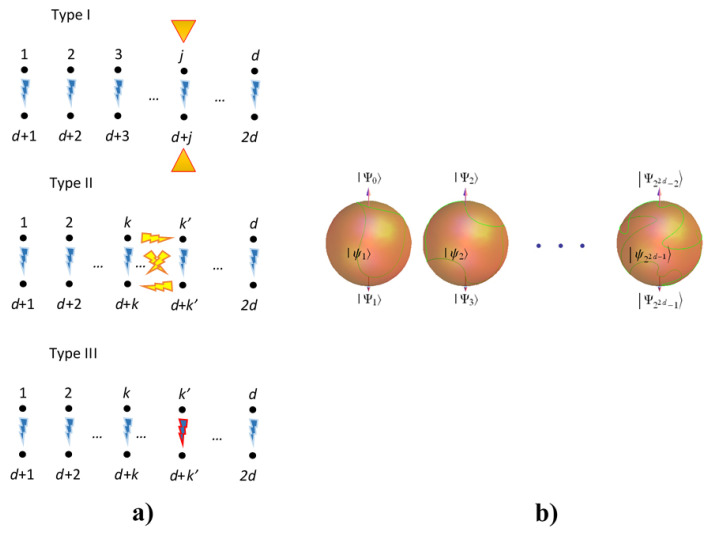
Representation of qubit interactions able to generate SU(2) decomposition: (**a**) Type I, II, and III interactions among 2d qubits (Type III assumes the inclusion of crossed interactions in the pair $k^{\prime }$); and (**b**) Distributed evolution on $2^{2d-1}$ Bloch spheres, each one for the states $|\psi_{j}\rangle$.

Figure 4a shows a pictorial representation of each interaction, where the pairing is graphically represented. Therein, yellow rays with blue contour are non-crossed interactions in the three spatial directions [B]. Yellow rays with red contour represent one interaction from non-crossed or crossed entangling interactions in only one spatial direction [D]. Blue rays with red contour indicate non-crossed interaction in three spatial directions together with a crossed interaction in only one direction [E]. Yellow triangles indicate local interactions on the respective qubits in only one correspondent pair [F].

In particular, note that this description is in agreement with the results in Table 1 for d=1, although it is a special case because diagonal-off entries for Type I, II, and III coincide in the same diagonal-off entries, so both interactions could be combined at the same time, preserving the SU(2) decomposition. This case has a richer structure for control in terms of the number of free parameters involved with respect to the number of parts to be controlled. Note that while Types I and III are only able to modify the inner entanglement of the correspondent pairs, Type II interaction (Type IIa and IIb) allows the modification of the global entanglement between different correspondent pairs, thus letting it spread on the entire system by switching the pairs involving interactions generating diagonal-off entries.

### 5.2. General Structure of SU(2) Blocks

A complementary analysis of SU(2) blocks obtained for the last interactions is given in this subsection. Their form is particularly useful as a connection with optimal control schemes, such as those presented in [9]. In any case (Type I, II, or III), each block ${S_{H}}_{I,I^{\prime }}$ (with $I,I^{\prime }$ the rows in which is situated) has the form:

(28) SHI,I′=h11h12h12*h22=h11+h222II,I′+Re(h12)XI,I′−Im(h12)YI,I′+h11−h222ZI,I′,

where $\left\{I_{I,I^{\prime }},X_{I,I^{\prime }},Y_{I,I^{\prime }},Z_{I,I^{\prime }}\right\}$ is the Pauli basis for the SU(2) block. If the Hamiltonian coefficients involved in the block are time-independent, then the corresponding ${S_{U}}_{I,I^{\prime }}$ block in the evolution matrix becomes:

(29) SUI,I′=eiSHI,I′tℏ=eih11+h222ℏteiωn·sI,I′t=eih11+h222ℏt(cosωt+isinωtn·sI,I′)=eih11+h222ℏtcosωt+ih11−h222ℏωsinωtih12ℏωsinωtih12*ℏωsinωtcosωt−ih11−h222ℏωsinωt,with:n=1ℏω(Re(h12),−Im(h12),h11−h222),sI,I′=(XI,I′,YI,I′,ZI,I′),ℏω=|h12|2+14|h11−h22|2,

clearly belonging to U(1)×SU(2) (see Appendix A.2). As stated previously, $F_{j,k^{\prime }}^{j_{s},j_{d+s}}$ is imaginary only if $j_{s}$ or $j_{d+s}$ is 2. Thus, only one component from $n_{1}$ or $n_{2}$ is different from zero because non-diagonal entries of block in (24), (26), and (27) are always real or imaginary. This reduces the optimal control to the second case reported by [9]. An additional analysis shows that $h_{11}\pm h_{22}\neq 0$ in general (without imposing restrictions on the non-local strengths $h_{J}$). This aspect will be relevant later.

### 5.3. Structure of SU(2) Blocks for Each Interaction

Several classical interactions fitting in the current procedure were analyzed. All them generate blocks (not necessarily SU(2) blocks) when they are expressed in the GBS basis, denoting a kind of universality for this basis due to its ability to gather similar interactions through simplified representations. For the sake of the search for SU(2) decomposition, we discuss finally closed forms for the specific Hamiltonians able to achieve the SU(2) decomposition. These formulas are quite useful for computer simulation purposes.

#### 5.3.1. Blocks in Type I Interaction

This interaction includes non-crossed spin interactions between correspondent particles in all spatial directions and external local interactions on the pair $\left[k^{\prime },k^{\prime }+d\right]$ of correspondent particles in direction *j*. From (24)–(26), it can be written as:

(30) $$\begin{array}{cc} H_{I}= & H_{D}+H_{ND_{I}}^{\left(j,k^{\prime }\right)},\\ \mathrm{with}: & H_{D}\equiv \sum_{i^{\prime }=1}^{3}\sum_{k=1}^{d}h_{{\left(i^{\prime }\left(4^{k-1}+4^{k+d-1}\right)\right)}_{4}^{2d}}\overset{2d}{\underset{s=1}{⨂}}\sigma_{{\left(i^{\prime }\left(4^{k-1}+4^{k+d-1}\right)\right)}_{4,s}^{2d}},\\ H_{ND_{I}}^{\left(j,k^{\prime }\right)} & =\sum_{t^{\prime }=0}^{1}h_{{\left(j4^{k^{\prime }+dt^{\prime }-1}\right)}_{4}^{2d}}\overset{2d}{\underset{s=1}{⨂}}\sigma_{{\left(j4^{k^{\prime }+dt^{\prime }-1}\right)}_{4,s}^{2d}}, \end{array}$$

generating SU(2) blocks with the diagonal terms from non-local interactions between correspondent parts and the non-diagonal terms from local interactions. Departing from (24)–(26), we obtain for the Hamiltonian components:

(31) $$\begin{array}{cc} \langle \Psi_{I_{4}^{d}}\left|H_{I}\right|\Psi_{K_{4}^{d}}\rangle = & \;\delta_{IK}\sum_{i^{\prime }=1}^{3}\sum_{k^{\prime \prime }=1}^{d}\left({\left(-1\right)}^{\delta_{i^{\prime },2}+\left(1-\delta_{i^{\prime },i_{k^{\prime \prime }}}\right)\left(1-\delta_{0,i_{k^{\prime \prime }}}\right)}h_{{\left(i^{\prime }\left(4^{k^{\prime \prime }-1}+4^{k^{\prime \prime }+d-1}\right)\right)}_{4}^{2d}}\right)+\\ & \sum_{t^{\prime }=0}^{1}h_{{\left(j4^{k^{\prime }+dt^{\prime }-1}\right)}_{4}^{2d}}\delta_{IK}^{\left\{k^{\prime }\right\}}F_{j,k^{\prime }}^{j\delta_{0,t^{\prime }},j\delta_{1,t^{\prime }}}\equiv {H_{D}}_{IK}+{H_{ND_{I}}^{\left(j,k^{\prime }\right)}}_{IK}. \end{array}$$

The last formula is obtained noting that $c_{i,i}^{i_{k^{\prime \prime }},i_{k^{\prime \prime }}}={\left(-1\right)}^{\delta_{i,2}+\left(1-\delta_{i,i_{k^{\prime \prime }}}\right)\left(1-\delta_{0,i_{k^{\prime \prime }}}\right)}$. The first term of the last expressions denotes the diagonal terms of interaction. This formula shows that the pair of entries in the diagonal of each SU(2) block are generally different. Because the block is formed by switching an index $i_{k^{\prime \prime }}$ in the row labels (or two as in the following cases) in agreement with the association rules 0↔j or i↔k (*j* is the direction associated to the interaction and i,j,k a permutation of 1,2,3), then for $i^{\prime }\neq j$ the terms in ${H_{D}}_{IK}$ have a sign change. This implies that in general $h_{11}\neq h_{22}$ in (28), generating non-diagonal ${S_{H}}_{I,I^{\prime }}$-blocks. The second term contains the four diagonal-off elements generating two blocks with two terms each. Note that Hamiltonian terms ($h_{I}$) are real together with $c_{i,i}^{i_{k^{\prime \prime }},i_{k^{\prime \prime }}}$, so diagonal terms are real, as expected. Diagonal-off terms will be real or imaginary depending on $F_{j,k^{\prime }}^{j,0},F_{j,k^{\prime }}^{0,j}$. In any case, concretely, they are imaginary only if j=2.

Note that this interaction (when it is applied to a combination of correspondent pairs with bipartite entangled states) generates only non-local operations on each correspondent pair, such as those presented in [11,13]. Still switching the direction *j* and the correspondent pair $k^{\prime }$ on which the local interaction is applied, this kind of Hamiltonian cannot generate extended entanglement between correspondent pairs more than that included in the initial state. This means that if the initial state is separable by correspondent pairs, it will remain separable at this level (but should be able to entangle or untangle the parts of each pair). Conversely, it cannot disentangle each correspondent pair from the remaining state in more complex cases. We dedicate a later section to analyzing these topics.

#### 5.3.2. Blocks in Type II Interaction

**Type IIa:** In this case, the interaction is completely non-local between correspondent pairs to generate the diagonal entries, and in only one direction between non-correspondent parts in two correspondent pairs to generate the diagonal-off entries. The Hamiltonian becomes:

(32) $$\begin{array}{cc} H_{IIa}= & H_{D}+H_{ND_{IIa}}^{\left(j,k^{\prime }k^{\prime \prime }\right)},\\ \mathrm{with}: & H_{ND_{IIa}}^{\left(j,k^{\prime }k^{\prime \prime }\right)}=\sum_{t^{\prime },t^{\prime \prime }=0}^{1}h_{{\left(j\left(4^{k^{\prime }+dt^{\prime }-1}+4^{k^{\prime \prime }+dt^{\prime \prime }-1}\right)\right)}_{4}^{2d}}\overset{2d}{\underset{s=1}{⨂}}\sigma_{{\left(j\left(4^{k^{\prime }+dt^{\prime }-1}+4^{k^{\prime \prime }+dt^{\prime \prime }-1}\right)\right)}_{4,s}^{2d}}, \end{array}$$

with a non-local and non-crossed interaction in the direction *j* for the group of non-correspondent terms defined by $k^{\prime }<k^{\prime \prime }\leq d$. The Hamiltonian entries are similar to those in (24)–(26), but with the last restriction for the non-correspondent terms of interaction. Due to discussion in the previous subsection, diagonal-off entries in the Hamiltonian are now always real. The components become:

(33) $$\begin{array}{cc} \langle \Psi_{I_{4}^{d}}\left|H_{IIa}\right|\Psi_{K_{4}^{d}}\rangle = & \;{H_{D}}_{IK}+{H_{ND_{IIa}}^{\left(j,k^{\prime }k^{\prime \prime }\right)}}_{IK},\\ {H_{ND_{IIa}}^{\left(j,k^{\prime }k^{\prime \prime }\right)}}_{IK}\equiv & \sum_{t^{\prime },t^{\prime \prime }=0}^{1}h_{{\left(j\left(4^{k^{\prime }+dt^{\prime }-1}+4^{k^{\prime \prime }+dt^{\prime \prime }-1}\right)\right)}_{4}^{2d}}\delta_{IK}^{\left\{k^{\prime },k^{\prime \prime }\right\}}F_{j,k^{\prime }}^{j\delta_{0,t^{\prime }},j\delta_{1,t^{\prime }}}F_{j,k^{\prime \prime }}^{j\delta_{0,t^{\prime \prime }},j\delta_{1,t^{\prime \prime }}}. \end{array}$$

**Type IIb:** For this interaction, the non-diagonal part generated by the non-local interaction between non-correspondent parts is supplied by a non-local and crossed interaction among non-correspondent parts of two correspondent pairs:

(34) $$\begin{array}{cc} H_{IIb}= & H_{D}+H_{ND_{IIb}}^{\left(i,k^{\prime }k^{\prime \prime }p\right)},\\ \mathrm{with}: & H_{ND_{IIb}}^{\left(i,k^{\prime }k^{\prime \prime }p\right)}\equiv \sum_{t^{\prime },t^{\prime \prime }=0}^{1}h_{{\left(j_{p}4^{k^{\prime }+dt^{\prime }-1}+k_{p}4^{k^{\prime \prime }+dt^{\prime \prime }-1}\right)}_{4}^{2d}}\overset{2d}{\underset{s=1}{⨂}}\sigma_{{\left(j_{p}4^{k^{\prime }+dt^{\prime }-1}+k_{p}4^{k^{\prime \prime }+dt^{\prime \prime }-1}\right)}_{4,s}^{2d}}. \end{array}$$

As before, $i,j_{p},k_{p}$ is a permutation of 1,2,3 with parity p=0,1 (even and odd, respectively). Thus, the components become:

(35) $$\begin{array}{cc} \langle \Psi_{I_{4}^{d}}\left|H_{IIb}\right|\Psi_{K_{4}^{d}}\rangle = & \;{H_{D}}_{IK}+{H_{ND_{IIb}}^{\left(i,k^{\prime }k^{\prime \prime }p\right)}}_{IK},\\ {H_{ND_{IIb}}^{\left(i,k^{\prime }k^{\prime \prime }p\right)}}_{IK}= & \;\sum_{t^{\prime },t^{\prime \prime }=0}^{1}h_{{\left(j_{p}4^{k^{\prime }+dt^{\prime }-1}+k_{p}4^{k^{\prime \prime }+dt^{\prime \prime }-1}\right)}_{4}^{2d}}\delta_{IK}^{\left\{k^{\prime },k^{\prime \prime }\right\}}F_{j_{p},k^{\prime }}^{j_{p}\delta_{0,t^{\prime }},j_{p}\delta_{1,t^{\prime }}}F_{k_{p},k^{\prime \prime }}^{k_{p}\delta_{0,t^{\prime \prime }},k_{p}\delta_{1,t^{\prime \prime }}}. \end{array}$$

The non-diagonal entries are now imaginary, except for i=2.

#### 5.3.3. Blocks in Type III Interaction

Finally, for Type III interaction, the non-diagonal part is generated by the non-local and crossed interaction between a pair of correspondent parts $k^{\prime }$:

(36) $$\begin{array}{cc} H_{III}= & \;H_{D}+H_{ND_{III}}^{\left(i,k^{\prime }\right)},\\ \mathrm{with}: & H_{ND_{III}}^{\left(i,k^{\prime }\right)}=\sum_{p=0}^{1}h_{{\left(j_{p}4^{k^{\prime }-1}+k_{p}4^{k^{\prime }+d-1}\right)}_{4}^{2d}}\overset{2d}{\underset{s=1}{⨂}}\sigma_{{\left(j_{p}4^{k^{\prime }-1}+k_{p}4^{k^{\prime }+d-1}\right)}_{4,s}^{2d}}, \end{array}$$

with the Hamiltonian components:

(37) $$\begin{array}{cc} \langle \Psi_{I_{4}^{d}}\left|H_{III}\right|\Psi_{K_{4}^{d}}\rangle = & \;{H_{D}}_{IK}+{H_{ND_{III}}^{\left(i,k^{\prime }\right)}}_{IK},\\ {H_{ND_{III}}^{\left(i,k^{\prime }\right)}}_{IK}= & \;\sum_{p=0}^{1}h_{{\left(j_{p}4^{k^{\prime }-1}+k_{p}4^{k^{\prime }+d-1}\right)}_{4}^{2d}}\delta_{IK}^{\left\{k^{\prime }\right\}}F_{i,k^{\prime }}^{j_{p},k_{p}}, \end{array}$$

where non-diagonal entries are imaginary only if i=2.

Figure 4b shows a distributed evolution on $2^{2d-1}$ Bloch spheres for the states $|\psi_{j}\;\rangle =\;\alpha_{2j-2}|\Psi_{2j-2}\rangle +\alpha_{2j-1}|\Psi_{2j-1}\rangle$ , which are part of the global state $|\psi \rangle =\sum_{j=1}^{2^{2d-1}}|\psi_{j}\rangle$, where each $|\Psi_{k}\rangle$ is an element of the GBS basis. Each state $|\psi_{j}\rangle$ evolves as a different curve on each Bloch sphere depending on parameters $h_{J}$.

Finally, we should note that each of the previous interactions involves labels to be completely identified, namely: $H_{I}^{\left(j,k^{\prime }\right)},H_{IIa}^{\left(j,k^{\prime },k^{\prime \prime }\right)},H_{IIb}^{\left(j,k^{\prime },k^{\prime \prime },p\right)}$, and $H_{III}^{\left(j,k^{\prime },k^{\prime \prime }\right)}$. These labels will be omitted by simplicity unless their specification becomes needed. In any case, closed expressions (31), (33), (35), and (37) are computationally efficient to generate matrix representations of Hamiltonians $H_{I},H_{IIa,b},H_{III}$, and for their respective *U*, inclusively in the time-dependent case, although a numerical approach to construct could also be necessary.

### 5.4. Available Parameters and Structure of Entries

The number of free parameters (coefficients $h_{I}$ of Hamiltonian) and their availability are important to set control procedures. In this section, we count the entries and terms for each Hamiltonian, summarizing the previous findings. If D≤3 is the number of spatial dimensions involved in each interaction, then the accounting of free parameters generating the SU(2) decomposition, together with the maximum number of entries by column able to generate it (breaking the SU(2) decomposition) is reported in Table 2. Note that the number of entries by column for all Hamiltonians (labeled with *i*, in some sense the direction of the interaction) can be increased by a factor *D* if all directions are considered at time. In the table, each Hamiltonian analyzed is reported, arriving at the main Hamiltonians $H_{I},H_{IIa,b}$, and $H_{III}$. Accounting shows few free parameters at time (compared with the exponential growth of the matrix with the system size *d*) to set a whole control (over all blocks) in one period of constant driven parameters, suggesting the use of time-dependent or at least constant-piecewise parameters to increase the control.

#### 5.4.1. Structure of Diagonal Entries Belonging to a Specific Block

Other aspects should be discussed. The first is related to terms in diagonal entries generated by non-local interactions $H_{{\mathrm{nl}}_{j}}^{c,s}$ among correspondent parts. Note that blocks are generated by interactions other than those, which are prescribed as a difference in one ($H_{{\mathrm{nl}}_{i}}^{c,k^{\prime }}$ or $H_{{\mathrm{cnl}}_{i}}^{c,k^{\prime }}$) or two ($H_{{\mathrm{nl}}_{i}}^{nc,k^{\prime }k^{\prime \prime }}$ or $H_{{\mathrm{cnl}}_{i}}^{nc,k^{\prime }k^{\prime \prime }p}$) terms in the scripts labels, in agreement with the rules depicted in Figure 1 and Figure 2. This implies that there will be two or eight blocks, each one relating rows (and columns) differing in only one or two terms of their scripts, respectively. Note the diagonal entries for $H_{{\mathrm{nl}}_{j}}^{c,s}$ in (18) for the GBS defined as in [22]: $\mathrm{Tr}\left({\tilde{\sigma }}_{i_{s}}^{*}\sigma_{j}{\tilde{\sigma }}_{i_{s}}^{T}\sigma_{j}^{T}\right)=2{\left(-1\right)}^{\delta_{j,2}+\left(1-\delta_{j,i_{s}}\right)\left(1-\delta_{0,i_{s}}\right)}$. Then, for each three strengths for a fixed correspondent pair, there will be only four sign combinations (none is the negative of another) depending on: (a) the direction of the interaction involved (on the correspondent pair *s*) is j=2 or j≠2; and (b) $i_{s}$ for the $s^{\mathrm{th}}$ script is in the set {0,j} or in {i,k} (with i,j,k a permutation of 1,2,3). There, the factors corresponding to other correspondent pairs will be equal to one. Then, for the 3d terms included in all diagonal entries there will be $4^{d}$ combinations for the whole terms—precisely the number of rows. This implies that all diagonal entries are different (but not independent because there are only 3d parameters). For two rows differing in only one or two terms in their scripts, only the three or six terms corresponding with the strengths of $H_{{\mathrm{nl}}_{j}}^{c,s}$ for such correspondent pairs (associated with those terms in the scripts) will change their signs in the diagonal terms in their block. Consequently, for such $4^{d-1}$ or $4^{d-2}$ groups of blocks having the same scripts exchanged and generated by the whole combinations in the other d−1 or d−2 terms in their scripts, they will have the same $h_{11}-h_{22}$ parameters, respectively. Thus, it will be only two or eight different $h_{11}-h_{22}$ parameters for the entire *H*. Meanwhile, $h_{11}+h_{22}$ parameters could be different.

#### 5.4.2. Structure of Diagonal-Off Entries Belonging to a Specific Block

The second aspect is related to the explicit calculation of $c_{j_{s},j_{d+s}}^{i_{s},k_{s}}$ for the basic cases of interest in the diagonal-off entries. (a) For $H_{I}$ and $H_{IIa,b}$: $j_{s}=j,j_{d+s}=0$, or $j_{s}=0,j_{d+s}=j$ (*j* being the direction label involved in the local and non-local interactions between non-correspondent parts); and (b) for $H_{III}$: $j_{s}=j_{p},j_{d+s}=k_{p}$. Table 3 explicitly shows these values. Note the parallelism between their two halves (vertically and horizontally).

These cases generate the diagonal-off entries in each block in agreement with the exchange rules depicted previously for the $s^{\mathrm{th}}$ scripts of such entries’ rows: $\left(i_{s},k_{s}\right)\in \left\{\left(0,j\right),\left(j,0\right);\left(i,k\right),\left(k,i\right)\right\}$, with i,j,k a permutation from 1,2,3 and *j* the associated direction for the corresponding interaction being used from $H_{I}$ and $H_{{II}_{a,b}}$; $\left(i_{s},k_{s}\right)\in \left\{\left(j_{p},k_{p}\right),\left(k_{p},j_{p}\right);\left(0,i\right),\left(i,0\right)\right\}$, with $i,j_{p},k_{p}$ a permutation of parity *p* from 1,2,3 and $j_{p},k_{p}$ are the associated directions for the interaction $H_{III}$.

First, we should note that the signs for each term in the diagonal-off entries do not depend on the entries’ scripts in positions other than the parts in which the interaction is being applied, $k^{\prime },k^{\prime \prime }$ in the expressions of the previous section (30), (32), (34), and (36). This is because $\mathrm{Tr}\left({\tilde{\sigma }}_{i_{s}}^{*}\sigma_{j_{d+s}}{\tilde{\sigma }}_{k_{s}}^{T}\sigma_{j_{s}}^{T}\right)=\mathrm{Tr}\left({\tilde{\sigma }}_{i_{s}}^{*}\sigma_{0}{\tilde{\sigma }}_{k_{s}}^{T}\sigma_{0}^{T}\right)=2$ . Instead, signs only depend on the type of exchange indexes shown in Table 3. It has already been noted that $c_{j_{s},j_{d+s}}^{i_{s},k_{s}}$ is imaginary only if $j_{s}=2$ or $j_{d+s}=2$. This property is then transferred to the corresponding $F_{j,s}^{j_{s},j_{d+s}}$, and then transformed to $h_{12}$ as a function of the number of those factors in (31), (33), (35), and (37). Thus, by exchanging $i_{s},k_{s}$ (block transposing), only the cases with $h_{12}\in I$ will change their sign.

The final fact is related with the different signs appearing in the terms of diagonal-off entries. This will be important to analyze the number of independent blocks in the entire evolution matrix. For $H_{I}$ and $H_{III}$, the two different terms are obtained by the exchange of $j_{s},j_{d+s}$. Thus, for $H_{I}$, only the $c_{j_{s},j_{d+s}}^{i_{s},k_{s}}$ with $\left(j_{s},j_{s+d}\right)=\left(0,2\right),\left(2,0\right)$ and $\left(i_{s},k_{s}\right)\in \left\{\left(0,2\right),\left(2,0\right)\right\}$ or $\left(j_{s},j_{s+d}\right)=\left(0,j\neq \;2\right),\left(j\neq \;2,0\right)$ and $\left(i_{s},k_{s}\right)\in \left\{\left(i,k\right),\left(k,i\right)\right\}$ will change their sign (in the first four rows of Table 3). For $H_{III}$, if $\left(j_{s},j_{s+d}\right)=\left(1,3\right),\left(3,1\right)$ and $\left(i_{s},k_{s}\right)\in \left\{\left(0,2\right),\left(2,0\right)\right\}$ or $\left(j_{s},j_{s+d}\right)=\left(0,2\right),\left(2,0\right)$ and $\left(i_{s},k_{s}\right)\in \left\{\left(i,k\right),\left(k,i\right)\right\}$, then $c_{j_{s},j_{d+s}}^{i_{s},k_{s}}$ will change their sign (in the last four rows of Table 3). For $H_{IIa,b}$, two terms in the scripts are involved, so different aspects contribute: the location of interacting parts, the type of exchange, and their order in the scripts.

Last properties exhibits the way in which each term in $h_{12}$ will change its sign. The three aspects mentioned in the previous paragraph allow us to understand the diagonal-off structure of $H_{I},H_{IIa,b}$, and $H_{III}$ (considering that their diagonal components follow the properties discussed above). In the following subsections, we analyze this structure for each interaction, particularly discussing the independence of blocks in terms of the free parameters, making a distinction between the effective parameters (those appearing in the final expression of (28)) and the physical parameters (those appearing as coefficients $h_{I}$ in the Hamiltonian). They are not the same because many physical parameters appear clustered in the same way in (28), because the entries of $S_{U}$ depend only on the parameters $h_{11}\pm h_{22},h_{12}$. As a result, by grouping finally in the U(1)×SU(2) blocks, there will be only two or eight different blocks $S_{U}$ in *U*.

#### 5.4.3. Block Entries of $H_{I}$

The diagonal-off entries have exactly the two terms $h_{{\left(j4^{s+dt-1}\right)}_{4}^{2d}}$ for $t^{\prime }=0,1$, and there are only two combinations: adding or subtracting terms. As was stated previously, they are imaginary only if local interactions are in the direction j=2. In this case, we separate the factor ±i for j=2 cases in the diagonal-off entries, and the remaining coefficients in the opposite corners in each block are equal as expected from (28). Then, there is generally one term with the same sign through all diagonal-off entries (when k=2, or otherwise when $j_{s}=2$ in the first four rows in the Table 3), leaving only two possibilities for the remaining term. Thus, in each $H_{I}$ matrix there are blocks with only two different diagonal-off entries, depending only on the index exchange type in the local interaction position and not on the remaining indexes. Thus, for a fixed set of indexes for the positions unrelated to the part on which the interaction is applied, a pair of blocks exists, one each for the exchanges (0,j),(j,0) and (i,k),(k,i), with different relative signs in their diagonal-off terms. For the corresponding diagonal entries, in (29), only the difference $h_{11}-h_{22}$ is relevant. As previously stated by analyzing equation (31), it is also possible realize that in each diagonal entry there are only two terms from the 3d terms changing their sign with respect to other rows. Block scripts differ in only one index, those corresponding with $i^{\prime }\neq j$ (the local interaction direction) and $k=k^{\prime }$ (the correspondent pair on which the local interaction is being applied), leaving only two terms and two different combinations for $h_{11}-h_{22}$. This implies that there are only two different blocks for (29) through all *U*, each one operating with different exchange rules, (0,j),(j,0) or (i,k),(k,i). Each one is the same (until unitary factors, which can be different) for all entries with different indexes in positions other than $k^{\prime }$. This fact can be attributed, depending on the number of disposable parameters (five, including the time and excluding the parameters in the unitary factor of each block), to the independence between the two types of blocks in the evolution matrix (29).

#### 5.4.4. Block Entries of $H_{IIa}$

For the non-diagonal entries, because the exchange factor $F_{j,s}^{j_{s},j_{d+s}}$ appears two times for each *j*, all of them are real, so the opposite corners of each block are always equal. Each entry has four terms with alternating signs, in agreement with the Table 3, as a function of the rows’ subscripts. Signs only depend on both indexes exchanged: either they are the same type (0,j),(j,0) or (i,k),(k,i), or otherwise opposite with an exchange of each type. This will give only four sign combinations (a calculation not developed explicitly here), except for j=2, where the appearance of two factors *i* will change the overall factor, giving eight combinations, one half of them with opposite overall sign to the remaining. For the diagonal entries, based on the ideas in the previous case for $H_{I}$, there will be four terms changing their relative signs with respect to other associated diagonal entries in the same block, but now differing in two part indexes (due to the related non-local interaction). As before, one term has a fixed sign, so there are only eight combinations for the three remaining terms from the 16 possible. This means eight different combinations for $h_{11}-h_{22}$ in (29), due to the values $k=k^{\prime },k^{\prime \prime }$ for the non-correspondent parts with non-local interactions in $H_{D}$ for this case. Thus, similar to $H_{I}$, in this case there will be eight different blocks in *U* for (29): one for each one of the eight different combinations of the exchange rules. Each type applies in the same way for all entries with different indexes in positions other than $k^{\prime },k^{\prime \prime }$. There are nine free parameters, including the time and excluding the parameters in the unitary factor for the block, so independence among the eight types of blocks can be more elusive. Despite all this, located operations not involving all GBS basis states appear as achievable.

#### 5.4.5. Block Entries of $H_{IIb}$

Although the exchange factors $F_{j,s}^{j_{s},j_{d+s}}$ are crossed and *j* takes two different values in the subscripts, the discussion regards certain similitude to that for $H_{IIa}$. For the diagonal-off entries, in agreement with Table 3, it implies that only if j=2 is not included in the crossed interaction (i=2 in (35)) will they become real. Each entry will have four terms with alternating signs, in agreement with the outcomes of products of exchange factors in Table 3 as a function of the rows’ subscript involved. Here, there will be eight combinations (four and four with opposite overall signs), except for j=2 with only four combinations. For the diagonal entries, $h_{11}-h_{22}$ in (29), the situation is identical to $H_{IIa}$. Then, there will be eight different block types in *U* for each combination of exchange rules on the indexes $k^{\prime },k^{\prime \prime }$. Again, nine free parameters for the SU(2) blocks are available.

#### 5.4.6. Block Entries of $H_{III}$

This is a special case exception of the previous remark where $j_{s},j_{d+s}$ is not of the forms 0,j or j,0. Nevertheless, $F_{i,s}^{j_{p},k_{p}}$ becomes in the same way on of $c_{j_{p},k_{p}}^{0,i}$, $c_{j_{p},k_{p}}^{i,0}$, $c_{j_{p},k_{p}}^{j_{p},k_{p}}$, or $c_{j_{p},k_{p}}^{k_{p},j_{p}}$. However, several aspects are identical to the $H_{I}$ case. A brief analysis shows that entries become real only for i=2 (see the last four rows of Table 3). Each diagonal-off entry has two terms with alternating signs as functions of entry labels. For the diagonal entries, again only two types of terms change their sign in $H_{D}$ from (31) for the rows forming the SU(2) blocks with the exchange rules. This gives only two types of $h_{11}-h_{22}$ in (29), again generating only two different blocks in the whole *U*—each one for a kind of exchange rule involved here, containing five free parameters.

**Figure 5:**
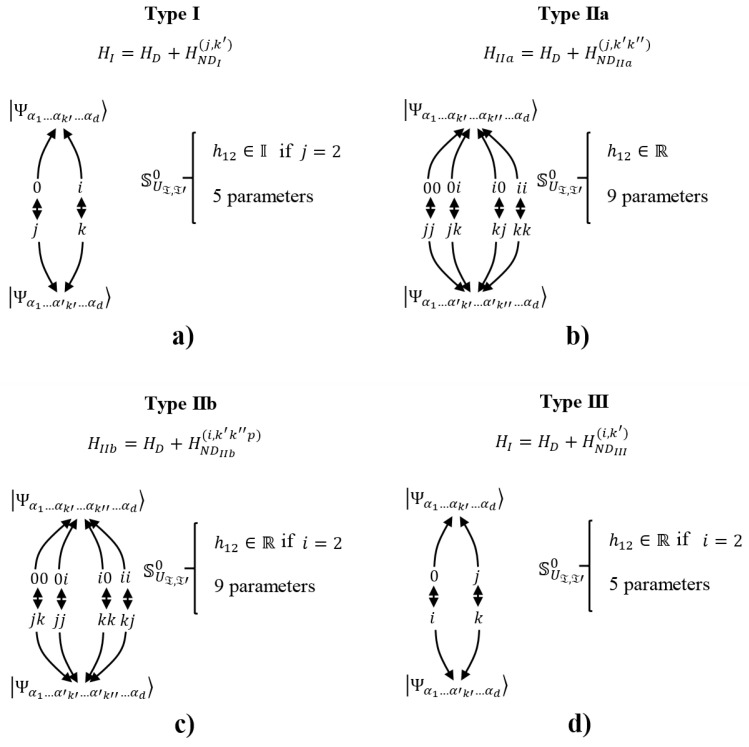
Exchange index relations involved for each interaction and highlighted properties for their correspondent $S_{U_{I,I^{\prime }}}^{0}$: (**a**) $H_{I}$; (**b**) $H_{IIa}$; (**c**) $H_{IIb}$; and (**d**) $H_{III}$. Exchange relations in (**b**,**d**) are doubled by considering the vertical switching in one of the indexes for each pair shown.

To resume the findings, Figure 5 shows the relations exhibited in the exchange indexes for each interaction. This figure depicts each of the exchange index relations of GBS basis states under the interaction. Thus, Figure 5a,d, depicts the two groups of exchange states for $H_{I}$ and $H_{III}$ generated by the two different blocks through the whole $SU(2^{2d})$ evolution matrix, both independent up to five parameters and with $h_{12}$ in (29). Figure 5b,c depict the double exchange indexes induced by the eight blocks generated by $H_{IIa}$ and $H_{IIb}$. These eight blocks are independent up to nine parameters. All representations in Figure 5 are for a single GBS basis state, but clearly one specific block is operating on any of them simultaneously. Note finally that for all cases there are a complementary number of free physical parameters in $h_{11}+h_{22}$: 3d+2−4=3d−2 for $H_{I}$ and $H_{III}$ and 3d+4−8=3d−4 for $H_{IIa}$ and $H_{IIb}$ (time *t* is not accounted because it was considered in the SU(2) fitting). Then, there is a linearly growing space to fit the blocks into a programmed operation in terms of the physical parameters, although there is an exponential growth of those blocks.

## 6. Connectedness, Superposition, Entanglement and Separability

To understand how dynamics is addressed under the interactions $H_{I},H_{IIa,b},H_{III}$ (used independently or combined), some complementary analysis is convenient. In order to prepare the reader, some illustrative examples are included in Appendix A.4 for d=1 and d=2, depicting some notable properties of dynamics in such cases by including several kinds of entangling operations.

### 6.1. Exchange Connectedness under Interactions

**Figure 6:**
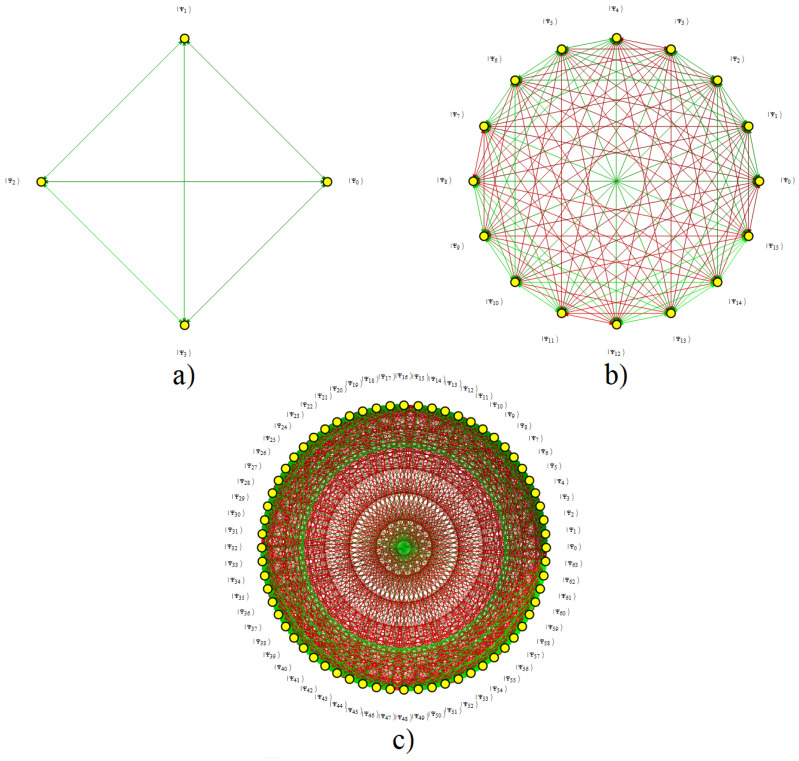
Connectedness graphs between states under SU(2) decomposition for one (green) and two (red) exchange scripts for all generalized Bell state (GBS) basis states: (**a**) d=1; (**b**) d=2; and (**c**) d=3.

Under the SU(2) decomposition, pairs of states in GBS basis become related, showing a probability exchange between them. As it was seen, each one of the $H_{I},H_{IIa,b},H_{III}$ interactions has rules for this exchange. In any case, it should be clear this exchange is achievable between any pair by combining all types of interactions obtained by switching the value of: (a) interaction direction and correspondent pair $j,k^{\prime }$ in (31) for $H_{I}$; (b) interaction direction and correspondent pairs $j,k^{\prime },k^{\prime \prime }$ in (33) for $H_{IIa}$; (c) interaction directions, correspondent pairs, and parity $i,k^{\prime },k^{\prime \prime },p$ in (35) for $H_{IIb}$; and (d) interaction direction and correspondent pair $i,k^{\prime }$ in (37) for $H_{III}$. Several types of interactions can be combined in a sequence. The combination of interactions is not precise for the basis element connectedness, but it is necessary to increase the entanglement, and thus to connect two arbitrary quantum states. In those terms, there are only two types of states: (1) those exchanging one script ($H_{I}$ and $H_{III}$), and (2) those exchanging two scripts ($H_{IIa}$ and $H_{IIb}$) in the GBS basis elements under the rules depicted in Figure 5 (although the rules and connections are different). All basis states become connected under one or several interactions applied consecutively, depending on the number of necessary exchanges in their scripts. Figure 6 shows a graph with these relations for the cases d=1,2,3. Green edges indicate one script exchange and red lines indicate two script exchanges. The connection can only be achieved with a single interaction in the first two cases, due to the low entanglement level. Figure 6a corresponds to the figure presented in [11] for Bell states in SU(4) systems.

Connectedness in a finite number of steps by applying some or all cases in each type of interaction (piecewise with constant parameters or with time-dependent parameters in each case) warrants the full probability exchange between the occupancy level of each state in terms of the discussion included in Section 3. Nevertheless, not all interactions are able to reach an arbitrary evolution. As is obvious, $H_{I}$ and $H_{III}$ are not able to generate extended entanglement out of the correspondent pair on which they operate (this assumes no rearrangements are made in the correspondent pairs and their elements). We discuss this aspect in the next subsection.

### 6.2. Notable Quantum Processing Operations Achievable under SU(2) Decomposition

Departing from $S_{U}$, then by fixing $\omega t=\frac{2n+1}{2}\pi ,\frac{h_{11}-h_{22}}{2\hbar \omega }=\epsilon ,\frac{h_{12}}{\hbar \omega }=i^{c}\delta ,\frac{h_{11}+h_{22}}{2\hbar \omega }=2\left(m-\frac{1}{2}\right);n,m\in Z,$ c∈{0,1},δ∈R, where $\epsilon^{2}+\delta^{2}=1$ in (29). Note that the parameter *c* depends on each kind of interaction in the terms discussed in the previous section. Then, we get the ${S_{U}}_{I,I^{\prime }}$ block [27]:

(38) $$\begin{array}{ccc} {H_{m}^{c}\left(\delta ,\epsilon \right)}_{I,I^{\prime }} & \equiv & {\left(-1\right)}^{m}\left(\begin{array}{cc} \epsilon & i^{c}\delta \\ {\left(-i\right)}^{c}\delta & -\epsilon \end{array}\right), \end{array}$$

operating on the GBS basis. Note that this form cannot always be achieved independently in all blocks in terms of the free parameters and the possible restriction $h_{0,0,\ldots ,0}=0$ (here $\det({H_{m}^{c}\left(\delta ,\epsilon \right)}_{I,I^{\prime }})=-1$, although it is not decisive in the following development). Nevertheless, we need only achieve it in some blocks in the immediate discussion. We are using the time-independent case, but other more practical cases with time-dependent Hamiltonian coefficients can be implemented. The last form is highly versatile. If $s_{\epsilon }\left|\epsilon \right|=\delta =\frac{1}{\sqrt{2}}$ ($s_{\epsilon }=\mathrm{sign}\left(\epsilon \right)$, referred in the notation as −,+), we get a Hadamard-like gate $H_{I,I^{\prime }}^{m,c,\mathrm{sign}(\epsilon )}\equiv {S_{U}}_{I,I^{\prime }}$ (in particular, if c=0, but this condition can be relaxed). When δ=1, we get an exchange-like gate [12,26] for the pair in the SU(2) block, $E_{I,I^{\prime }}^{m,c}\equiv {S_{U}}_{I,I^{\prime }}$. Note that this case is a limit case for the time-independent case (29) when $h_{12}\gg h_{11}-h_{22}$. Otherwise, it can be achieved in two steps of time-independent piecewise Hamiltonians (as in [12]) or as a continuous time-dependent Hamiltonian. These gates are:

(39) $$\begin{array}{c} H_{I,I^{\prime }}^{m,c,s_{\epsilon }}=\frac{{\left(-1\right)}^{m}}{\sqrt{2}}\left(\begin{array}{cc} s_{\epsilon } & i^{c}\\ {\left(-i\right)}^{c} & -s_{\epsilon } \end{array}\right),\;E_{I,I^{\prime }}^{m,c}={\left(-1\right)}^{m}\left(\begin{array}{cc} 0 & i^{c}\\ {\left(-i\right)}^{c} & 0 \end{array}\right). \end{array}$$

Note additionally that when $\frac{h_{11}+h_{22}}{2\hbar \omega }=\left(\frac{\alpha }{m}-1\right)\pi ,\omega t=m\pi ;n,m\in Z$, we get the quasi-identity gate ${S_{U}}_{I,I^{\prime }}=e^{i\alpha \pi }I_{I,I^{\prime }}\equiv I_{I,I^{\prime }}^{\alpha }$. The combination of these blocks (allowed because the block independence previously discussed) allows important quantum processing operations to be set.

### 6.3. SU(2) Decomposition in the Context of n-Qubit Controlled Gates

Transformation between quantum states can generally be achieved by means of linear and anti-linear operators. Anti-linear operators are particularly useful to depict time-reversal operations or the action of some Einstein-Podolsky-Rosen channels. If these kinds of operations are being considered in the processing, an extension of the Hamiltonian (1) should be considered by the inclusion of anti-linear operations [28]. In this work, we have restricted our development to linear operators, as was settled in Section 2 and Section 3.

Below of such context, it should be advised that SU(2) decomposition is compatible with the most quantum information developments in the literature. Nevertheless, many of those works do not consider that such proposed processing forms are rarely compatible with the dynamics of physical systems if the computational basis continues to be used (the natural basis based on physical properties of local systems such as spin and polarization). The nature of entangling operations naturally induces both superposition and entanglement, thus generating a complex dynamics evolution in such basis compared with the structured gates proposed in the quantum information developments (whose authors were clearly not always concerned with the underlying physics). SU(2) decomposition (mainly the part developed in the Section 2 and Section 3) naturally proposes a better basis to set the quantum processing grammar for certain interaction architectures (e.g., those developed in Section 4 and Section 5). The induced 2×2 block structure allows such processing structures to be set more easily, mainly based on binary processing.

In the context of quantum computation, the most common trend is the settlement of universal gates in the sense of a quantum Turing machine. A set of universal quantum gates for two-qubit processing was established by [29] as a set of local gates together with the CNot gate. Despite universality, this trend is not optimal because for a given processing, it is not clear how to express it in terms of those elements in the universal set. In an alternative trend, [30] has settled a more optimal gate decomposition by factorization in terms of P-unitary matrices. In the last two trends, SU(2) decomposition for SU(4) (d=1, meaning two-qubit processing) has shown how to adapt those results for the physics of Heisenberg–Ising interactions including driven magnetic fields: (a) in [31], a set of alternative universal gates has been proposed on the grammar of Bell states; and (b) in [26], an optimal set of six gates (P-unitary matrices) is proposed using the forms of SU(2) decomposition on a Bell states basis to reproduce any other gate for two-quibit processing. In the current context, those outcomes are automatically applicable to Type I and III interactions. Type II interactions are excluded because they require at least d=2. In any case, the contribution of the SU(2) reduction is in the proposal of Bell basis as a grammar instead of the computational one so that the physical evolution fulfills the forms required by the processing gates.

Although two-qubit processing is still universal, more powerful processing is possible by attaining more than two qubits at a time. In this approach, [32,33] have stated universal processing gates in terms of local rotations and n-qubits controlled gates. In the computational basis, rotations are obtained by local interactions by turning off the entangling operations, but controlled gates can be physically difficult to reproduce. In the SU(2) reduction scheme, the form of rotations in those works ($R_{y}\left(\alpha \right)$ and $R_{z}\left(\alpha \right)$) are achieved by the forms (29) as follows. First, $R_{y}\left(\alpha \right)$ is mainly achieved by settling $h_{11}=h_{22}$ and $h_{12}\in I$. $R_{z}\left(\alpha \right)$ is obtained by fixing $h_{12}=0$. Notably, those rotations are not necessarily physical neither local, they could operate among entangled states. Instead, they can be determined as rotations on the informational states being used (elements of GBS basis). Other basic forms are also easily obtained, for example, Ph(δ) is obtained by settling cosωt=±1. For the controlled gates $\Lambda_{n}\left(U\right)$ proposed in [32], authors in [33] turn to a long factorization in terms of rotations and controlled gates $\Lambda_{1}\left(U\right)$ (which can also be obtained departing from the CNot gate and rotations). In any case, if a computational basis is used, the reproduction of the CNot gate can still bring certain difficulties in many quantum systems [34]. In the context of SU(2) reduction, CNot gate and inclusively $\Lambda_{1}\left(U\right)$ are directly obtained if the Bell basis is used as grammar:

(40) $$\begin{array}{ccc} \Lambda_{1}\left(U\right) & = & \left(\begin{array}{cc} {S_{U}}_{1}\to I & 0\\ 0 & {S_{U}}_{2}\to U \end{array}\right), \end{array}$$

where *U* is a general matrix in SU(2) as in (29). Because of the independence of blocks stated in Section 5, the achievement of $\Lambda_{1}\left(U\right)$ is warranted. Then, the construction of $\Lambda_{n}\left(U\right)$ follows immediately as proposed in [32,33], but considering those forms working on the grammar basis of the Bell states or on the GBS basis in general. Clearly, in the SU(2) decomposition scheme, other controlled gates are achievable by the alternative selection of the elements on which interaction is being applied. If more optimal factorization methods are possible for d>1 (where blocks are repeated by groups), based on the set of matrices *U* as in (14) by including all the possible forms generated by Type I, IIa, IIb, and III interactions, it is still an open question.

### 6.4. Generating Superposition and Entanglement

In the following, we will use an arrow to depict a certain group of quantum operations. On the top of the arrow, we set the type of interaction being used. On the bottom, we set the subspace on which they apply or the generic form of each operation, together with their prescriptions. For instance, if an operation for d=4 (8 qubits and 256 elements in the GBS basis) generated by the Type IIa interaction is applied in the associated direction *y* and on the pairs 1 and 4 ($j=2,k^{\prime }=2,k^{\prime \prime }=4$ in (32)) with prescriptions for a Hadamard gate mixing the basis states $|\Psi_{0}\rangle =|\Psi_{0,0,0,0}\rangle$ and $|\Psi_{130}\rangle =|\Psi_{2,0,0,2}\rangle$ (i.e., $H_{0,130}^{0,0,+}$) and an exchange gate between the basis states $|\Psi_{1}\rangle =|\Psi_{1,0,0,0}\rangle$ and $|\Psi_{131}\rangle =|\Psi_{3,0,0,2}\rangle$ (i.e., $E_{0,131}^{0,0}$), we will write:

(41) $$\begin{array}{c} \begin{array}{c} H_{IIa}^{\left(2,2,5\right)}\\ \overset{\;}{\to }\\ H_{0,130}^{0,0,+}⊕E_{0,131}^{0,0} \end{array}. \end{array}$$

Although other operations can be defined between the remaining basis states, if they are not specified, it is because some operations are repeated for other certain groups of scripts (e.g., for $|\Psi_{20}\rangle =|\Psi_{0,1,1,0}\rangle$ and $|\Psi_{150}\rangle =|\Psi_{2,1,1,2}\rangle$, $H^{0,0,+}$ is also being applied) or because the concrete operation being developed does not require such specification (e.g., there is no specification for the operation between $|\Psi_{67}\rangle =|\Psi_{3,0,0,1}\rangle$ and $|\Psi_{193}\rangle =|\Psi_{1,0,0,3}\rangle$). In some cases, complex families of subsequent operations are required, and then one family is specified by a group of indexes defining it.

#### 6.4.1. Generating 2-Separable Superposition

**Figure 7:**
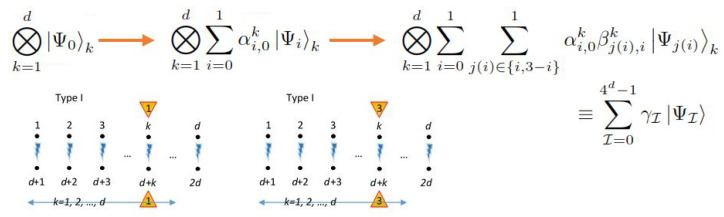
Processes to build 2-separable states with complete superposition.

By using the general block operations ${S_{U}}_{I,I^{\prime }}\in U\left(1\right)\times SU\left(2\right)$ (29), it is possible to arrive at a state exhibiting complete superposition through all of the basis elements. Thus, for example, departing from the simple state ${|\Psi_{0}\rangle }^{2d}={|\Psi_{0}\rangle }_{1}{|\Psi_{0}\rangle }_{2}\ldots {|\Psi_{0}\rangle }_{d}$ (easily obtained from $|00\ldots 0\rangle$), a couple of local operations $H_{I}^{\left(i,k\right)}$ on each correspondent pair *k* are sufficient to generate a state containing representatives from each basis element:

(42) $$\begin{array}{cc} {|\Psi_{0}\rangle }^{2d}\begin{array}{c} H_{I}^{\left(1,k\right)}\\ k=1,2,\ldots ,d\\ \overset{\;}{\to }\\ \underset{s,s^{\prime }}{⨁}{S_{U}}_{s,s^{\prime }}\\ s^{\prime }-s=4^{k-1},\\ s_{4,k}^{d}=0 \end{array} & \overset{d}{\underset{k=1}{⨂}}\sum_{i=0}^{1}\alpha_{i,0}^{k}{|\Psi_{i}\rangle }_{k},\\ \begin{array}{c} H_{I}^{\left(3,k\right)}\\ k=1,2,\ldots ,d\\ \overset{\;}{\to }\\ \underset{s,s^{\prime }}{⨁}{S_{U}}_{s,s^{\prime }}⊕\underset{s^{\prime \prime },s^{\prime \prime \prime }}{⨁}{S_{U}}_{s^{\prime \prime },s^{\prime \prime \prime }}\\ s^{\prime }-s=3\cdot 4^{k-1},s^{\prime \prime \prime }-s^{\prime \prime }=4^{k-1}\\ s_{4,k}^{d}=0,{s^{\prime \prime }}_{4,k}^{d}=1 \end{array} & \overset{d}{\underset{k=1}{⨂}}\sum_{i=0}^{1}\sum_{j(i)\in \{i,3-i\}}^{1}\alpha_{i,0}^{k}\beta_{j(i),i}^{k}{|\Psi_{j(i)}\rangle }_{k}\equiv \sum_{I=0}^{4^{d}-1}\gamma_{I}|\Psi_{I}\rangle ,\\ \mathrm{with}: & \gamma_{I}=\prod_{k=1}^{d}\alpha_{j^{-1}\left(I_{4,k}^{d}\right),0}^{k}\beta_{I_{4,k}^{d},j^{-1}\left(I_{4,k}^{d}\right)}^{k}, \end{array}$$

where $j^{-1}\left(i\right)$ is the inverse of j(i) and directions i=1,3 were used as examples. In addition, $\alpha_{i,j}^{k}$ are the components of ${S_{U}}_{s,s^{\prime }}$ in the first operations, and $\beta_{j(i),i}^{k}$ are the components of ${S_{U}}_{s,s^{\prime }}$, ${S_{U}}_{s^{\prime \prime },s^{\prime \prime \prime }}$ for the second operations with i=0,1, respectively. Figure 7 depicts each step of the process, using the local operations (alternatively, crossed interactions in $H_{III}$ could be considered).

The last process is a particular case of more general operations by considering $O_{J}^{\left(i,\{s\}\right)}={S_{U}}_{I,I^{\prime }}$ to mix the states through the momentary associated blocks changing the indexes {s} with some interaction $H_{J},J\in \left\{I,IIa,IIb,III\right\}$ in the associated direction *i*. We coin the term k-local operation when ${S_{U}}_{I,I^{\prime }}$ generates entanglement at the most in *k* parts. In our basic interactions scheme, there are only 2-local and 4-local operations, as was discussed previously. Thus, following the previously-introduced notation, we set a family of procedures to develop superposition including the previous procedure. Departing from the ${|\Psi_{0}\rangle }^{2d}$, it is possible to apply several alternate 2-local operations to generate superposition involving all GBS basis states. By defining a sequence of paired directions for the $H_{I}$ evolution involving all pairs s=1,2,…,d (this process can alternatively be achieved by $H_{III}$): $\left\{\left\{i_{s},k_{s}\left(i_{s}\right)\right\}|\left\{1,2,3\right\}∋i_{s}\neq k_{s}\left(i_{s}\right)\in \left\{1,2,3\right\}\backslash \left\{i_{s}\right\};s=1,2,\ldots ,d\right\}$. Additionally, $j_{s}\left(i_{s}\right)\in \left\{1,2,3\right\},i_{s}\neq j_{s}\left(i_{s}\right)\neq k_{s}\left(i_{s}\right)$. Then, following the evolution process:

(43) $$\begin{array}{cc} {|\Psi_{0}\rangle }^{2d} & \begin{array}{c} H_{I}^{\left(i_{1},1\right)}\\ \overset{\;}{\to }\\ O_{I}^{\left(i_{1},\left\{1\right\}\right)} \end{array}\sum_{t\in \{0,i_{1}\}}\alpha_{t,0}^{1}|\Psi_{t,0,\ldots ,0}\rangle \begin{array}{c} H_{I}^{\left(k_{1}\left(i_{1}\right),1\right)}\\ \overset{\;}{\to }\\ O_{I}^{\left(k_{1}\left(i_{1}\right),\left\{1\right\}\right)} \end{array}\sum_{\epsilon_{1}=0}^{3}\alpha_{p_{1}\left(\epsilon_{1}\right),0}^{1}\beta_{\epsilon_{1},p_{1}\left(\epsilon_{1}\right)}^{1}|\Psi_{\epsilon_{1},0,\ldots ,0}\rangle ,\\ & \begin{array}{c} H_{I}^{\left(i_{2},2\right)}\\ \overset{\;}{\to }\\ O_{I}^{\left(i_{2},\left\{2\right\}\right)} \end{array}\ldots \begin{array}{c} H_{I}^{\left(k_{d}\left(i_{d}\right),d\right)}\\ \overset{\;}{\to }\\ O_{I}^{\left(k_{d}\left(i_{d}\right),\left\{d\right\}\right)} \end{array}\sum_{\epsilon_{1},\ldots ,\epsilon_{d}=0}^{3}(\prod_{s=1}^{d}\alpha_{p_{s}\left(\epsilon_{s}\right),0}^{s}\beta_{\epsilon_{s},p_{s}\left(\epsilon_{s}\right)}^{s})|\Psi_{\epsilon_{1},\epsilon_{2},\ldots ,\epsilon_{d}}\rangle \equiv |\Psi_{f_{\mathrm{sep}}}\rangle , \end{array}$$

where $p_{s}\left(\epsilon_{s}\right)$ are the inverses of the association rules for the one index exchanges depicted in Figure 5a (or Figure 5d for $H_{III}$): $p_{s}\left(i_{s}\right)=i_{s}=p_{s}\left(j_{s}\left(i_{s}\right)\right),p_{s}\left(0\right)=0=p_{s}\left(k_{s}\left(i_{s}\right)\right)$. Additionally, $|\alpha_{0,0}^{s}{|}^{2}+{\left|\alpha_{i_{s},0}^{s}\right|}^{2}=1,$ $|\beta_{0,0}^{s}{|}^{2}+|\beta_{k_{s}\left(i_{s}\right),0}^{s}{|}^{2}=1,|\beta_{i_{s},i_{s}}^{s}{|}^{2}+{\left|\beta_{j_{s}\left(i_{s}\right),i_{s}}^{s}\right|}^{2}=1$. ${\mathrm{Tr}}^{S}\left(\rho_{IJ}\right)$ represents the partial trace with respect to the entire system except the s∈S parts. As expected, $|\Psi_{f_{\mathrm{sep}}}\rangle$ is 2-separable:

(44) $$\begin{array}{c} {\mathrm{Tr}}^{\left\{k^{\prime }\right\}}\left(|\Psi_{f_{\mathrm{sep}}}\rangle \langle \Psi_{f_{\mathrm{sep}}}|\right)=(\sum_{\epsilon_{k^{\prime }}=0}^{3}\alpha_{p_{k^{\prime }}\left(\epsilon_{k^{\prime }}\right),0}^{k^{\prime }}\beta_{\epsilon_{k^{\prime }},p_{k^{\prime }}\left(\epsilon_{k^{\prime }}\right)}^{k^{\prime }}|\Psi_{\epsilon_{k^{\prime }}}\rangle )\left(\sum_{\epsilon_{k^{\prime }}=0}^{3}\alpha_{p_{k^{\prime }}\left(\epsilon_{k^{\prime }}\right),0}^{k^{\prime }}\beta_{\epsilon_{k^{\prime }},p_{k^{\prime }}\left(\epsilon_{k^{\prime }}\right)}^{k^{\prime }}|\Psi_{\epsilon_{k^{\prime }}}\rangle \right){}^{†} \end{array}$$

due to the limited nature of operations involved, which cannot be able to generate more extended entanglement. In addition, superposition can be limited to the SU(2) blocks coverage through the number of parameters introduced, $\alpha_{p_{s}\left(\epsilon_{s}\right),0}^{s},\beta_{\epsilon_{s},p_{s}\left(\epsilon_{s}\right)}^{s}$, and their physical scope. As shown in [11], a richer superposition coverage on $SU(2^{2d})$ can be achieved with additional 2-local operations on each part, introducing extra parameters and probability mixing. As in [11], n in (29) is limited to take the two forms $\left(n_{x},0,n_{z}\right)$ or $\left(0,n_{y},n_{z}\right)$ (for the time-independent case), but by combining both forms we arrive at two general forms with arbitrary $n=(n_{x},n_{y},n_{z})$ (this also fulfills the time-dependent case with adequate $h_{ij}\left(t\right)$).

Although this procedure can include a general full 2-separable state together with entangled segments between correspondent pairs, it cannot exhibit states with more extended entanglement, requiring more extended entangling operations such as $H_{IIa}$ and $H_{IIb}$. The quest is to obtain general states departing from a simple resource, which is still an open challenge—particularly for the possible entanglement degree there (a more ambitious challenge is the transformation between two general states [35], but it can always be reduced in two steps of this kind). We discuss this issue in the remaining subsection, and we develop some procedures to generate some maximal entangled states of arbitrary size.

#### 6.4.2. Entanglement Dynamics under Interactions

Now, we analyze the entanglement generation under the interactions being considered. We employ the partial trace criterion [19] for pure states by considering a single SU(2) combination of two GBS basis states $|\phi_{IJ}\rangle =\alpha_{I}|\Psi_{I}\rangle +\alpha_{J}|\Psi_{J}\rangle$. In addition, the explicit form for coefficients will be written as $\alpha_{I}=\cos\theta /2,\alpha_{J}=e^{i\phi }\sin\theta /2$. Then, we construct their associated density matrix $\rho_{IJ}=|\phi_{IJ}\rangle \langle \phi_{IJ}|$ to conveniently take partial traces in order to analyze the entanglement of specific subsystems in this quantum state under concrete interactions. Because the rules in the exchange scripts (in the GBS basis states to form the SU(2) blocks) are basically the same for the three interactions $H_{I},H_{IIa,b},H_{III}$, the analysis is reduced to only two cases. The first is for a pair of GBS basis elements $|\Psi_{I}\rangle ,|\Psi_{J}\rangle$ differing in only one subscript between I and J: $i_{s}=j_{s}\forall s\in \left\{1,\ldots ,d\right\},s\neq k^{\prime }$ (in $H_{I},H_{III}$ interactions). Thus, in this case (omitting the base *b* and the size *d* for simplicity in the scripts):

(45) $$\begin{array}{c} |\phi_{\mathrm{IJ}}^{1}\rangle =\frac{1}{\sqrt{2^{d}}}\sum_{E,D=0}^{2^{d}-1}\left(\overset{d}{\underset{k^{\prime }\neq s=1}{⨂}}{\tilde{\sigma }}_{i_{s}}⊗\left(\alpha_{I}{\tilde{\sigma }}_{i_{k^{\prime }}}+\alpha_{J}{\tilde{\sigma }}_{j_{k^{\prime }}}\right)\right){}_{E,D}|E\rangle ⊗|D\rangle . \end{array}$$

The second case is for a pair of elements in the GBS basis differing in two subscripts of I and J: $i_{s}=j_{s}\forall s=1,\ldots ,d,s\neq k^{\prime },k^{\prime \prime }$ (in $H_{IIa,b}$ interactions):

(46) $$\begin{array}{c} |\phi_{\mathrm{IJ}}^{2}\rangle =\frac{1}{\sqrt{2^{d}}}\sum_{E,D=0}^{2^{d}-1}\left(\overset{d}{\underset{k^{\prime },k^{\prime \prime }\neq s=1}{⨂}}{\tilde{\sigma }}_{i_{s}}⊗\left(\alpha_{I}{\tilde{\sigma }}_{i_{k^{\prime }}}⊗{\tilde{\sigma }}_{i_{k^{\prime \prime }}}+\alpha_{J}{\tilde{\sigma }}_{j_{k^{\prime }}}⊗{\tilde{\sigma }}_{j_{k^{\prime \prime }}}\right)\right){}_{E,D}|E\rangle ⊗|D\rangle . \end{array}$$

Then, we analyze the entanglement of several subsystems in each case by taking the partial trace with respect to its complement. Calculations are direct. At the end, the association rules 0↔i and j↔k should be applied to explicitly denote the viable relations between I and J, and to reduce some traces on parts $k^{\prime },k^{\prime \prime }$ in (45) and (46). In Table 4 we report the generalized bipartite concurrence for pure states [36]:

(47) $$\begin{array}{ccc} C^{2}\left({\mathrm{Tr}}^{S}\left(\rho_{IJ}\right)\right) & = & 2(1-{\mathrm{Tr}}^{S}\left(\rho_{IJ}^{2}\right)), \end{array}$$

where, *j* is assumed as the direction label of the interaction involved. If $m=\min(m_{1},m_{2})$, where $m_{1},m_{2}$ are the Hilbert space dimensions of each subsystem, then this measure changes smoothly from 0 for separable states to 2(m−1)/m for maximally entangled states. Note that we take ${\tilde{\sigma }}_{i}\equiv e^{i\phi_{i}}\sigma_{i}$, although it is only relevant for $\sigma_{2}$. With this distinction, we introduce $\phi^{\prime }=\phi +\phi_{i_{k}^{\prime }}-\phi_{j_{k}^{\prime }}$.

Table 4 includes some obvious results for “local” interactions on single parts ($H_{I}$) or on correspondent pairs ($H_{III}$): (a) any part is maximally entangled with respect to the remaining system (through its correspondent pair) if there are currently no active local or non-local crossed interactions in $H_{I}$ and $H_{III}$, respectively, so $C^{2}\left({\mathrm{Tr}}^{S}\left(\rho_{IJ}\right)\right)=1$; (b) nevertheless, if these local or non-local crossed interactions act on the correspondent pair, each part of it can become separable or partially entangled to the remaining system; and (c) any correspondent pair (as a subsystem) is separable from the remaining system in any GBS basis state, so $C^{2}\left({\mathrm{Tr}}^{S}\left(\rho_{IJ}\right)\right)=0$. Note that in the cases (b) and (c) that the subsystem comprises two parts $\left[k^{\prime },k^{\prime }+d\right]$ being compared with the remaining system, so the Hilbert space dimension is four (m=4). Similarly, the most important results here: (d) shows how interactions between non-correspondent parts (crossed or non-crossed) affect the original separability of each correspondent pair with respect to the remaining system, letting it become entangled with the remaining system. Finally, (e) exhibits the change of entanglement between non-correspondent parts. They are clearly originally entangled with their respective pair outside of the subsystem, but that entanglement becomes reduced ($C^{2}\left({\mathrm{Tr}}^{S}\left(\rho_{IJ}\right)\right)\leq 3/2$) due to the non-local interactions.

#### 6.4.3. Generating Larger Maximal Entangled Systems

The generation of extended entanglement can be shown with a couple of introductory examples [27]. If $|\beta_{ij}\rangle =|\Psi_{2i+i⊕j}\rangle$ are the GBS basis elements for d=1 corresponding to the Bell states [22], then considering the $|GHZ\rangle$ and $|W\rangle$ states of size 2d expressed in the GBS basis:

(48) $$\begin{array}{ccc} {|GHZ\rangle }^{2d} & = & \frac{1}{\sqrt{2}}\sum_{i=0}^{1}\overset{d}{\underset{j=1}{⨂}}{|i,i\rangle }_{j}=\frac{1}{2^{\frac{d+1}{2}}}\sum_{i=0}^{1}\overset{d}{\underset{j=1}{⨂}}\left({|\Psi_{0}\rangle }_{j}+{\left(-1\right)}^{i}{|\Psi_{3}\rangle }_{j}\right), \end{array}$$

(49) $$\begin{array}{ccc} {|W\rangle }^{2d} & = & \frac{1}{\sqrt{2d}}\sum_{i=1}^{2d}\overset{d}{\underset{j=1}{⨂}}{|\delta_{i,2j-1},\delta_{i,2j}\rangle }_{j}=\frac{d^{-\frac{1}{2}}}{2^{\frac{d-1}{2}}}\sum_{i=1}^{d}\overset{d}{\underset{\begin{array}{c} j=1\\ j\neq i \end{array}}{⨂}}\left({|\Psi_{0}\rangle }_{j}+{|\Psi_{3}\rangle }_{j}\right)⊗{|\Psi_{1}\rangle }_{i}, \end{array}$$

where *j* sums over correspondent pairs. Note that we are alternating the notation in the kets by convenience: ${|\Psi_{k}\rangle }_{j}$ is the Bell state $|\Psi_{k}\rangle$ on the $j^{\mathrm{th}}$ correspondent pair, while $|\Psi_{i_{1},i_{2},\ldots ,i_{d}}\rangle =|\Psi_{I}\rangle$ is the $I=4^{d-1}i_{d}+\ldots +4i_{2}+i_{1}$ element in the GBS basis. For d=2, they are simply:

(50) $$\begin{array}{ccc} {|GHZ\rangle }^{4} & = & \frac{1}{\sqrt{2}}\left(|\Psi_{0,0}\rangle +|\Psi_{3,3}\rangle \right)=\frac{1}{\sqrt{2}}\sum_{I\in \{0,15\}}|\Psi_{I}\rangle , \end{array}$$

(51) $$\begin{array}{ccc} {|W\rangle }^{4} & = & \frac{1}{2}(|\Psi_{1,0}\rangle +|\Psi_{0,1}\rangle +|\Psi_{3,1}\rangle +|\Psi_{1,3}\rangle )=\frac{1}{2}\sum_{I\in \{1,4,7,13\}}|\Psi_{I}\rangle . \end{array}$$

Then, we can depart from the basic state $|0000\rangle =\frac{1}{2}\left({|\Psi_{0}\rangle }_{1}+{|\Psi_{3}\rangle }_{1}\right)⊗\left({|\Psi_{0}\rangle }_{2}+{|\Psi_{3}\rangle }_{2}\right)$ for d=2. We arrive at the $|GHZ\rangle$ by applying the following operations (as before, the interaction Hamiltonian is indicated in the upper position, while the operation is written below):

(52) $$\begin{array}{cc} |0000\rangle & \begin{array}{c} H_{I}^{\left(3,1\right)}\\ \overset{\;}{\to }\\ H_{0,3}^{0,0,+}⊕H_{12,15}^{0,0,+} \end{array}\;\frac{1}{\sqrt{2}}{|\Psi_{0}\rangle }_{1}⊗\left({|\Psi_{0}\rangle }_{2}+{|\Psi_{3}\rangle }_{2}\right),\\ & \begin{array}{c} H_{I}^{\left(3,2\right)}\\ \overset{\;}{\to }\\ H_{0,12}^{0,0,+} \end{array}{|\Psi_{0}\rangle }_{1}⊗{|\Psi_{0}\rangle }_{2}=|\Psi_{0,0}\rangle ,\\ & \begin{array}{c} H_{IIa}^{\left(3,1,2\right)}\\ \overset{\;}{\to }\\ H_{0,15}^{0,0,+} \end{array}\;\frac{1}{\sqrt{2}}\left(|\Psi_{0,0}\rangle +|\Psi_{3,3}\rangle \right)=\frac{1}{\sqrt{2}}\left(|\Psi_{0}\rangle +|\Psi_{15}\rangle \right)={|GHZ\rangle }^{4}. \end{array}$$

The first operation requires action on two sets of GBS basis states. They are of the same form, so they are easily achieved in terms of prescriptions for $H_{I,I^{\prime }}^{m,c,s_{\epsilon }}$. Note that no more specifications are needed in complementary blocks. They are free because their effect will work on states that are not included. Similarly, for example:

(53) $$\begin{array}{cc} {|GHZ\rangle }^{4}\begin{array}{c} H_{IIa}^{\left(2,1,2\right)}\\ \overset{\;}{\to }\\ I_{0,10}^{0}⊕E_{5,15}^{0,0} \end{array} & \frac{1}{\sqrt{2}}\left(|\Psi_{0,0}\rangle +|\Psi_{1,1}\rangle \right),\\ \begin{array}{c} H_{I}^{\left(1,2\right)}\\ \overset{\;}{\to }\\ E_{0,4}^{0,0}⊕E_{1,5}^{0,0} \end{array} & \frac{1}{\sqrt{2}}\left({|\Psi_{0}\rangle }_{1}⊗{|\Psi_{1}\rangle }_{2}+{|\Psi_{1}\rangle }_{1}⊗{|\Psi_{0}\rangle }_{2}\right),\\ \begin{array}{c} H_{I}^{\left(3,1\right)}\\ \overset{\;}{\to }\\ H_{4,7}^{0,0,+}⊕I_{1,2}^{2p} \end{array} & \frac{1}{\sqrt{2}}\left(\frac{1}{\sqrt{2}}\left({|\Psi_{0}\rangle }_{1}+{|\Psi_{3}\rangle }_{1}\right)⊗{|\Psi_{1}\rangle }_{2}+{|\Psi_{1}\rangle }_{1}⊗{|\Psi_{0}\rangle }_{2}\right),\\ \begin{array}{c} H_{I}^{\left(3,2\right)}\\ \overset{\;}{\to }\\ I_{4,8}^{2q}⊕I_{7,11}^{2r}⊕H_{1,13}^{0,0,+} \end{array} & \frac{1}{2}\left(\left({|\Psi_{0}\rangle }_{1}+{|\Psi_{3}\rangle }_{1}\right)⊗{|\Psi_{1}\rangle }_{2}+{|\Psi_{1}\rangle }_{1}⊗\left({|\Psi_{0}\rangle }_{2}+{|\Psi_{3}\rangle }_{2}\right)\right)\\ = & \frac{1}{2}\left(|\Psi_{4}\rangle +|\Psi_{7}\rangle +|\Psi_{1}\rangle +|\Psi_{13}\rangle \right)={|W\rangle }^{4}, \end{array}$$

where p,q,r∈Z. In the last operations, the block independence discussed in the previous section was applied to justify the construction of some simultaneous operations.

#### 6.4.4. Recursive Generation of Larger Maximal Entangled Systems

In the previous subsection, we obtained the larger maximal entangled states ${|GHZ\rangle }^{4}$ and ${|W\rangle }^{4}$ departing from the more basic states such as $|0000\rangle$. The enlargement of entangled states can be stated in a more impressive way as recursive processes. In each case, these processes are based on the control of the parameters involved and the independence among block types generated in each interaction.

**Figure 8:**
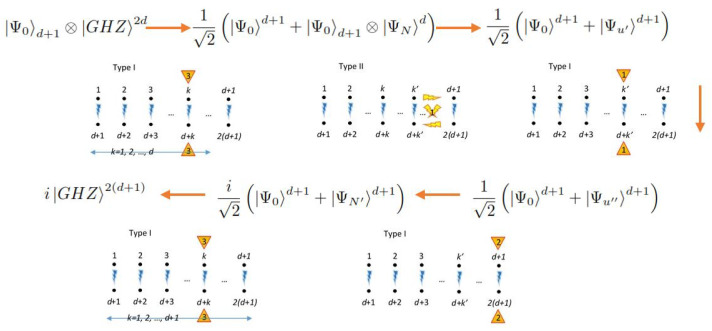
Processes to build recursive enlargement of $|GHZ\rangle$ entangled states.

Thus, the process shown in Figure 8 combines some of the operations depicted previously to develop ${|GHZ\rangle }^{2(d+1)}$ departing from ${|GHZ\rangle }^{2d}$, stating a procedure to get larger versions of these maximal entangled states. The first step begins by using the state ${|\Psi_{0}\rangle }_{d+1}⊗{|GHZ\rangle }^{2d}$. Then, a local operation is applied on each pair in the original state k=1,2,…,d to reduce the factors $\left({|\Psi_{0}\rangle }_{k}+{|\Psi_{3}\rangle }_{k}\right)$ and $\left({|\Psi_{0}\rangle }_{j}-{|\Psi_{3}\rangle }_{j}\right)$ in (48) into ${|\Psi_{0}\rangle }_{j}$ and ${|\Psi_{3}\rangle }_{j}$, respectively. Then, we exchange the indexes 30↔21 for the non-correspondent pairs $k^{\prime }$ and d+1 with a non-local operation. This transformation is followed by a couple of local operations changing the indexes 2↔3 for the pair $k^{\prime }$ and 1↔3 for the pair d+1 (which adds a factor *i*). In this last case, we transform the index 0 by itself, but adding the factor *i* . Finally, we revert for k=1,2,…,d+1 the initial transformation between ${|\Psi_{0}\rangle }_{k},{|\Psi_{3}\rangle }_{k}$ and $\left({|\Psi_{0}\rangle }_{k}\pm {|\Psi_{3}\rangle }_{k}\right)$, respectively. All additional index transformations are settled as the identity. The state obtained will be $i{|GHZ\rangle }^{2(d+1)}$. It is notable that only one 4-entangling operation between the added pair with another arbitrary pair from the original 2d-partite system has become necessary in this case. This reflects the low robustness of the genuine entanglement for these states. Considering the expression for ${|GHZ\rangle }^{2d}$ in (48), the precise prescriptions are:

(54) $$\begin{array}{cc} {|\Psi_{0}\rangle }_{d+1}⊗{|GHZ\rangle }^{2d}\;\begin{array}{c} H_{I}^{\left(3,k\right)}\\ k=1,2,\ldots ,d\\ \overset{\;}{\to }\\ \underset{s,s^{\prime }}{⨁}H_{s,s^{\prime }}^{0,0,+}\\ s^{\prime }-s=3\cdot 4^{k-1},\\ s,s^{\prime }\in \left\{3p\leq N|p\in N\right\} \end{array} & \frac{1}{\sqrt{2}}\left({|\Psi_{0}\rangle }^{d+1}+{|\Psi_{0}\rangle }_{d+1}⊗{|\Psi_{N}\rangle }^{d}\right),\\ \begin{array}{c} H_{IIa}^{\left(1,k^{\prime },d+1\right)}\\ \overset{\;}{\to }\\ I_{0,u}^{0}⊕E_{N,u^{\prime }}^{0,0}\\ u=4^{k^{\prime }-1}+4^{d}\\ u^{\prime }=N-4^{k^{\prime }-1}+4^{d} \end{array} & \frac{1}{\sqrt{2}}\left({|\Psi_{0}\rangle }^{d+1}+{|\Psi_{u^{\prime }}\rangle }^{d+1}\right),\\ \begin{array}{c} H_{I}^{\left(1,k^{\prime }\right)}\\ \overset{\;}{\to }\\ I_{0,4^{k^{\prime }-1}}^{0}⊕E_{u^{\prime },u^{\prime \prime }}^{0,0}\\ u^{\prime \prime }=u^{\prime }+4^{k^{\prime }-1} \end{array} & \frac{1}{\sqrt{2}}\left({|\Psi_{0}\rangle }^{d+1}+{|\Psi_{u^{\prime \prime }}\rangle }^{d+1}\right),\\ \begin{array}{c} H_{I}^{\left(2,d+1\right)}\\ \overset{\;}{\to }\\ I_{0,2\cdot 4^{d}}^{\frac{1}{2}}⊕E_{u^{\prime \prime },N^{\prime }}^{0,1} \end{array} & \frac{i}{\sqrt{2}}\left({|\Psi_{0}\rangle }^{d+1}+{|\Psi_{N^{\prime }}\rangle }^{d+1}\right),\\ \begin{array}{c} H_{I}^{\left(3,k\right)}\\ k=1,2,\ldots ,d+1\\ \overset{\;}{\to }\\ \underset{s,s^{\prime }}{⨁}H_{s,s^{\prime }}^{0,0,+}\\ s^{\prime }-s=3\cdot 4^{k-1},\\ s,s^{\prime }\in \left\{3p\leq N^{\prime }|p\in N\right\} \end{array} & i{|GHZ\rangle }^{2(d+1)}, \end{array}$$

where ${|\Psi_{I}\rangle }^{n}={|\Psi_{i_{1}}\rangle }_{1}⊗{|\Psi_{i_{2}}\rangle }_{2}⊗\ldots ⊗{|\Psi_{i_{n}}\rangle }_{n}$. In addition, $N=4^{d}-1$ and $N^{\prime }=4^{d+1}-1$. Note that the first and last operations are actually a set of operations for k=1,2,…,d and k=1,2,…,d+1 through several correspondent pairs. They exploit the Hadamard-like block operations for $H_{I}^{\left(3,k\right)}$ to switch first the ${|GHZ\rangle }^{2d}$ into versions where only the states $|\Psi_{0}\rangle$ and $|\Psi_{3}\rangle$ appear. Thus, operations generated with $H_{IIa}^{\left(1,k^{\prime },d+1\right)}$ between two different correspondent pairs are used as exchange operations entangling the added state ${|\Psi_{0}\rangle }_{d+1}$. Then, the additional operations $H_{I}^{\left(1,k^{\prime }\right)}$ and $H_{I}^{\left(2,d+1\right)}$ generate a state expressed only in terms of $|\Psi_{0}\rangle$ and $|\Psi_{3}\rangle$, to finally be transformed into ${|GHZ\rangle }^{2(d+1)}$ with the same kind of initial operations.

**Figure 9:**
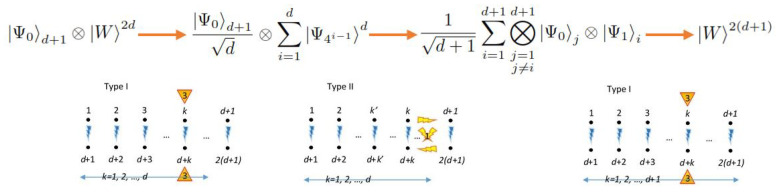
Processes to build recursive enlargement of $|W\rangle$ entangled states.

To obtain the ${|W\rangle }^{2(d+1)}$ state, we begin with ${|\Psi_{0}\rangle }_{d+1}⊗{|W\rangle }^{2d}$, then we use the same local transformation to reduce the factors $\left({|\Psi_{0}\rangle }_{k}+{|\Psi_{3}\rangle }_{k}\right)$ in (48) into ${|\Psi_{0}\rangle }_{k}$ for each k=1,2,…,d. Then, we apply a sequence of non-local transformations between the pairs k,d+1 for k=1,2,…,d to transfer probability between states with indexes 01↔10 there, in such a way as to reach the coefficient $\frac{1}{\sqrt{d+1}}$ in each term. Finally, we revert the initial transformation for k=1,2,…,d+1, changing ${|\Psi_{0}\rangle }_{k}$ into $\left({|\Psi_{0}\rangle }_{k}+{|\Psi_{3}\rangle }_{k}\right)$. The final result is ${|W\rangle }^{2(d+1)}$, as is shown in Figure 9. Note how the entangling operations need to go through the overall pairs. It reflects the robustness of genuine entanglement in these states. By considering the expression for ${|W\rangle }^{2d}$ in (48), the following process gives the prescriptions to reach ${|W\rangle }^{2(d+1)}$ from ${|W\rangle }^{2d}$:

(55) $$\begin{array}{cc} {|\Psi_{0}\rangle }_{d+1}⊗{|W\rangle }^{2d}\;\begin{array}{c} H_{I}^{\left(3,k\right)}\\ k=1,2,\ldots ,d\\ \overset{\;}{\to }\\ \underset{s,s^{\prime }}{⨁}I_{s,s^{\prime }}^{0}⊕\underset{s^{\prime \prime },s^{\prime \prime \prime }}{⨁}H_{s^{\prime \prime },s^{\prime \prime \prime }}^{0,0,+}\\ s^{\prime }-s=4^{k-1},\\ s-4^{k-1},s^{\prime }-2\cdot 4^{k-1}\in \left\{3p\leq N|p\in N\right\}\\ s^{\prime \prime \prime }-s^{\prime \prime }=3\cdot 4^{k-1},\\ s^{\prime \prime }-4^{i-1},s^{\prime \prime \prime }-4^{i-1}\in \left\{3p\leq N|p\in N\right\},\\ k\neq i\in \{1,2,\ldots ,d\} \end{array} & \frac{{|\Psi_{0}\rangle }_{d+1}}{\sqrt{d}}⊗\sum_{i=1}^{d}{|\Psi_{4^{i-1}}\rangle }^{d},\\ \begin{array}{c} H_{IIa}^{\left(1,k,d+1\right)}\\ k=1,2,\ldots ,d\\ \overset{\;}{\to }\\ \underset{u,u^{\prime }}{⨁}I_{u,u^{\prime }}^{0}⊕{H_{0}^{0}\left(\delta_{k},\epsilon_{k}\right)}_{4^{k-1},4^{d}}\\ u=4^{i-1},u^{\prime }=u+4^{k-1}+4^{d}\\ k\neq i\in \{1,2,\ldots ,d\} \end{array} & \frac{1}{\sqrt{d+1}}\sum_{i=1}^{d+1}\overset{d+1}{\underset{\begin{array}{c} j=1\\ j\neq i \end{array}}{⨂}}{|\Psi_{0}\rangle }_{j}⊗{|\Psi_{1}\rangle }_{i},\\ \begin{array}{c} H_{I}^{\left(3,k\right)}\\ k=1,2,\ldots ,d,d+1\\ \overset{\;}{\to }\\ \underset{s,s^{\prime }}{⨁}I_{s,s^{\prime }}^{0}⊕\underset{s^{\prime \prime },s^{\prime \prime \prime }}{⨁}H_{s^{\prime \prime },s^{\prime \prime \prime }}^{0,0,+}\\ s^{\prime }-s=4^{k-1},\\ s-4^{k-1},s^{\prime }-2\cdot 4^{k-1}\in \left\{3p\leq N^{\prime }|p\in N\right\}\\ s^{\prime \prime \prime }-s^{\prime \prime }=3\cdot 4^{k-1},\\ s^{\prime \prime }-4^{i-1},s^{\prime \prime \prime }-4^{i-1}\in \left\{3p\leq N^{\prime }|p\in N\right\},\\ k\neq i\in \{1,2,\ldots ,d+1\} \end{array} & {|W\rangle }^{2(d+1)}. \end{array}$$

As before, Hadamard-like block operations for $H_{I}^{\left(3,k\right)}$ allow the states ${|W\rangle }^{2d}$ and ${|W\rangle }^{2(d+1)}$ to be switched, at the beginning and at the end, in terms of $|\Psi_{0}\rangle$ and $|\Psi_{1}\rangle$. The remarkable set of operations are obtained with $H_{IIa}^{\left(1,k,d+1\right)}$ to entangle the added state ${|\Psi_{0}\rangle }_{d+1}$ through the operations (38), which progressively transfer the probability to the state $|\Psi_{4^{d}}\rangle$, completing a state easily transformed into ${|W\rangle }^{2(d+1)}$ with the final set of operations. Additional exchange in the indexes is settled in the identity. The adequate set of $\epsilon_{k}$ values for each step of operations should fulfill the d+1 equations:

(56) $$\begin{array}{cc} g_{0} & \equiv \;0,\\ \sqrt{d} & =\;\sqrt{d+1}\left(\epsilon_{j}+\delta_{j}g_{j-1}\right),\;j=1,2,\ldots ,d,\\ g_{j} & =\;\delta_{j}-\epsilon_{j}g_{j-1},\;j=1,2,\ldots ,d-1,\\ \sqrt{d} & =\;\sqrt{d+1}\left(\delta_{d}-\epsilon_{d}g_{d-1}\right). \end{array}$$

**Figure 10:**
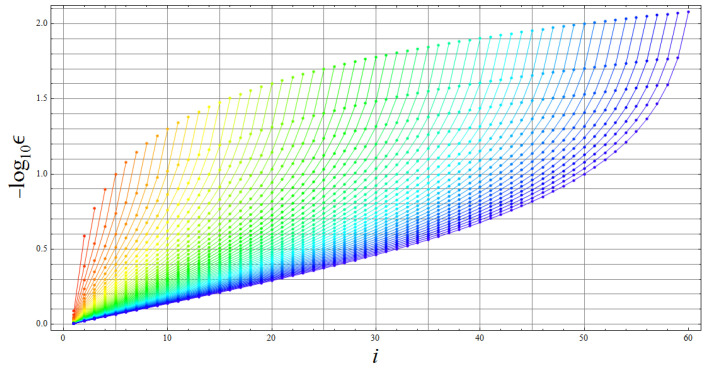
Solutions for $\epsilon_{i}$ in ${H_{0}^{0}\left(\delta_{i},\epsilon_{i}\right)}_{I,I^{\prime }}$ involved in the enlargement of ${|W\rangle }^{d}$ into ${|W\rangle }^{d+1}$ for values of d∈{2,…,60}.

These equations can be solved numerically for any *d*. Figure 10 shows the $-\log_{10}\epsilon_{i}$ solutions for d=1,2,…,60 by taking $\epsilon_{i},\delta_{i}>0$. Note that $\epsilon_{i}$ drops rapidly to zero when *d* and *i* grow.

### 6.5. Multipartite Entanglement and General States

In a previous subsection we described how to generate extended superposition using type *I* interactions. However, that process does not reach genuine entangled states. The use of type IIa,IIb interactions is mandatory to extend the entanglement as a set of operations involving elements of two pairs. Nevertheless, it is clear that many operations and combinations are necessary and possible.

For instance, by considering the permutation i,j,k from 1,2,3 and departing from the state ${|\Psi_{0}\rangle }^{2d}$, the process to reach an entangled state based on a complete combination from the basis elements for two correspondent pairs is as follows (note that the process is not unique). First, we apply a 2-local operation on the pair *s* and direction *i* followed by another on the pair $s^{\prime }$ in the direction *j*. A linear combination from four basis elements is obtained. Then, we apply a 4-local operation in the direction *k* and for pairs $s,s^{\prime }$, obtaining a state of eight terms. Finally, we again apply a 2-local operation on pair *s* in the direction *j*. At the end, we obtain the desired state of sixteen terms with the pairs $s,s^{\prime }$, genuinely entangled as it was seen in Table 4.

(57) $$\begin{array}{cc} {|\Psi_{0}\rangle }^{2d}\; & \begin{array}{c} H_{I}^{\left(i,s\right)}\\ \overset{\;}{\to }\\ O_{I}^{\left(i,\{s\}\right)} \end{array}\sum_{t\in \{0,i\}}\alpha_{t,0}^{s}|\Psi_{0,\ldots ,t,\ldots ,0,\ldots ,0}\rangle \begin{array}{c} H_{I}^{\left(j,s^{\prime }\right)}\\ \overset{\;}{\to }\\ O_{I}^{\left(j,\left\{s^{\prime }\right\}\right)} \end{array}\sum_{\begin{array}{c} t\in \{0,i\}\\ t^{\prime }\in \left\{0,j\right\} \end{array}}\alpha_{t,0}^{s}\alpha_{t^{\prime },0}^{s^{\prime }}|\Psi_{0,\ldots ,t,\ldots ,t^{\prime },\ldots ,0}\rangle ,\\ & \begin{array}{c} H_{IIa}^{\left(k,s,s^{\prime }\right)}\\ \overset{\;}{\to }\\ O_{IIa}^{\left(k,\left\{s,s^{\prime }\right\}\right)} \end{array}\sum_{\begin{array}{c} \epsilon ,\epsilon^{\prime }\in C_{4}\\ t\in \{0,i\}\\ t^{\prime }\in \left\{0,j\right\} \end{array}}\alpha_{t,0}^{s}\alpha_{t^{\prime },0}^{s^{\prime }}\beta_{\epsilon ,\epsilon^{\prime };t,t^{\prime }}^{s,s^{\prime }}\delta_{t,p_{s,k}^{t,\epsilon }}\delta_{t^{\prime },p_{s^{\prime },k}^{t^{\prime },\epsilon^{\prime }}}|\Psi_{0,\ldots ,\epsilon ,\ldots ,\epsilon^{\prime },\ldots ,0}\rangle ,\\ & \begin{array}{c} H_{I}^{\left(j,s\right)}\\ \overset{\;}{\to }\\ O_{I}^{\left(j,\{s\}\right)} \end{array}\sum_{\begin{array}{c} \chi ,\epsilon ,\epsilon^{\prime }\in C_{4}\\ t\in \{0,i\}\\ t^{\prime }\in \left\{0,j\right\} \end{array}}\alpha_{t,0}^{s}\alpha_{t^{\prime },0}^{s^{\prime }}\beta_{\epsilon ,\epsilon^{\prime };t,t^{\prime }}^{s,s^{\prime }}\alpha_{\chi ,\epsilon }^{s}\delta_{t,p_{s,k}^{t,\epsilon }}\delta_{t^{\prime },p_{s^{\prime },k}^{t^{\prime },\epsilon^{\prime }}}\delta_{\epsilon ,p_{s,j}^{t,\chi }}|\Psi_{0,\ldots ,\chi ,\ldots ,\epsilon^{\prime },\ldots ,0}\rangle , \end{array}$$

where $C_{4}=\left\{0,1,2,3\right\}$ and $p_{s,j}^{t,\epsilon }$ is the extension of the inverse exchange rule presented before $p_{s}\left(\epsilon \right)$, but specifying the rule *j* as a function of the direction of the interaction involved. The script t∈{0,i} is a label specifying each possible inverse. This means that if *j* is the characteristic direction of the interaction, then: $p_{s,j}^{0,i}=k=p_{s,j}^{0,k},p_{s,j}^{0,0}=0=p_{s,j}^{0,j}$ and $p_{s,j}^{i,i}=i=p_{s,j}^{i,k},p_{s,j}^{i,0}=j=p_{s,j}^{i,j}$. This single process could be improved using additional interactions to grow the spectrum of coefficients $\alpha_{\beta ,\alpha }^{s},\beta_{\beta ,\beta^{\prime };\alpha ,\alpha^{\prime }}^{s,s^{\prime }}$ in order to have a wider coverage of SU(4). In addition, it is clear the last process (or another alternative) should be repeated, varying one or two pairs in order to generate more complex entanglement. The question about how to generate a specific state under this procedure or to generate certain kind or level of entanglement is clearly open, mainly due to the poorly understood complexity to measure this property for large states in general.

## 7. Conclusions

Quantum gate array computation is based on the transformation of quantum states under certain universal operations. These operations are used to manipulate the information settled on quantum systems to simulate or reproduce computer processing, and normally use separable states as primary resources. Quantum systems involved—light or matter—are manipulated around entanglement generation in this kind of processing. Then, commonly involved interactions are non-local, implying that their parts become entangled when they are being manipulated. In the process, several slightly differentiated interactions are applied, each one with a different set of eigenvalues. This does not allow a common grammar to be set through the entire quantum information processing problem.

SU(2) decomposition provides a procedure not only to reduce control in the quantum manipulation states. Together, it provides a common language to address the evolution through several kinds of similar interactions in order to manage a wider processing. Upon the selection of a compatible basis, it allows the recovery of two-state processing despite the inclusion of the necessary entangling interactions. Although we developed the procedure for certain types of well-known interactions (i.e., Heisenberg–Ising and Dzyaloshinskii–Moriya), the process can be extended to other interactions and architectures (the arrangement of qubits under interaction) by the adequate selection of the basis on which dynamics should be expressed conveniently. In addition, it is advised that other configurations based on qudits are possible using alternative group decompositions to $SU(2^{2d})$ and SU(2). Finally, the development only proposes the change of quantum information grammar being used as function of the physical system in the deployment, preserving their applicability for most quantum information proposals in the literature.

Some applications of SU(2) decomposition are foreseen. It can be exploited in the quantum control of larger systems in which control schemes are not as well-developed as those of SU(2) dynamics. The previously established decomposition allows the establishment of exact control when blocks are reduced to the standard forms I,NOT,H, etc. The success of such strategy for exact control depends on the number of free parameters involved, which can be reached using a sequence of pulses instead of a single one, or otherwise time-dependent controlled parameters in the Hamiltonian, although the block structure is conserved. Similarly, optimal control in terms of energy or time can be achieved when procedures such as those in [9,10] are adapted to each block in the depicted structure. More ambitious ideas about the control of quantum processing such as the use of traveling waves, ion traps, resonant cavities, or superconducting circuits [37,38,39,40] could be adapted to the architectures presented here.

Note that the selectivity of pairing in the blocks is related to the arisen non-diagonal elements (i.e., with the interactions generating diagonal-off entries in all cases). This approach to quantum evolution will allow analytical control of the flow of quantum information in different adaptive geometrical arrangements. The use of more feasible external fields (other than stepwise fields) is compulsory, which is completely compatible with the current SU(2) reduction scheme [41].

In a related but not necessarily equivalent direction, selective block decomposition could be useful for unitary factorization in quantum gate design (e.g., that developed for the SU(4) case [26]), particularly for large dedicated gates involving the processing of several qubits. A current challenge in the mathematical arena is solving how to express certain $SU(2^{2d})$ matrices as a finite product in $U{\left(1\right)}^{2^{2d-1}-1}\times SU{\left(2\right)}^{2^{2d-1}}$, such as those developed here.

Finally, other applications in quantum processing could be engineered for multichannel quantum information storage, using certain subspaces to store differentiated information which could be processed simultaneously in other subspaces (e.g., in quantum image processing or quantum machine learning). Additional research should be developed to adapt this procedure to specific gate operations, and the translation of the most common algorithms into equivalent ones based on entangled resources like those shown here.

## Figures and Tables

**Table 1:** Basis pairs and Hamiltonian required to get the SU(2) block decomposition for case d=1.

Basis Arrangement	Hamiltonian
$\left\{\left\{|\beta_{00}\rangle ,|\beta_{01}\rangle \right\},\left\{|\beta_{11}\rangle ,|\beta_{10}\rangle \right\}\right\}$	$H=H_{0}+h_{01}\sigma_{0}⊗\sigma_{1}+h_{10}\sigma_{1}⊗\sigma_{0}+h_{23}\sigma_{2}⊗\sigma_{3}+h_{32}\sigma_{3}⊗\sigma_{2}$
$\left\{\left\{|\beta_{00}\rangle ,|\beta_{11}\rangle \right\},\left\{|\beta_{01}\rangle ,|\beta_{10}\rangle \right\}\right\}$	$H=H_{0}+h_{02}\sigma_{0}⊗\sigma_{2}+h_{20}\sigma_{2}⊗\sigma_{0}+h_{13}\sigma_{1}⊗\sigma_{3}+h_{31}\sigma_{3}⊗\sigma_{1}$
$\left\{\left\{|\beta_{00}\rangle ,|\beta_{10}\rangle \right\},\left\{|\beta_{01}\rangle ,|\beta_{11}\rangle \right\}\right\}$	$H=H_{0}+h_{03}\sigma_{0}⊗\sigma_{3}+h_{30}\sigma_{3}⊗\sigma_{0}+h_{12}\sigma_{1}⊗\sigma_{2}+h_{21}\sigma_{2}⊗\sigma_{1}$

**Table 2:** Rows generated and free parameters in each interaction considered in the text.

Hamiltonian	Entries Type	Entries by Column/Row	Parameters by Entry
$H_{0}$	Diagonal	1	D≤3
$H_{l_{i}}$	Non-diagonal	*d*	2
$H_{{\mathrm{nl}}_{i}}^{c}$	Diagonal	1	*d*
$H_{{\mathrm{nl}}_{i}}^{nc}$	Non-diagonal	$\frac{1}{2}d\left(d-1\right)$	4
$H_{{\mathrm{cnl}}_{i}}^{c}$	Non-diagonal	*d*	2
$H_{{\mathrm{cnl}}_{i}}^{nc}$	Non-diagonal	d(d−1)	4
$H_{I}$	2 × 2 block	2	2+Dd≤2+3d
$H_{IIa,b}$	2 × 2 block	2	4+Dd≤4+3d
$H_{III}$	2 × 2 block	2	2+Dd≤2+3d

**Table 3:** Values of $c_{j_{s},j_{d+s}}^{i_{s},k_{s}}$ for all exchange scripts in $H_{I},H_{IIa,b},H_{III}$. i,j,k is an even permutation of 1,2,3.

$\left(j_{s},j_{s+d}\right)$	$\left(i_{s},k_{s}\right)$	$c_{j_{s},j_{d+s}}^{i_{s},k_{s}}$	$\left(i_{s},k_{s}\right)$	$c_{j_{s},j_{d+s}}^{i_{s},k_{s}}$	$\left(i_{s},k_{s}\right)$	$c_{j_{s},j_{d+s}}^{i_{s},k_{s}}$	$\left(i_{s},k_{s}\right)$	$c_{j_{s},j_{d+s}}^{i_{s},k_{s}}$
(0,2)	(0,2)	−i	(2,0)	*i*	(1,3)	*i*	(3,1)	−i
(2,0)	(0,2)	*i*	(2,0)	−i	(1,3)	*i*	(3,1)	−i
(0,j≠2)	(0,j)	1	(j,0)	1	(i,k)	$-{\left(-1\right)}^{\delta_{2k}}$	(k,i)	$-{\left(-1\right)}^{\delta_{2k}}$
(j≠2,0)	(0,j)	1	(j,0)	1	(i,k)	${\left(-1\right)}^{\delta_{2k}}$	(k,i)	${\left(-1\right)}^{\delta_{2k}}$
2∈(j,k)	(j,k)	−i	(k,j)	*i*	(0,i)	$-i{\left(-1\right)}^{\delta_{2k}}$	(i,0)	$i{\left(-1\right)}^{\delta_{2k}}$
2∈(k,j)	(j,k)	*i*	(k,j)	−i	(0,i)	$-i{\left(-1\right)}^{\delta_{2k}}$	(i,0)	$i{\left(-1\right)}^{\delta_{2k}}$
(1,3)	(1,3)	1	(3,1)	1	(0,2)	−1	(2,0)	−1
(3,1)	(1,3)	1	(3,1)	1	(0,2)	1	(2,0)	1

**Table 4:** Bipartite concurrence $C^{2}\left({\mathrm{Tr}}^{S}\left(\rho_{IJ}\right)\right)$ for several subsystems in the SU(2) mixing of some pairs of GBS basis states.

Case	*S*	$C^{2}\left({\mathrm{Tr}}^{S}\left(\rho_{IJ}\right)\right)$
(a) $|\phi_{\mathrm{IJ}}^{1}\rangle$	$\left[s\notin \left\{k^{\prime },k^{\prime }+d\right\}\right]$	1
(b) $|\phi_{\mathrm{IJ}}^{1}\rangle$	$\left[s\in \left\{k^{\prime },k^{\prime }+d\right\}\right]$	$1-\sin^{2}\theta {\left(\cos\phi^{\prime }\delta_{0,i_{k^{\prime }}\cdot j_{k^{\prime }}}+{\left(-1\right)}^{\epsilon_{i_{k^{\prime }}j_{k^{\prime }}j}}\left(1-\delta_{0,i_{k^{\prime }}\cdot j_{k^{\prime }}}\right)\sin\phi^{\prime }\right)}^{2}$
(c) $|\phi_{\mathrm{IJ}}^{1}\rangle$	$\left[k^{\prime },k^{\prime }+d\right]$	0
(d) $|\phi_{\mathrm{IJ}}^{2}\rangle$	$\left[k^{\prime },k^{\prime }+d\right]$	$\sin^{2}\theta$
(e) $|\phi_{\mathrm{IJ}}^{2}\rangle$	$\left[k^{\prime },k^{\prime \prime }\right]$	$\frac{3}{2}-\frac{1}{2}\sin^{2}\theta \left(\cos^{2}\phi^{\prime }\delta_{i_{k^{\prime }}j_{k^{\prime }}}\delta_{i_{k^{\prime \prime }}j_{k^{\prime \prime }}}+\sin^{2}\phi^{\prime }\left(1-\delta_{i_{k^{\prime }}j_{k^{\prime }}}\delta_{i_{k^{\prime \prime }}j_{k^{\prime \prime }}}\right)\right)$

## Appendix A

The Appendix is divided into four parts to develop a more detailed understanding of some critical aspects in the paper. The first is the motivation of the Hamiltonian used here, which is expressed in terms of Pauli operators (or Pauli matrices) together with the identity. Because another central aspect is the terminology around the group theory, the second Appendix brielfy explains some terms and developments used in the paper, always centered in the *special unitary groups*, SU(n). Special attention is given to the concept of the *groups product*, which is central in the paper. The third Appendix explains the GBS basis developed by Sych and Leuchs [22]—a set of quantum states with partial entanglement setting a basis that is useful for our development. Because this paper contains sections which may make it difficult to understand the generality of the proposal for larger values of *d*, the fourth Appendix presents the two more basic examples: d=1, which has already been indirectly presented in the literature [11]; and d=2, which comprises aspects not encountered with d=1, while they are present for the d>1 cases.

### Appendix A.1. Generic Hamiltonian Expressed in Terms of Pauli Operators

The Hamiltonian for the interaction between a magnetic object and an external magnetic field is $-\vec{\mu }\cdot \vec{B}$, where $\vec{\mu }$ is the dipole moment of the object. For quantum particles, this dipole moment is precisely the spin, commonly expressed in terms of the Pauli operators $\vec{\sigma }=\left(\sigma_{x},\sigma_{y},\sigma_{z}\right)$ as $\vec{\mu }=\frac{ge}{2m}\vec{s}=\frac{ge\hbar }{4m}\vec{\sigma }$. Thus, the interaction reads $H=-\vec{\sigma }\cdot \vec{B}$ by absorbing the physical constants $\frac{\hbar }{2}$ in $\vec{B}$.

For quantum magnetic systems, the most common interaction between two level systems is the Heisenberg–Ising interaction. This interaction is a low-order approximation for the far-field interaction between two magnetic dipoles in terms of the spin particles: $\vec{s_{1}}\cdot J\cdot \vec{s_{2}}$ or $\vec{\sigma_{1}}\cdot J\cdot \vec{\sigma_{2}}$, when the spins $\vec{s_{i}}=\frac{\hbar }{2}\vec{\sigma_{i}}$ are expressed in terms of Pauli operators by absorbing the factors $\frac{\hbar }{2}$ in J, which is generally a tensor. When J becomes diagonal, we get the anisotropic Heisenberg–Ising interaction. Moreover, if those diagonal elements become equal, the interaction becomes isotropic.

In the context of this work, another kind of interaction appears—the Dzyaloshinskii–Moriya interaction, which is a contribution to the total magnetic exchange interaction between two neighboring magnetic spins [24,25]: $H=\vec{D}\cdot \left(\vec{\sigma_{1}}\times \vec{\sigma_{2}}\right)$. $\vec{D}$ is a vector expressed in terms of the sources’ orientation. Clearly, this Hamiltonian will contain terms such as $D_{1}\sigma_{1_{y}}⊗\sigma_{2_{z}},D_{2}\sigma_{1_{z}}⊗\sigma_{2_{x}},D_{3}\sigma_{1_{x}}⊗\sigma_{2_{y}}$, where the script denotes the part and the subscript denotes the component (tensor product symbol ⊗ is introduced to remark that the product is between the spins of different quantum objects).

It is clear from the previous examples that the interactions contain terms involving one or two Pauli operators from the different physical parts. Although most terms with two spins appear there because interactions directly occur between a pair of physical objects, the motivation to include all classes of products of Pauli operators in the Hamiltonian (1) is to consider the most extensive types of interaction Notably, in the development of this work, precisely the previous interactions set a special kind of interactions, making the SU(2) decomposition possible Particularly we should note that each term in the Heisenberg–Ising and Dzyaloshinskii–Moriya interactions are only able to generate entangling operations between the pair of objects involved. However, extended entanglement could be generated by including many of those interactions between other pairs, as in the case of Ising chains. A conclusion from this paper is that non-physical terms containing more than two spin factors in the interaction could automatically generate more extended and inclusively genuine entanglement. For instance, for 2d qubits, one term containing 2d factors in one term of the Hamiltonian $\sigma_{1_{z}}⊗\sigma_{2_{z}}⊗\ldots \sigma_{{2d}_{z}}$, which can generate genuine entanglement in fewer steps than are necessary in Section 6. Note also that powers or additional factors for each operator are not necessary because of their algebraic properties (any product of them for each part can be reduced to only one operator until unitary factors).

Although these examples are for magnetic systems, these kinds of Hamiltonians are not exclusive to those systems. Thus, for instance, the dipole interaction for a two-level system (an atom or ion restricted to excitation between two energy levels) in a radiation trap, particles in a double-well potential, etc., also have Hamiltonians expressed in terms of the Pauli matrices because they are the basis of SU(2) dynamics, common for all two-level systems (see A.2). Finally, there is a mathematical reason for the form of Hamiltonian (1). For all two-level quantum systems, dynamics are ruled by transformations given by elements of the *unitary group of order 2*, U(2) (see Appendix A.2), as solutions from the Schödinger equation for the evolution operator. In group theory, those elements can be depicted as the exponential of the *generators* of the group, which are precisely the Pauli operators defining an associated Lie algebra: $\exp(i\sum_{k\in 0,1,2,3}\alpha_{k}\sigma_{k})$ (see Appendix A.2). For composed systems, the set of generators (see Appendix A.2) and the basis elements (see Appendix A.3) for their dynamics are precisely the different products between the generators for each part: $\left\{{⨂}_{j=1}^{d}\sigma_{j_{k}}|k\in 0,1,2,3\right\}$. Thus, through the Schrödinger equation, we can identify the exponent with the Hamiltonian in (1), thus representing the most general Hamiltonian for the current system composed of 2d two-level quantum systems.

### Appendix A.2. Group Theory Basics in the Context of the SU(2) Decomposition

In the current Appendix we deliver a minimum of the group theory context to understand some aspects in this work. For a deeper treatment, [42,43] is a modern introductory resource. We begin by remarking on the notion of a *group*. It is a set *G* of elements $g_{i}\in G$ together with a defined product operation · fulfilling the properties: (a) *Closure*: $g_{1}\cdot g_{2}\in G$ for all $g_{1},g_{2}\in G$ (otherwise with a defined map: G×G→G); (b) *Associativity*: $g1\cdot \left(g_{2}\cdot g_{3}\right)=\left(g1\cdot g_{2}\right)\cdot g_{3}$ for all $g_{1},g_{2},g_{3}\in G$; (c) *Identity element*: there is a unique e∈G such that g·e=e·g=g for all g∈G; (d) *Inverse*: for each g∈G there exist $g^{-1}\in G$ such that $g\cdot g^{-1}=g^{-1}\cdot g=e$. If $G^{\prime }\subset G$ is itself a group, then we say $G^{\prime }$ is a subgroup of *G*.

In two-level quantum systems we are interested in states defined in terms of a superposition of two orthonormal states $|\psi_{0}\rangle ,|\psi_{1}\rangle$: $|\psi \rangle =\alpha_{0}|\psi_{0}\rangle +\alpha_{1}|\psi_{1}\rangle$. Equivalently, we can use the matrix notation:

(A1) $$\begin{array}{ccc} \psi & = & \left(\begin{array}{c} \alpha_{0}\\ \alpha_{1} \end{array}\right) \end{array}$$

to depict such states. Those states have a time evolution $|\psi (t)\rangle$ via the evolution operator U(t) obeying the Schrödinger Equation (2) in terms of the Hamiltonian operator, *H*. U(t) is an operator acting on the original state to evolve it: $|\psi (t)\rangle =U\left(t\right)|\psi \rangle$. It fulfills: (a) the outcome $|\psi (t)\rangle$ belongs to the set depicted by a superposition of $|\psi_{0}\rangle ,|\psi_{1}\rangle$; and (b) the norm of the new state is preserved: $\langle \psi (t)|\psi (t)\rangle =\langle {\psi |U\left(t\right)}^{†}U\left(t\right)|\psi \rangle =\langle \psi |\psi \rangle$, then $U{\left(t\right)}^{†}=U{\left(t\right)}^{-1}$. In the context of the Dirac notation, the dual $U{\left(t\right)}^{†}$ is another operator related with U(t). The evolution operator should clearly fulfill: (i) U(0)=I, the identity operator which leaves $|\psi \rangle$ without change; and (ii) $U\left(t_{2}\right)U\left(t_{1}\right)=U\left(t_{1}+t_{2}\right)$. Then, the reader can easily note that the set of operators U(t) for different values of *t* should form a group with the property $U{\left(t\right)}^{†}=U{\left(t\right)}^{-1}$. This group is said the *unitary group of order 2*, U(2). Similarly, we can define a n-level system, where the evolution operators define the *unitary group of order n*, U(n).

For U(2), because the action of U(t) on $|\psi \rangle$ is again a linear combination of $|\psi_{0}\rangle ,|\psi_{1}\rangle$, then we know that:

(A2) $$\begin{array}{c} U\left(t\right)=\sum_{i,j=0}^{1}u_{i,j}|\psi_{i}\rangle \langle \psi_{j}|\;\mathrm{or}:\;U\left(t\right)=\left(\begin{array}{cc} u_{00} & u_{01}\\ u_{10} & u_{11} \end{array}\right) \end{array}$$

(in the following, for simplicity, we will adopt both representations as equivalent). Because of the norm definition for quantum states, we know that:

(A3) $$\begin{array}{c} U{\left(t\right)}^{†}=\sum_{i,j=0}^{1}u_{i,j}^{*}|\psi_{j}\rangle \langle \psi_{i}|\;\mathrm{or}:\;U{\left(t\right)}^{†}=\left(\begin{array}{cc} u_{00}^{*} & u_{10}^{*}\\ u_{01}^{*} & u_{11}^{*} \end{array}\right)=\frac{1}{\left|U(t)\right|}\left(\begin{array}{cc} u_{11} & -u_{01}\\ -u_{10} & u_{00} \end{array}\right)=U{\left(t\right)}^{-1}, \end{array}$$

which clearly shows that entries for U(t) should fulfill the restrictions: $u_{11}\;=\;u_{00}^{*}e^{2i\phi },u_{10}\;=\;-\;u_{01}^{*}e^{2i\phi },|u_{00}{|}^{2}+{\left|u_{01}\right|}^{2}=1$ (then, $\left|U\left(t\right)\right|=e^{2i\phi }$, with ϕ∈R arbitrary):

(A4) $$\begin{array}{c} U\left(t\right)=\left(\begin{array}{cc} u_{00} & u_{01}\\ -e^{2i\phi }u_{01}^{*} & e^{2i\phi }u_{00}^{*} \end{array}\right)=e^{i\phi }\left(\begin{array}{cc} e^{-i\phi }u_{00} & e^{-i\phi }u_{01}\\ -{\left(e^{-i\phi }u_{01}\right)}^{*} & {\left(e^{-i\phi }u_{00}\right)}^{*} \end{array}\right)\equiv e^{i\phi }\tilde{U}\left(t\right). \end{array}$$

We advise in the last structure that both $e^{i\phi }$ and $\tilde{U}\left(t\right)$ form groups separately. The set of numbers $e^{i\phi }$ are clearly the U(1) group under the standard multiplication of complex numbers. We skip the demonstration that $\tilde{U}\left(t\right)$ with the standard matrix multiplication forms a group, which is trivial for the associativity, identity element, and inverse properties. Demonstration for the closure property is direct. We note that elements in this group fulfill the property $|\tilde{U}\left(t\right)|=1$. This group is said to be the *special unitary group*, SU(2). Normally, in quantum mechanics we select U(t)∈SU(2) because the phase $e^{i\phi }$ is non-physical. For this reason we drop the tilde indistinctly. Because (A4), we say that U(2) is the *direct product* of U(1) and SU(2): U(2)=U(1)×SU(2) (the reader is advised that the term product is not due to the scalar product underlying in (A4), but instead to a pairing in terms of the Cartesian product of the elements of each group. For a formal definition, consult [42,43]; this concept will be relevant later). SU(2) is clearly a subgroup of U(2).

Another important property of the SU(2) group is that any element of it can be written as a linear combination of the Pauli matrices (this aspect is widely used in the text). This means that they are a basis for matrices in SU(2) (then also for U(2)). In fact:

(A5) $$\begin{array}{c} \tilde{U}\left(t\right)=ℜ\left(e^{-i\phi }u_{00}\right)\sigma_{0}+iℑ\left(e^{-i\phi }u_{01}\right)\sigma_{1}+iℜ\left(e^{-i\phi }u_{01}\right)\sigma_{2}+ℑ\left(e^{-i\phi }u_{00}\right)\sigma_{3}, \end{array}$$

where *ℜ* and *ℑ* are the real and imaginary part functions (in addition, note we are using here indistinctly the notation $\sigma_{0}=I,\sigma_{1}=\sigma_{x}=X,\sigma_{2}=\sigma_{y}=Y,\sigma_{3}=\sigma_{z}=Z$, although in the text they have several meanings as the basis of the different SU(2) elements appearing there). Moreover, in several parts of the text, the following property is used, derived from the algebra fulfilled by Pauli matrices (obtained from the fact: ${\left(n\cdot \vec{\sigma }\right)}^{2s}=1,s\in Z$):

(A6) $$\begin{array}{c} e^{i\alpha n\cdot \vec{\sigma }}=\cos\alpha \;\sigma_{0}+i\sin\alpha \;n\cdot \vec{\sigma }=\left(\begin{array}{cc} \cos\alpha +in_{3}\sin\alpha & i\sin\alpha (n_{1}-in_{2})\\ i\sin\alpha (n_{1}+in_{2}) & \cos\alpha -in_{3}\sin\alpha \end{array}\right), \end{array}$$

where n is a unitary vector with real components. From the last expression, it is easy to demonstrate that $|e^{i\alpha n\cdot \vec{\sigma }}|=1$, so by comparing with (A4), it is advisable that (A6) is a parametrization for the elements in SU(2). Moreover, from the Baker–Campbell–Hausdorff formula [42,43]:

(A7) $$\begin{array}{c} e^{i(\phi \sigma_{0}+\alpha n\cdot \vec{\sigma })}=e^{i\phi }e^{i\alpha n\cdot \vec{\sigma }}. \end{array}$$

Then, it is said that $\sigma_{1},\sigma_{2},\sigma_{3}$ are the *generators* of SU(2), while $\sigma_{0},\sigma_{1},\sigma_{2},\sigma_{3}$ are the generators of U(2). This fact was mentioned in Appendix A.1 to suggest the generality of Hamiltonian (1), although some steps remain to arrive at the $SU(2^{2d})$ group.

The reader can note that (13) and (29) adjust to those structures, and thus the blocks in the decomposition belong to U(1)×SU(2). Similar arguments in group theory show that U(n)=U(1)×SU(n), where SU(n) is the *special unitary group of order n*, the group of unitary matrices $U^{†}=U^{-1}$ with determinant equal to 1, |U|=1. Although there are generators for such groups, the development of the article shows that the combination of 2d two-level quantum systems requires evolution matrices in $U(2^{2d})$ (or in $SU(2^{2d})$). As a result of the decomposition, we show that the evolution matrices belonging to such groups form the product group $U{\left(2\right)}^{2^{2d-1}}$ (while the Hilbert space of the quantum states is decomposed into the direct sum of $2^{2d-1}$ two-level subspaces of dimension 2). Precisely, the elimination of the term $h_{\left\{00\ldots 0\right\}}$ in the Hamiltonian induces directly in (14) that $U\in SU(2^{2d})$. In addition, the SU(2) decomposition shows in this case that $U\in U{\left(1\right)}^{2^{2d-1}-1}\times SU{\left(2\right)}^{2^{2d-1}})$ (due to the dependence of one U(1)-term from the remaining). The reader should consult the formal definition of a *direct product* in [42,43].

### Appendix A.3. Generalized Bell States Basis in Context

GBS states (generalized Bell states) as introduced by Sych and Leuchs [22] (16) were expressed in terms of Pauli operators. That original expression is highly convenient for the development of the current work because it allows it to be easily connected with the form of the Hamiltonian (1) in the same terms, allowing the important result to be easily obtained (18). Nevertheless, a more simple expression could be given for the understanding of such states. In fact, it is easy to note that each element in the GBS basis for 2d qubits can be written as:

(A8) $$\begin{array}{ccc} |\Psi_{I_{4}^{d}}\rangle & = & \overset{d}{\underset{j=1}{⨂}}|\Psi_{I_{4,j}^{d}}\rangle =|\Psi_{i_{1}}\rangle ⊗|\Psi_{i_{2}}\rangle ⊗\ldots ⊗|\Psi_{i_{d}}\rangle , \end{array}$$

where $I_{4,j}^{d}=i_{j}$, and $|\Psi_{2\gamma +(\gamma ⊕\delta )}\rangle =|\beta_{\gamma ,\delta }\rangle$ or $|\Psi_{i}\rangle =|\beta_{\left(\frac{i}{2}\mathrm{mod}2\right),\left(i-2\left(\frac{i}{2}\mathrm{mod}2\right)\right)⊕(\frac{i}{2}\mathrm{mod}2})\rangle$, the well-known single Bell states (in the last expressions, ⊕ represents the module-2 sum). Thus, each element of the GBS basis is in reality a tensor product of *d* Bell states identified through their scripts in base-4. These states are 2-separable (meaning the smallest separable subsystems still contains two entangled parts). Thus, when we apply a Hadamard-like operation involving only one script (Type I or III interactions), we are consequently able to convert the involved Bell state into a separable state. When we look at this version of the GBS basis, it is clearer why Type I and III interactions become in entangling or unentagling operations on only one correspondent pair. Both types of operations actually resemble the effect on two-qubits processing with SU(4) operations such as those developed in [11], while the remaining system is not involved. Only the Type II operations provide more extended entangling properties.

### Appendix A.4. Illustrative Examples of SU*(2)* Decomposition

In the following two subsections, we develop examples of the aspect of the evolution operators for the specific cases d=1 and d=2. The latter case is of special importance because it depicts how Type II interactions extend the entanglement, as shown in Section 6.

#### Appendix A.4.1. Case *d* = 1

This case has been developed in the literature [11,13]. In the current context, only Type I and Type III interactions are possible (because there is only one correspondent pair). The corresponding GBS basis has four elements: $|\Psi_{0}\rangle ,|\Psi_{1}\rangle ,|\Psi_{2}\rangle ,|\Psi_{3}\rangle$, the Bell states precisely. In the next expressions, we assume a lexicographic order in the components of the basis, so any arrangement of them is supposed (in contrast to how it was considered in (14)). The Hamiltonian $H_{I}$ contains at the most five terms:

(A9) $$\begin{array}{c} H_{I}=\sum_{m=1}^{3}h_{m,m}\sigma_{1_{m}}⊗\sigma_{2_{m}}+h_{k,0}\sigma_{1_{k}}+h_{0,k}\sigma_{2_{k}}, \end{array}$$

where *k* is the direction of the local interaction. Te Hamiltonian $H_{III}$ (with the crossed interaction in the direction *k*) also contains utmost five terms. If i,j,k is an even permutation of 1,2,3:

(A10) $$\begin{array}{c} H_{III}=\sum_{m=1}^{3}h_{m,m}\sigma_{1_{m}}⊗\sigma_{2_{m}}+h_{i,j}\sigma_{1_{i}}⊗\sigma_{2_{j}}+h_{j,i}\sigma_{1_{j}}⊗\sigma_{2_{i}}. \end{array}$$

Although it is an special case accepting the combination of the two Hamiltonians (Table 1), we set them separately. Because of the space and complexity, we do not express $U_{k}\left(t\right)$ in terms of the original coefficients in (A9) and (A10). In any case, formulas (31)–(37) are sufficiently efficient to reproduce the entries of each Hamiltonian. Instead, after expressing both Hamiltonians in the GBS basis, they become (as in (28)):

(A11) $$\begin{array}{ccc} H_{1}= & \left(\begin{array}{cccc} h_{11}^{1} & h_{12}^{1} & 0 & 0\\ h_{12}^{1*} & h_{22}^{1} & 0 & 0\\ 0 & 0 & h_{11}^{2} & h_{12}^{2}\\ 0 & 0 & h_{12}^{2*} & h_{22}^{2} \end{array}\right)=S_{H_{0,1}}⊕S_{H_{2,3}} & ⟹\;U_{1}\left(t\right)=S_{U_{0,1}}⊕S_{U_{2,3}},\\ H_{2}= & \left(\begin{array}{cccc} h_{11}^{1} & 0 & h_{12}^{1} & 0\\ 0 & h_{11}^{2} & 0 & h_{12}^{2}\\ h_{12}^{1*} & 0 & h_{22}^{1} & 0\\ 0 & h_{12}^{2*} & 0 & h_{22}^{2} \end{array}\right)=S_{H_{0,2}}⊕S_{H_{1,3}} & ⟹\;U_{2}\left(t\right)=S_{U_{0,2}}⊕S_{U_{1,3}},\\ H_{3}= & \left(\begin{array}{cccc} h_{11}^{1} & 0 & 0 & h_{12}^{1}\\ 0 & h_{11}^{2} & h_{12}^{2} & 0\\ 0 & h_{12}^{2*} & h_{22}^{2} & 0\\ h_{12}^{1*} & 0 & 0 & h_{22}^{1} \end{array}\right)=S_{H_{0,3}}⊕S_{H_{1,2}} & ⟹\;U_{3}\left(t\right)=S_{U_{0,3}}⊕S_{U_{1,2}}, \end{array}$$

where the superscript *i* in $h_{mn}^{i}$ points out the consecutive number of blocks and $S_{H_{I,I^{\prime }}}$ fulfills the syntactic notation followed in (28) and (29). We will exploit this notation in the following section for simplicity, where the matrix notation will become hardly extensive.

#### Appendix A.4.2. Case *d* = 2

We develop two cases for the case d=2. The first considers the Type I interaction and the second pertains to the Type IIa interaction. The last case involves a different situation not appearing in d=1: the possibility of generating extended entanglement among the four qubits involved in this case.

By considering the four qubits under the Type I interaction with the local interaction terms on the pair $k^{\prime }=2$ in the direction *j* in (30), the Hamiltonian has the form:

(A12) $$\begin{array}{c} H_{I}=\sum_{m=1}^{3}h_{m,0,m,0}\sigma_{1_{m}}⊗\sigma_{3_{m}}+\sum_{m=1}^{3}h_{0,m,0,m}\sigma_{2_{m}}⊗\sigma_{4_{m}}+h_{0,j,0,0}\sigma_{2_{j}}+h_{0,0,0,j}\sigma_{4_{j}}. \end{array}$$

There are 16 GBS elements in the basis: $|\Psi_{0,0}\rangle ,|\Psi_{0,1}\rangle ,\ldots ,|\Psi_{0,3}\rangle ,|\Psi_{1,0}\rangle ,|\Psi_{1,1}\rangle ,\ldots ,|\Psi_{3,3}\rangle$. Interaction generates exchanges between the GBS basis elements as follows (forming eight blocks but only two types of them). If i,j,k is a permutation of 1,2,3 with i<k, then: (a) $|\Psi_{m,0}\rangle ⟷|\Psi_{m,j}\rangle$; (b) $|\Psi_{m,i}\rangle ⟷|\Psi_{m,k}\rangle$, with m=0,…,3. Due to the extension, we do not write matrix expressions as in the case d=1 (which already settled an illustrative orientation to the reader). Instead, we use the notation of direct sum for block matrices as before. Thus, remembering the scripts are numbers becoming from the base-4 scripts in the GBS basis, $|\Psi_{a,b}\rangle =|\Psi_{I}\rangle$ with I=a+4b∈0,1,…,15, the decomposition for the evolution operator will become for $U_{j}\left(t\right)$:

(A13) $$\begin{array}{cc} U_{j}\left(t\right)\; & =\left(\overset{3}{\underset{m=0}{⨁}}S_{U_{m,m+4j}}\right)⊕\left(\overset{3}{\underset{m=0}{⨁}}S_{U_{m+4i,m+4k}}\right),\\ & \mathrm{then}:\\ & U_{1}\left(t\right)=S_{U_{0,4}}⊕S_{U_{1,5}}⊕S_{U_{2,6}}⊕S_{U_{3,7}}⊕S_{U_{8,12}}⊕S_{U_{9,13}}⊕S_{U_{10,14}}⊕S_{U_{11,15}},\\ & U_{2}\left(t\right)=S_{U_{0,8}}⊕S_{U_{1,9}}⊕S_{U_{2,10}}⊕S_{U_{3,11}}⊕S_{U_{4,12}}⊕S_{U_{5,13}}⊕S_{U_{6,14}}⊕S_{U_{7,15}},\\ & U_{3}\left(t\right)=S_{U_{0,12}}⊕S_{U_{1,13}}⊕S_{U_{2,14}}⊕S_{U_{3,15}}⊕S_{U_{4,8}}⊕S_{U_{5,9}}⊕S_{U_{6,10}}⊕S_{U_{7,11}}. \end{array}$$

These exchanges only involve qubits in the same correspondent pair, so they cannot extend the entanglement beyond this pair. Additionally, we remark that the first four blocks have the same form, as do the last four. Here, only two different types of blocks exist.

We develop the case d=2 for a Type IIa interaction involving additional non-local and non-crossed interactions between the pairs $k^{\prime }=1$ and $k^{\prime \prime }=2$ (note that the situation will be similar for the cases with d>2). Assuming the interaction in the direction *j* and in (30), the Hamiltonian becomes:

(A14) $$\begin{array}{cc} H_{IIa}= & \sum_{m=1}^{3}h_{m,0,m,0}\sigma_{1_{m}}⊗\sigma_{3_{m}}+\sum_{m=1}^{3}h_{0,m,0,m}\sigma_{2_{m}}⊗\sigma_{4_{m}}+\\ & h_{j,j,0,0}\sigma_{1_{j}}⊗\sigma_{2_{j}}+h_{j,0,0,j}\sigma_{1_{j}}⊗\sigma_{4_{j}}+h_{0,j,j,0}\sigma_{2_{j}}⊗\sigma_{3_{j}}+h_{0,0,j,j}\sigma_{3_{j}}⊗\sigma_{4_{j}}. \end{array}$$

Then, there exist eight types of exchanges and blocks (i,j,k is a permutation of 1,2,3): (a) $|\Psi_{0,0}\rangle ⟷|\Psi_{j,j}\rangle$; (b) $|\Psi_{0,i}\rangle ⟷|\Psi_{j,k}\rangle$; (c) $|\Psi_{0,j}\rangle ⟷|\Psi_{j,0}\rangle$; (d) $|\Psi_{0,k}\rangle ⟷|\Psi_{j,i}\rangle$; (e) $|\Psi_{i,0}\rangle ⟷|\Psi_{k,j}\rangle$; (f) $|\Psi_{i,i}\rangle ⟷|\Psi_{k,k}\rangle$; (g) $|\Psi_{i,j}\rangle ⟷|\Psi_{k,0}\rangle$; (h) $|\Psi_{i,k}\rangle ⟷|\Psi_{k,i}\rangle$. In this case, all blocks will become different, but it is not a general situation when *d* grows. As before $|\Psi_{a,b}\rangle =|\Psi_{I}\rangle$, with I=a+4b∈0,1,…,15. Then, the evolution operator can be written as:

(A15) $$\begin{array}{c} U_{j}\left(t\right)=S_{U_{0,j+4j}}⊕S_{U_{4i,j+4k}}⊕S_{U_{4j,j}}⊕S_{U_{4k,j+4i}}⊕S_{U_{i,k+4j}}⊕S_{U_{i+4i,k+4k}}⊕S_{U_{i+4j,k}}⊕S_{U_{i+4k,k+4i}}, \end{array}$$

noting that the exchange involving two scripts implies the generation of entanglement between the two correspondent pairs (i.e., among the four qubits as a whole). In this case, eight blocks are different (but not necessarily independent,—because there are only 11 parameters free, including time *t*).

These two examples show in detail how the SU(2) decomposition is established. For cases d>2, the situation becomes similar and they are easily understood using the last synthetic notation in terms of direct sums of blocks. It should finally be remarked that formulas (30) and (37) are computationally useful and efficient to connect the original Hamiltonian coefficients with the entries for each block.
